# Subsurface messenger for the annual maximum lifetime maximum intensity of tropical cyclones in the western North Pacific

**DOI:** 10.1038/s41467-026-72770-5

**Published:** 2026-05-07

**Authors:** Xinning Ni, Yu Zhang, Wei Wang

**Affiliations:** 1 Key Laboratory of Physical Oceanography and Frontiers Science Center for Deep Ocean Multispheres and Earth System/Academy of Future Ocean, Ocean University of China, Qingdao, China; 2 Laboratory for Ocean Dynamics and Climate, Qingdao Marine Science and Technology Center, Qingdao, China

**Keywords:** Physical oceanography, Natural hazards

## Abstract

Given the large number of record-breaking tropical cyclones (TCs) in recent years, there is a pressing need to investigate how strong TCs respond to climate change. Here we find that the annual maximum lifetime maximum intensity (LMI) of TC in the western North Pacific is strongly correlated with the temperature of a subsurface water mass, exhibiting a multi-decadal V-shaped structure in the past four decades. This water mass originally forms and is subducted in the eastern North Pacific under the center of the North Pacific High (NPH). It is then transported along a subsurface path over approximately four years to the western boundary. Correspondingly, the annual maximum LMI can be predicted several years in advance based on the intensity of NPH. We propose a mechanism in which the highly variable heat content of the subsurface water mass modulates the under-storm sea surface temperature through TC-induced mixing and upwelling process.

## Abstract (web summary)

This study shows that the western North Pacific tropical cyclone peak intensity correlates with a subsurface water mass formed under the North Pacific High in the eastern North Pacific, allowing tropical cyclone intensity to be predicted years ahead.

## Introduction

Tropical cyclones (TCs) rank among the most devastating natural hazards, inflicting severe casualties and massive economic losses across tropical and subtropical regions^1–7^. Consequently, accurate and timely TC forecasting becomes crucial for effective disaster risk reduction. Under global warming, the trends of TCs activity, particularly for strong TCs (category 4 and 5 based on Saffir-Simpson scale^8^), have attracted significant scientific attention^2,3^. However, due to both incomplete understanding of physical mechanisms and observational limitations, this issue has remained unresolved for decades^4,5^, with oceanic influences being a focus of research^6,7^.

Ocean serves as the primary energy source for TC development, with sea surface temperature (SST) largely dominating the enthalpy flux supplied to the atmosphere. Based on the balance between the energy supply and dissipation within the boundary layer, the Potential Intensity (PI) theory^9,10^ establishes a direct relationship between SST and the maximum attainable TC intensity, predicting an increase of TCs’ lifetime maximum intensity (LMI) with SST in a warming climate^11^. However, application of this theory is hindered by great knowledge gap between the climatological SST (or the pre-storm SST) and the under-storm SST, which could largely depart from the former due to the SST cooling effect^12,13^.

The SST cooling primarily results from TC-driven turbulent mixing and upwelling processes^13,14^. The initial manifestations of this phenomenon precede the TC arrival, and persist over an extended period following a TC’s passage, spanning a few weeks^15^. This cooling effect is jointly determined by storm characteristics including intensity, size and translation speed^14,16,17^ as well as the upper ocean thermal structure. Observational evidence indicates that intense, slow-moving TCs can produce 4 °C–5 °C SST cooling^18^ along the track and beneath the TCs’ core, substantially reducing enthalpy flux out of the ocean and inhibiting further TC intensification^19^. Numerical simulations validate this finding, showing that cold wake effects can reduce TC intensity by more than 50%^13^.

By incorporating the thermodynamic environment and the cooling effect of subsurface waters on SST, the improved ocean-coupled SST yields more accurate TC intensity forecasts^20,21^. Foundational studies have further established that the pre-storm upper ocean heat content is a more reliable predictor of TC activity than the pre-storm SST alone^20–23^. However, these works primarily address the overall TC activity, using mean or integrated metrics, such as mean intensification rate^23^, power dissipation index (PDI)^7,22^, or accumulated cyclone energy (ACE)^24^. The problem of to what extent the most intense TCs are affected by the pre-storm subsurface ocean conditions, and what these effects are at large spatial and long temporal scales remain unresolved. Notably, modulated by subsurface temperature variability, the TC intensity changes may diverge from global warming signals but rather exhibit more regional subsurface characteristics. This is expected to be particularly possible for strong TCs, whose vertical mixing can penetrate 100–200 m depth^18,25^, making subsurface thermal structure exert an upper limit on the TC intensity by substantially reducing SST. The issue of how subsurface conditions affect strong TCs, as well as the related variability, has received much less attention, due in part to the scarcity of long-term subsurface observations. Much of the existing research is limited to case studies^26,27^.

Here, we focus on the annual maximum LMI, which is defined as the highest LMI value observed among all TCs in a given year. This metric is selected as an indicator of the upper limit of the subsurface ocean’s capacity in sustaining TC intensification when atmospheric conditions are relatively favorable. By contrast, while mean and integrated metrics are valuable for capturing the full spectrum of TC activity, they are influenced by various factors including storm frequency, duration, and track. These factors are more dominated by large-scale atmospheric conditions^28–30^. Additionally, these quantities include both strong and weak storms. Weak winds generally induce shallow mixing, which are more susceptible to ocean surface conditions. This complicates the task of isolating the distinct role of subsurface ocean temperatures in driving TC intensification.

As one of the world’s most TC-active basins, the western North Pacific not only has the largest number of TCs but also holds about one third of strong TCs^3^, making the ocean-TC interaction research in this region scientifically indispensable for understanding global TC behavior. Recent decades have witnessed a rapid accumulation of ocean observations, including Argo floats that offer vertical profiles of temperature/salinity (T/S) at high temporal (10-day) resolution^31^, the EN4 dataset that supplies gridded, quality-controlled monthly T/S in the ocean interior^32^, and the sustained biannual hydrological measurements along the 137°E meridian from equator through the TC active region for more than 50 years^33^. These datasets, combined with 45-year multiple atmospheric variables datasets and the TC best-track records enable a systematic investigation of the relationship between subsurface thermal conditions and the intensity of the strongest TCs in the western North Pacific.

The present study investigates how the strongest TCs evolve under a changing climate over the past four decades (1975–2020), with a focus on the role of the subsurface ocean in modulating the annual maximum LMI. We reveal the phenomenon and the underlying mechanism by which a particular subsurface water mass dominants the observed V-shaped, multi-decadal variation pattern of the annual maximum LMI. This water mass can be traced through a trans-basin, quasi-conservative, advective path to the subduction region in the eastern North Pacific. Therefore, changes in the annual maximum LMI of TCs in our study region originate from earlier variations in the properties of the subducted water mass and can be predicted by the intensity of the North Pacific High (NPH) a few years in advance.

## Results

### The correlation between annual maximum LMI and subsurface water temperature

**Fig. 1:**
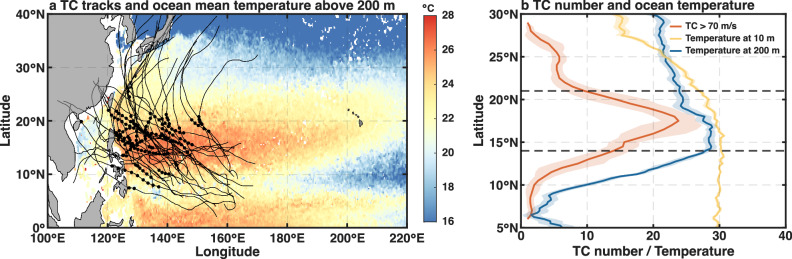
Super tropical cyclones (TCs) and ocean water temperature. **a** Tracks of super TCs since year 2010, superimposed on the mean temperature for the upper 200 m based on Argo data (shade). Black lines represent TC tracks with lifetime maximum intensity (LMI) exceeding 70 m s^−1^. Black dots overlaid on the tracks indicate locations where the wind speed exceeds 70 m s^−1^. **b** Meridional distribution of the accumulated occurrence (red line, in 6-hour intervals) of super TCs for 1975–2020, alongside ocean surface (yellow line) and subsurface (blue line) temperature. The shading denotes one standard deviation. The temperature values presented are normalized (Methods). TC data used in (**a**) and (**b**) are from IBTrACS-USA.

Though tracks of TCs are widely distributed in the western North Pacific, the peak winds of the extreme storms are much more closely clustered in a small region (120°–150°E, 14°–21°N, hereafter the TC region), where a significant number of super TCs (defined as category 5 with LMI > 70 m s^−1^) was found to occur according to the USA agencies’ TC best-track database collected in the International Best Track Archive for Climate Stewardship (IBTrACS)^34^. The higher occurrence of the most intense TCs in this latitudinal band may partly reflect the time required for TCs to reach their maximum intensity after forming farther south and tracking a long distance westward or northwestward^35,36^. Beyond this dynamical preconditioning, however, a notable thermodynamic correspondence is observed. This key region for peak winds coincides with where climatologically the upper ocean (0–200 m) is overwhelmingly warm, even warmer than the tropical area to the south (Fig. 1a). In addition, the meridional distribution of these super TCs during 1975–2020 aligns more closely with the subsurface temperature at 200 m depth than with the surface temperature (Fig. 1b). This counter-intuitive feature raises the question of which pre-storm temperature, the one at the surface or the one in the interior, is more decisive for LMI of strong TCs.

To find the answer, we calculated simultaneous correlations between the annual maximum LMI in the TC region and the annual mean temperature as a function of density through the local water column. The calculation is for the period 1975–2020 with the TC data from 4 agencies, which are USA agencies (USA), the Tokyo-Typhoon Center of the Japan Meteorological Agency (JMA), the Hong Kong Observatory (HKO), the China Meteorological Administration (CMA), as archived in IBTrACS. Since different agencies use different averaging periods for TC intensity measurements, TC intensities are first converted to 1-minute averaged maximum sustained wind speeds^37,38^ (Methods).

**Fig. 2:**
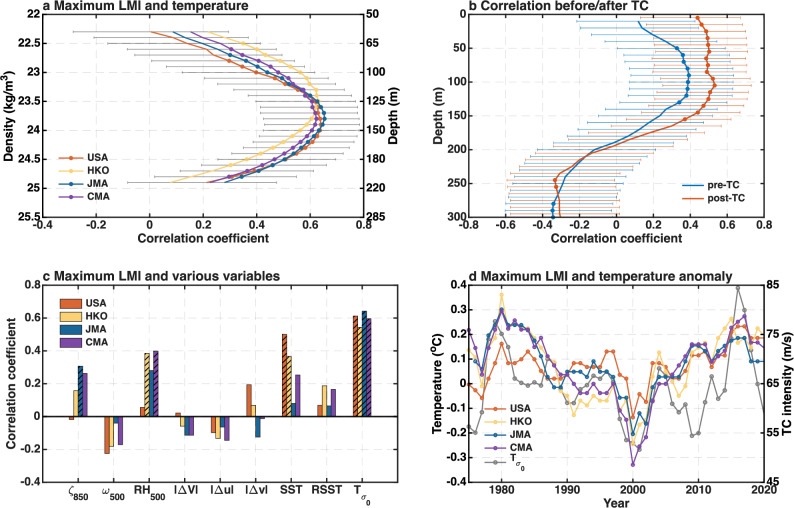
Correlation between tropical cyclone (TC) intensity and various oceanic and atmospheric variables. **a** Simultaneous correlations between the annual maximum lifetime maximum intensity (LMI) and the temperature (averaged between 14°–21°N) through the water column along the 137°E meridian. Color dots show results statistically significant (*p* < 0.05), and the gray bars show the 95% confidence intervals of analysis based on IBTrACS-USA data. **b** Correlations between TC intensity (LMI > 70 m s^−1^) and the local temperature at different depths before (blue) and after (red) TC passage based on Argo data. Color dots show results statistically significant (*p* < 0.05); blue and red bars show the 95% credible intervals. **c** Simultaneous correlation between the annual maximum LMI and various atmospheric and oceanic variables. ${\zeta }_{850}$, vorticity at 850 hPa; ${\omega }_{500}$, omega (${dp}/{dt}$) at 500 hPa, $p$ is the pressure; ${{RH}}_{500}$, relative humidity at 500 hPa; $\left|\triangle {{{\bf{V}}}}\right|$, full shear of 200–850 hPa; $\left|\triangle u\right|$, shear of zonal wind of 200–850 hPa; $\left|\triangle v\right|$, shear of meridional wind of 200–850 hPa; SST, sea surface temperature over the TC region; Relative SST (RSST), SST over the TC region relative to North Pacific tropical mean SST; ${T}_{{{{{\rm{\sigma }}}}}_{0}}$, ocean temperature along the isopycnal 24 kg m^−3^. Data used are from summer months (July–September). Black slashes indicate correlation coefficients that are statistically significant (*p* < 0.05). **d** Time series of the maximum LMI and temperature anomaly along the isopycnal 24 kg m^−3^. TC data used in (**b**) are from IBTrACS-USA. A 3-year running mean smoothing was applied to all time series.

The correlation curves of all 4 TC datasets exhibit a high degree of alignment, indicating a distinctive pattern. The pattern is characterized by a pronounced subsurface peak (R > 0.6, *p* < 0.05) around the potential density ${\sigma }_{0}$ = 24 kg m^−3^, which is located about 150 m beneath the mixed layer. From there, the correlation shows a rapid decline in both upward and downward directions (Fig. 2a). The robustness of this result is further assessed by two alternative methods (Methods), both yielding consistent patterns with that in Fig. 2a (Supplementary Fig. 1).

Hydrographic measurements were taken only twice a year along 137°E, once in winter and once in summer. To overcome the limited temporal resolution, the correlations between super TCs’ LMI and the pre- as well as post-storm temperature profiles were analyzed using Argo floats data which are available at higher temporal resolutions (Methods). A clear distinction is evident between the two correlation curves at different times. For the pre-storm temperature, the strongest correlation occurs at subsurface layers, consistent with findings from the 137°E data. This pattern changes markedly after TC passage, as mixing and upwelling homogenize the upper ocean temperature profile, leading to uniform correlation with LMI (Fig. 2b). This finding lends further evidence for the close relation between the pre-storm subsurface temperature and the super TCs’ LMI.

To further elucidate the influence of subsurface temperature, a comprehensive analysis of multiple atmospheric and oceanic factors considered relevant for TC intensity was conducted. While atmospheric variables have been demonstrated to play a role in TC’s duration or short-term intensity fluctuations^29,30^, they demonstrate a limited linkage to the variability of annual maximum LMI. Local SST relative to North Pacific tropical mean SST also demonstrates a weak relation to the strongest TCs (Fig. 2c). In contrast, the oceanic subsurface temperatures, particularly the temperature on the ${\sigma }_{0}$ isopycnal, demonstrate high consistency with annual maximum LMI, exhibiting quasi-decadal variabilities superposed on a multi-decadal V-shaped variation, with the minimum around 2000, but no appreciable long-term trend over the past four decades (Fig. 2d).

Given this exclusively high correlation, it is reasonable to hypothesize that subsurface temperature in the study region has a strong impact on TCs’ peak intensity. In order to verify the hypothesis, it is first necessary to eliminate one possibility that the observed variation of the annual maximum LMI is largely determined by TC activity before entering the study area. Towards this goal, we investigated the intensity increment the TC attains in the study region, defined as the difference between the maximum LMI and the TC’s final intensity outside the region (Methods). Similar results to Fig. 2 were obtained, showing high correlation between the intensity increment and the subsurface temperature (Supplementary Fig. 2). Hence, a strong linkage between the annual maximum LMI and the subsurface temperature is established.

### The transport path of the subsurface water mass

**Fig. 3:**
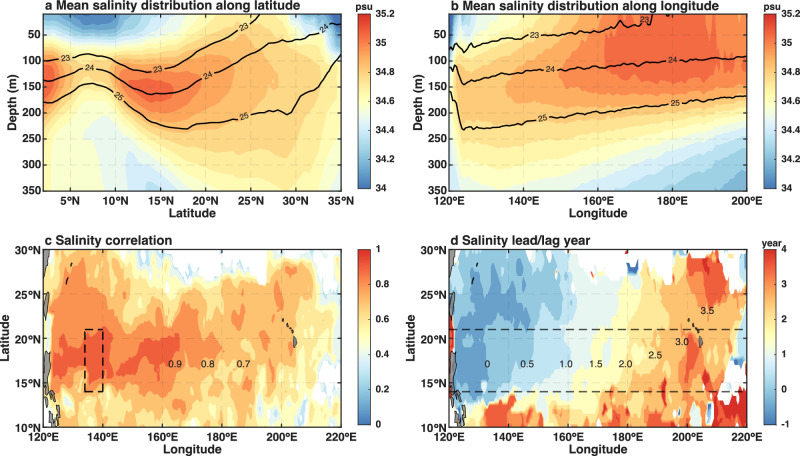
The transport path of the subsurface water based on Argo data. **a** Meridional distribution of subsurface mean salinity averaged over 120°–150°E (shade). **b** Zonal distribution of subsurface mean salinity averaged over 14°–28°N (shade). Black lines in (**a**) and (**b**) indicate isopycnals 23 kg m^−3^, 24 kg m^−3^ and 25 kg m^−3^. **c** Strongest correlations (above 95% confidence level) between the salinity within the box (134°–140°E, 14°–21°N, denoted by a black dashed box) and that of all other regions along the isopycnal 24 kg m^−3^. **d** Lead/lag periods corresponding to the strongest correlations shown in (**c**). Black dashed lines denote latitudes 14°N and 21°N, respectively.

As demonstrated previously, the pre-storm temperature most correlated with the annual maximum LMI in the TC region is that around the 24 kg m^−3^ isopycnal. Clearly seen from a meridional section of salinity through the region, this isopycnal is at the core of a warm and saline water mass, with maximum salinity exceeding 35 psu below the mixed layer and above 200 m depth (Fig. 3a). Since the salinity maximum appears at the subsurface, the water mass is not generated from the local air-sea interaction process but from somewhere else.

In the climatological mean state, the density surfaces slope upward from low latitudes and intersect the mixed layer in subtropical regions (Fig. 3a, b). The zonal distribution of salinity averaged over 14°–21°N shows that the 24–25 kg m^−3^ isopycnals outcrop east of 180°E, where a large volume of saline waters are ventilated (Supplementary Fig. 3). These features suggest that the saline water mass is likely generated in the eastern North Pacific’s ventilation zones, and subsequently transported to the TC region along a subsurface path. To identify this path, we calculated lead-lag correlations of salinity along the isopycnal 24 kg m^−3^ between an area near the western boundary (134°–140°E,14°–21°N, denoted by a rectangular box in Fig. 3c) and other regions based on Argo data (Methods). A quasi-conservative transfer path stands out, characterized by strong correlations and the lead time declining continuously and progressively westward from the ventilation region (Fig. 3c, d). The maximum lead time is about 4 years, indicating the transfer time of the water mass to the western boundary.

Around 14°–21°N, the baroclinic instability caused by Subtropical Countercurrent (STCC) create large amounts of mesoscale eddies^39,40^, propagating westward due to the planetary $\beta$ effect. Existing studies show that mesoscale eddies play a key role in the transport of mode waters across the Pacific basin^41,42^. Our analysis confirms that eddies in this latitudinal band, independent of their polarities, can trap and carry the saline water mass continuously westward over a long distance (Supplementary Fig. 4).

### Water mass formation and NPH

**Fig. 4:**
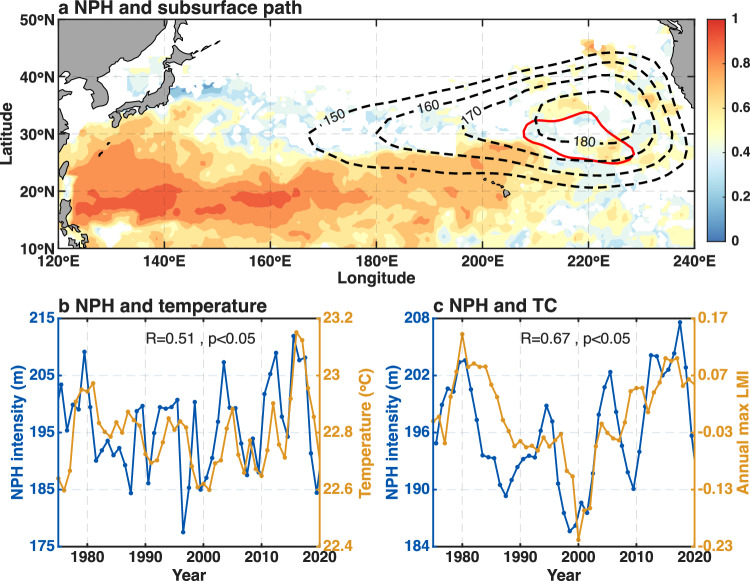
The relation among the North Pacific High (NPH), the annual maximum lifetime maximum intensity (LMI) and the subsurface water temperature. **a** Spatial distribution of the NPH and the subsurface water mass transport path. The shading denotes the strongest correlations (above 95% confidence level) between the temperature within the area (134°–140°E, 14°–21°N, as the same showed in Fig. 3c and that of all other regions along the isopycnal 24 kg m^−3^. The NPH intensity is calculated as the average of the ten largest values of geopotential height at 1000 dbar. The dashed contours represent the climatology NPH distribution, with level of 150 m, 160 m, 170 m and 180 m. The red contour marks the area in the eastern North Pacific where the mixed layer depth in February exceeds 120 m based on WOA2018 dataset. **b** Time series of NPH intensity (blue) and subsurface temperature along the isopycnal 24 kg m^−3^ (orange) based on 137°E dataset during 1975–2020. The line of NPH intensity was shifted to the right for 4.5 years. A 1-year smoothing was applied to both time series. **c** Time series of NPH intensity (blue) and annual maximum LMI (orange). The annual maximum LMI time series is averaged by four agencies (USA, HKO, CMA and JMA) and is normalized. The line of NPH intensity was shifted to the right for 5.5 years. A 3-year smoothing was applied to both time series.

A similar correlation analysis was conducted for temperature along the same isopycnal (Fig. 4a), and the resulting patterns are consistent with those for salinity (Fig. 3c). This coherence is expected, due to the along-isopycnal compensation between salinity and temperature as well as the quasi-conservation of both properties following the westward propagation of the water mass.

The result clearly reveals that the subsurface water mass, whose temperature was found closely related to variation of the annual maximum LMI, originates from the subtropical outcrop region in the eastern North Pacific (Fig. 4a). This region, which also coincides with the area where the climatological mixed layer depth (MLD) in late winter (February) exceeds 120 m (red contour in Fig. 4a), is characterized by deep mixing and is a known formation zone of the North Pacific Eastern Subtropical Mode Water (NPESTMW)^43,44^. Moreover, as we turn our attention to the atmosphere, the formation site is under the center of the North Pacific High (NPH)^45^, a large-scale pressure system in the eastern Pacific Ocean with the climatological mean position around (220°E, 35°N). This convergence implies a close dynamical linkage with surface oceanic and atmospheric conditions in the formation and subduction of the NPESTMW. More importantly, since the temperature and salinity of the water mass are largely preserved during its westward transit, their interannual-to-decadal variabilities often reflect changing air-sea fluxes as well as surface conditions in formation regions^46–48^.

We systematically examine the lead-lag relationships of the annual maximum LMI with key surface variables influencing formation of NPESTMW (Supplementary Fig. 5), as well as the relationships between the temperature along the isopycnal 24 kg m^−3^ in the TC region and these variables (Supplementary Fig. 6). The examined variables include wintertime wind stress, MLD, and buoyancy flux averaged over the water mass formation region (200°–240°E, 20°–35°N)^44^, as well as two large-scale climate indices, the NPH intensity and the Pacific Decadal Oscillation (PDO) index^49^.

Among these factors, the NPH shows the strongest correlation with both the annual maximum LMI and the subsurface temperature. A lead of 4.5-year is observed between the NPH intensity and the core temperature of the subsurface water mass in the TC region under 1-year smoothing (Fig. 4b). This lead is a slightly larger than, but close to the transit time of the water mass, as demonstrated by Fig. 3d. The difference between the two can be explained by the lag of a few months between water mass formation and the initiation of its westward transport^44,50^. When being compared directly with TCs’ annual maximum LMI, the NPH intensity reveals a strong correlation (Fig. 4c). The lead time increases to over five years, due to the artificial effect of the 3-year smoothing procedure.

Additionally, we constructed composite analyses of sea level pressure (SLP), wind stress, and MLD over the North Pacific Subtropical High (NPSH) region for years with strong and weak TC events (Methods). Composite analyses were performed for the five years preceding each type of event to examine precursory signals in the ocean-atmosphere system (Supplementary Fig. 7). The results indicate that NPH intensity anomalies are strongly correlated to regional wind stress and MLD, with distinctly opposing patterns prior to strong versus weak TC events. Prior to strong (weak) TC events, the NPH is intensified (weakened), accompanied by an enhanced (reduced) high-pressure system over the eastern North Pacific and increased (diminished) wind stress around its periphery. These conditions may enhance (reduce) oceanic heat loss, deepen (shallow) the mixed layer in water mass formation regions, thereby influencing the formation and physical characteristics of water masses.

In summary, the warm and saline water mass, which is closely related to the variation of the annual maximum LMI, is formed under the NPH, effectively preserves atmospheric signals and is subsequently transported quasi-conservatively through subsurface pathways. Over the past four decades, changes of NPH consist of quasi-decadal oscillations superimposed on a striking multi-decadal V-shaped structure. The observed V-shaped variation of the annual maximum LMI is therefore likely a consequence of the earlier changes of factors, such as NPH in the eastern North Pacific.

### Mechanism of subsurface water dominating evolution of under-storm SST

At first glance, the observation that the annual maximum LMI is influenced more by the pre-storm subsurface temperature than by the pre-storm SST is intriguing. A key factor underlying this phenomenon is the considerable depth to which TCs’ effects can extend. Based on pre- and post-storm observations of upper ocean T/S structure, we estimated the depth influenced by strong TCs (category 4 and 5) in the study region through two different approaches (Methods). The first estimated was the well-mixed depth, where the upper ocean becomes fully mixed under a storm. This depth was found to generally exceed 100 m, reaching approximately 150 m, which corresponds to the central depth of the subsurface water mass. The second estimated was the TC-affected depth, a deeper layer where water parcels undergo partial mixing and modification without full homogenization^51,52^. This depth averaged above 150 m and could extend as deep as 230 m (Supplementary Fig. 8), suggesting an upper limit of the depth influenced by TCs. Although the well-mixed depth and TC-affected depth are defined differently, both estimates indicate that effects of strong TCs extend well beyond the surface mixed layer, entraining much of the particular subsurface water mass and causing it to mix with waters above.

Although modeling studies have demonstrated that TCs can effectively bring information of subsurface thermal structures to the surface^28^, we carried out experiments using the Massachusetts Institute of Technology general circulation model (MITgcm)^53^ (Methods) to conduct a thorough investigation of the related process in the study region. Initializing the model with observed T/S profiles along the 137°E section, we imposed an identical TC forcing with high LMI to quantify the evolution of temperature through the water column during TC passage. It is well recognized that both turbulent mixing and upwelling can induce SST cooling, though the latter has received less attention in previous research. To disentangle the different roles of these two processes, we isolated them by selectively enabling or disabling horizontal advection in the mixed layer, as well as adopting a relatively low translation speed of TC (Methods).

**Fig. 5:**
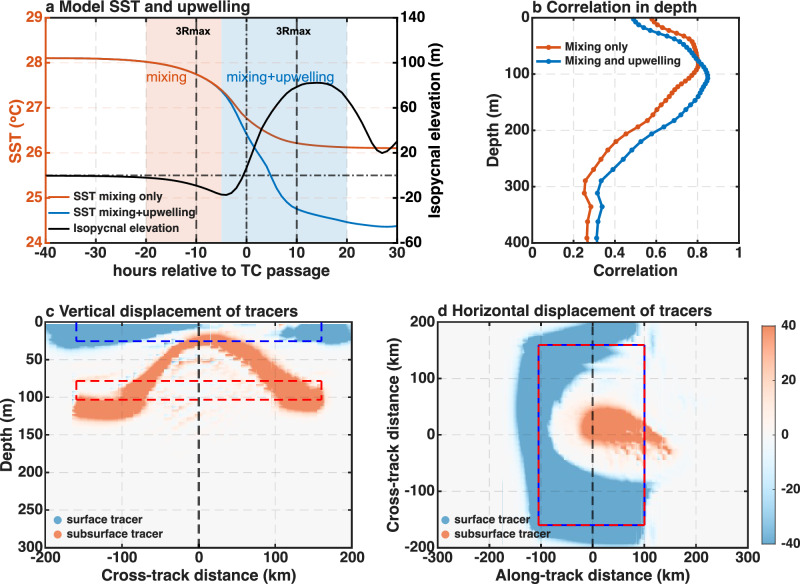
Modeled response of ocean temperature. **a** Time evolution of sea surface temperature (SST) from the model in the mixing only case (red) and the mixing+upwelling case (blue), along with the isopycnal elevation in the mixing+upwelling case (black). **b** Correlation between the modeled under-storm SST and the pre-storm temperature at different depths in the mixing-only case (red line) and the mixing+upwelling case (blue line; Methods). **c** Vertical displacement of surface and subsurface water 6 h after the TC passage (Methods). Two tracers, denoted by two colors, are initialized in a surface region (blue dashed box) and a subsurface (red dashed box) region. As upwelling occurs, the surface tracer is pushed away while the subsurface tracer is lifted to the surface (shade). **d** the same as (**c**) but for horizontal displacement of the tracer at 20 m depth.

Our simulation shows that the mixing process begins long (>10 hours) before the TC’s arrival^14,28,54^. By the time the TC reaches the area, the under-storm SST has become a blended temperature that reflects both surface and subsurface thermal properties (Fig. 5a). The under-storm SST is found to correlate strongly with the pre-storm subsurface temperature, particularly at the depth of about 80 m (red line in Fig. 5b).

When upwelling is allowed, subsurface isopycnals are elevated as much as 60 m under strong TCs (black solid line in Fig. 5a), enhancing the entrainment of deeper waters into the mixed layer, and pushing near-surface waters away from the TC’s track (Fig. 5c, d). While vertical mixing remains a key driver of SST changes across all TC translation speeds, upwelling becomes particularly important for slow‑moving storms^14^. The combined effects of mixing and upwelling lead to significantly greater SST cooling, reaching nearly double the cooling observed in the mixing-only scenario (Fig. 5a). This effect could substantially increase the likelihood of reaching the temperature threshold that prevents further TC intensification, highlighting the critical role of subsurface temperature in determining TC strength. In the presence of both mixing and upwelling, the correlation between the under-storm SST and the pre-storm subsurface temperature follows a similar pattern to the mixing-only scenario, but with the peak value shifted to an even deeper depth (blue line in Fig. 5b).

Although model simulations have confirmed the main observed feature, the underlying physical mechanism remains largely unclear. While it is intuitive that a relatively warm subsurface can support strong TCs, the process by which the correlation peaks in the subsurface is more complex. Consider the extreme case where the subsurface temperature is excessively high but constant. In this scenario, the well-mixed temperature would undoubtedly favor TC intensification due to the subsurface contribution, yet its variation would be correlated only with the pre-storm temperature at the surface. This suggests that a highly variable subsurface is likely a necessary condition for the formation of the subsurface correlation peak.

**Fig. 6:**
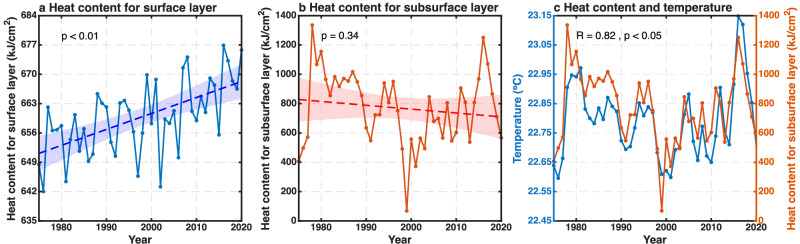
Heat content of the surface and subsurface layers. **a** Time series of surface layer heat content. **b** Time series of subsurface layer heat content. **c** Comparison of the temperature along the isopycnal 24 kg m^−3^ and the subsurface layer heat content. Dashed lines in (**a**) and (**b**) show the linear regression with p-value denotes on each panel. The shaded area indicates the 95% confidence interval.

Based on a linear trend analysis, we identified two thermally independent layers within the upper ocean of the study area (Supplementary Fig. 9). The surface mixed layer exhibits a strong linear warming trend (Fig. 6a), whereas the subsurface layer containing the warm and saline water mass shows a V-shaped, multi-decadal variation pattern rather than a significant long-term trend since 1975 (Fig. 6b). More importantly, the heat content of the subsurface layer, which closely aligns with the core temperature of the water mass (Fig. 6c), is much more variable than that of the surface mixed layer.

According to the results of a theoretical model (Methods), the correlations between the under-storm SST and the pre-storm temperature of the surface and subsurface layers have the relation of $\frac{R\left({T}_{{{{\rm{mix}}}}},{T}_{1}\right)}{R\left({T}_{{{{\rm{mix}}}}},{T}_{2}\right)}\approx \frac{\sigma {C}_{1}}{\sigma {C}_{2}}$, where ${T}_{{{{\rm{mix}}}}}$ is the well-mixed temperature during a TC’s passage; ${T}_{1}$, ${T}_{2}$ represent temperature of the surface and subsurface layer, respectively; ${C}_{1}$ and ${C}_{2}$ are the heat content of the surface and subsurface layers. $R$ denotes the correlation coefficient between two variables, and $\sigma$ represents the standard deviation. Given that the well-mixed layer for strong TCs is about 150 m in the study area, the ratio between the variance of the two layers is over 20, indicating a much greater correlation between the under-storm SST $({T}_{{{{\rm{mix}}}}})$ and the subsurface temperature $({T}_{2})$ than that related to the surface temperature (${T}_{1}$). This is likely the reason for the subsurface peak observed in the correlation profile.

Therefore, effects of strong TCs in the study region extend far beyond the surface mixed layer, causing much of the subsurface warm and saline water mass to mix with surface waters. Due to the greater variability in heat content of the subsurface layer, the under-storm SST is highly correlated with the thermal conditions of the subsurface layer rather than the surface layer. Furthermore, when consider the presence of TC-induced upwelling as indicated by model simulations, more subsurface waters participate in the mixing process as near surface waters are pushed away from the TC track by flow divergence, thus amplifying the effects of subsurface thermal structure on under-storm SST evolution (Fig. 5).

## Discussion

This study focuses on a critical region in the western North Pacific (120°–150°E, 14°–21°N), known for its high frequency and intensity of strong TCs. This region is underlain by a warm, saline subsurface water mass, which provides a favorable thermal background for development of intense TCs. More importantly, we identified a notable and unexpected multi-decadal variation in the behavior of the annual maximum LMI, which closely mirrors changes in this particular subsurface water mass in the past four decades with little sign for long-term trend. This finding is somewhat surprising because it challenges the expectation that global warming will result in intensification of TCs^28,55^. Instead, the observed V-shaped pattern suggests that the annual maximum LMI is more closely tied to a particular subsurface layer than to the pre-storm SST or to the pre-storm upper ocean overall heat content^20,22^. The particular water mass originally forms and is subducted in the eastern North Pacific beneath the NPH. It is subsequently carried, mainly by oceanic mesoscale eddies, quasi-conservatively along a subsurface path to the TC region. The entire process, from formation to arrival, takes about 4.5 years. The consistency relationship between the annual maximum LMI and the NPH intensity makes it possible to predict the TCs’ peak intensity a few years in advance. This finding can help resolve the reliable prediction issue of intense TCs that has haunted the community for a long time. Based on the multi-decadal V-shaped variation pattern of NPH, it is unlikely that the annual maximum LMI in the western North Pacific will increase in the next few decades, despite the ongoing global warming trend. While the relationship between global warming and the annual maximum LMI of TCs in the study area is weak, this does not imply that global warming has no impact on TCs. We suggest that weak TCs, which typically induce shallower mixing^56^, are more directly influenced by surface ocean conditions. As a result, mean or integrated metrics reflecting both strong and weak TC activity show a rising trend, which aligns with the broader effects of global warming^23,57^.

Our targeted analysis of the most intense TCs allows us to isolate and highlight as much as possible the subsurface conditions that may otherwise be obscured in broader studies that aggregate all TC events. To test the robustness of our results, we also performed the analysis using different quantiles of TC intensity (Supplementary Fig. 10). The correlations using the top 25% LMI are nearly identical to those using the maximum LMI, confirming that the use of the maximum LMI in our analysis is robust. By contrast, using the mean LMI of all TCs yields weaker and less consistent results, likely due to the inclusion of weaker storms with reduced TC-induced mixing. Although the annual maximum LMI metric may not fully capture overall TC activity, it serves as a more precise indicator of the ocean’s maximum capacity to sustain the most intense TC events. This approach provides complementary insights into how subsurface ocean conditions influence the most powerful storms, a topic that has been less explored in existing research.

A theoretical model is proposed to explain the subsurface peak in the correlation between the annual maximum LMI and the pre-storm temperature profile. The mechanism suggests that this subsurface peak is driven by three factors: the great well-mixed depth of strong TCs, the independent variations of the surface mixed layer and the subsurface water mass, and the exceptionally high variability in the properties of the specific subsurface water mass.

Building on these findings, we now clarify the fundamental significance of our discovery while emphasizing its regional character. The robust linkage between the subsurface warm water mass and TC maximum intensity is regional. Its occurrence depends on two conditions: the presence of a warm subsurface water mass located at the proper depth within the reach of the TC-induced mixing, and the occurrence of strong TCs in the same region. With both conditions satisfied, the subsurface water can be effectively mixed upward during TCs’ passage, modulating the under-storm SST change. A similar process may also occur in other basins with analogous conditions, such as the western North Atlantic, which also features both intense TCs^58^ and warm subsurface water subducted from the outcrop region in the northeast^59,60^. In contrast, such phenomena do not seem possible in the eastern sides of basins where the subsurface warm water mass is misaligned with occurrence of strong TCs^24^. Nevertheless, while the phenomenon exhibits regional characteristics, the underlying mechanism by which subsurface ocean variability dominates the most intense TCs is fundamental and universal. The interannual variability of the under-storm SST is not governed by the layer with the highest temperature, but rather by the layer exhibiting the greatest variability in heat content. Consequently, the interannual variation in local maximum TC intensity can be determined by the character of a particular subsurface layer subducted and transported remotely from higher latitudes. The TC’s intensity is therefore affected by climatic variability at middle and high latitudes a few years in advance, representing a potential pathway that links TC’s activity with the dynamics at the global scale.

A question arises as to whether current high-resolution climate models are capable of reproducing the observed linkage between TC intensity and NPH strength. Here we evaluated performance of coupled models using outputs of six high-resolution models from the High Resolution Model Intercomparison Project (HighResMIP) of CMIP6 (Methods), which has garnered considerable attention for its capability in simulating the observed frequency and geographical distribution of TC genesis with reasonable accuracy^61,62^. We found that these models generally lack the ability to capture the observed linkage between early sea surface signals in the eastern North Pacific and the subsequent TC maximum intensity in the western North Pacific (Supplementary Figs.11–13). This remarkable discrepancy arises primarily from the substantial underestimation of TC intensity (Supplementary Fig. 12) by models^63^, which induces weak and shallow mixing that leaves the subsurface water largely intact. In addition, though similar water mass was found to be subducted in the eastern North Pacific, its properties were poorly conserved, limiting the persistence of the subducted signal along the transfer path (Supplementary Fig. 13). These findings highlight important limitations in current model simulations of ocean-atmosphere coupling and TC intensification, providing valuable insights for future model development.

Going forward, further investigation is warranted to determine whether a similar mechanism operates in other ocean basins. Achieving this requires not only the establishment of a comprehensive subsurface observational network, but also the development of air-sea coupled, high-resolution models with improved sub-grid schemes.

## Methods

### Correlation between TC intensity and potentially important atmospheric and oceanic factors

The TC data over the western North Pacific are from the International Best Track Archive for Climate Stewardship (IBTrACS), which provide TC tracks and intensities at 3-hour intervals^34^. We utilize TC records from four agencies of USA, JMA, HKO and CMA. Time period in use is 1975-2020. To account for measurement discrepancies among agencies, the TC intensity values are first converted to 1-minute averaged maximum sustained wind speeds^37,38^. Subsequently, each agency’s intensity is normalized by dividing its respective mean value and then multiplying the total mean value of the 4 agencies. We define the TC region as 120°–150°E, 14°–21°N, where most super TCs occur (Fig. 1). For each year, we calculate the LMI of all TCs occurring within this region and use the annual maximum LMI to reflect the upper limit of the subsurface ocean’s capacity to sustain TC intensification.

The measurements along the 137°E section, obtained by shipboard observations of the JMA and including temperature and salinity profiles from 1975 to 2020 (ref. ^33^), are used to calculate temperature correlation with the maximum LMI. The data are averaged between 14°–21°N to represent the hydrological characteristics in the TC region.

Atmospheric variables including zonal and meridional winds ($u,v$), relative humidity (RH) and omega ($\omega$) are from National Centers for Environmental Prediction (NCEP)/ National Center for Atmospheric Research (NCAR) Reanalysis1^64^ with 2.5$^\circ \times$2.5° spatial resolution and daily temporal resolution. All atmospheric variables are from time period 1975–2020. SSTs are from the Optimum-Interpolation Sea Surface Temperature (OISST) of National Oceanic and Atmospheric Administration (NOAA)^65^. It provides 0.25$^\circ \times$0.25° spatial resolution and daily temporal resolution, with available time period 1982–2020. Given that TC activity in the western North Pacific peaks in summer months, here we use the data during July–September to represent typical atmospheric and oceanic conditions during the TC season and examine their correlation with the annual maximum LMI.

### Robustness of the annual maximum LMI-temperature relationship

To assess the robustness of this annual maximum LMI-temperature relationship, two alternative methods were used. The first examined localized temperature variations using the same 137°E data, but with data extracted within a 4° latitude window centered on each locale of the TCs’ annual maximum LMI, instead of averaged within 14°–21°N band. The second method utilized EN4 data to derive area-averaged temperature fields across the entire study region (120°–150°E, 14°–21°N), capturing broader-scale thermal characteristics (Supplementary Fig. 1).

### Correlation between TC intensity and temperatures from Argo profiles

To further investigate the ocean thermal response before and after TC passage, we examine the relation between TC intensity and temperature at different depths before and after TC passage (Fig. 2b). This analysis focuses on super TCs whose LMI exceeded 70 m s^−1^ based on IBTrACS-USA data, covering the period from 2004 to 2020. We identify locations of super TC intensities and select Argo profiles from 1–3 weeks before TC arrival within a 5°$\times$5° box as pre-TC profiles. Post-TC profiles are collected within 1 week after TC arrival.

### TC Intensity increment

For each year, we first identify the maximum LMI of TCs. By examining the same TC prior to its entry into the TC region, we calculate the intensity increment as the difference between its maximum LMI and its final intensity outside the region. This increment can be regarded as a variable influenced by regional factors.

### The lead-lag correlations analysis

To identify the origin of subsurface water mass in the TC region, we use salinity/temperature as a tracer (Fig. 3c, d, Fig. 4a). The reference salinity/temperature is defined as the spatial average within the region 134°–140°E, 14°–21°N along the isopycnal 24 kg m^−3^. We perform correlation analysis between this reference time series and salinity/temperature fields (binned into 5° × 5° subregions) throughout the North Pacific along the same isopycnal based on Argo float data for time period 2004–2020. By examining the lead-lag correlations, we determine the optimal time period corresponding to the strongest correlation, thereby indicating whether salinity/temperature anomalies at each location led or lagged relative to the reference region.

### Mesoscale eddies carrying water mass westward

A global Mesoscale Eddy Trajectory Atlas (MET3.2 exp DT two-satellites)^66^ was employed to assess the typical sea surface height anomaly (SSHA) values of mesoscale eddies. It is composed of eddy identification and trajectories produced with altimetric maps. The eddy energy flux is calculated as the eddy propagation speed multiplied by the eddy potential energy calculated by META data (Supplementary Fig. 4a).

The time-longitude analysis of salinity is performed within the region 120°–240°E, averaged over 14°–21°N. Monthly salinity anomalies are derived from Argo data for the period 2004–2020 (Supplementary Fig. 4c).

To analyze the three-dimensional structure of eddies, we utilize Argo profile data within eddies for the region 120°–150°E, 10°–25°N. Mesoscale eddy positions within the composite domain are identified using META data, which provide eddy locations and time information. Each Argo profile is matched with the nearest eddy. The Argo profiles should be observed within 3 days before and after the observation time of eddy. This spatiotemporal matching is applied to all available eddies (with radius exceeding 40 km) and Argo floats in this domain. Finally, Argo profiles are reconstructed based on their distance from eddy centers and eddy polarity (Supplementary Figs. 4de).

### Data normalization

Z-score transformation procedure is used for normalizing temperature data in Fig. 1b.

1 $$Z=\frac{T-\bar{T}}{\sigma \left(T\right)},$$

where $T$ is the original temperature series, $\bar{T}$ is its mean, $\sigma (T)$ is its standard deviation, and $Z$ is the normalized data. The Z-score method centers and scales the data to achieve a zero mean and unit standard deviation^67^. In Fig.1b, to focus on the latitudinal distribution pattern rather than absolute temperature values, we first normalize the temperature data and then apply a scaling coefficient to align the range of TC number, enabling their display on a shared x-axis.

### North Pacific High intensity

The NPH intensity is calculated as the average of the ten largest values of the six-month mean geopotential height at 1000 dbar. The geopotential height data are from NCEP/NCAR Reanalysis1^64^ with 2.5$^\circ \times$2.5° spatial resolution and daily temporal resolution. Time period is from 1975 to 2020. The climatological spatial distribution of NPH is obtained by averaging the geopotential height at 1000 dbar over the period from 1975 to 2020.

### Relationship between annual maximum LMI and various variables potentially significant for formation and subduction of the water mass

To investigate the influence of environmental factors on water mass formation. we systematically compared the relationships between annual maximum LMI of TCs and several key surface variables. These include wintertime wind stress, MLD, and buoyancy flux averaged over the water mass formation region (200°–240°E, 20°–35°N), as well as the NPH intensity and the PDO index from 1975 to 2020. The March wind stress is calculated by the bulk formula based on NCEP/NCAR Reanalysis1 monthly dataset with 2.5$^\circ \times$2.5° spatial resolution. The MLD is defined as the depth at which potential density increases by 0.1 kg m^−3^ from 10 m (ref. ^68^) and is averaged over January to March based on EN4 data. The monthly time series of the PDO index is obtained from NCEI and are averaged by a year.

The buoyancy flux $B$ is composed of heat and fresh water contributions^69^ and is given by:

2 $$B=-\frac{\alpha {Q}_{{{{\rm{net}}}}}}{{C}_{{{{\rm{p}}}}}}+{\rho }_{{{{\rm{s}}}}}\beta {S}_{{{{\rm{A}}}}}\left(E-P\right),$$

where $\alpha$ and $\beta$ are the thermal expansion and haline contraction coefficients. ${Q}_{{{{\rm{net}}}}}$ is the net heat flux, and is calculated as the sum of surface sensible heat flux, latent heat flux, net short-wave radiation flux and net long-wave radiation flux. ${C}_{{{{\rm{p}}}}}$ is the specific heat, ${\rho }_{{{{\rm{s}}}}}$ is the sea surface density, ${S}_{{{{\rm{A}}}}}$ is the absolute sea surface salinity, and $E$, $P$ are evaporation and precipitation. The variables used to calculate the buoyancy flux is based on monthly ERA5 dataset with 0.25$^\circ \times$0.25° spatial resolution.

### Numerical simulation of ocean response to TCs

We use the MITgcm to simulate the ocean response to TC. The flat-bottomed ocean with 1000 m depth was configured on an $f$ plane. The KPP turbulence closure scheme^70^ was chosen to parameterize the vertical mixing. The model ocean is 1000 m deep, divided into 55 layers. The thickness of the first 12 layer was set at 5 m, followed by layers with thickness increasing exponentially with depth. The model domain spans 3000 km along-track and 2000 km cross-track, with a uniform horizontal resolution of 10 km. Sponge boundary conditions were used to reduce the effects of wave reflection from the walls.

An axisymmetric azimuthal wind field of TC was adopted. A modified Holland model was used for the model TC forcing. The application of Holland model requires the specification of the maximum sustained wind speeds (${V}_{\max }$), the radius of maximum winds (${R}_{\max }$) and parameter $B$, which controls the radial width of the wind maximum. The formula of $B$ was evaluated by least squares fits of wind profile observed during flight^71,72^.

3 $${V}_{\theta }\left(r\right)={V}_{\max }{{\cdot }}{\left[{\left(\frac{{R}_{\max }}{r}\right)}^{B}{{\cdot }}\exp \left(1-{\left(\frac{{R}_{\max }}{r}\right)}^{B}\right)\right]}^{\frac{1}{2}},$$

4 $$B=0.886+0.0177{V}_{\max }-0.0094\varphi,$$

where ${V}_{\theta }$ is tangential wind speed, $r$ is the distance from the TC center and $\varphi$ is the latitude of the TC.

The wind stresses are then calculated by the bulk formula.

5 $$\tau={\rho }_{{{{\rm{a}}}}}{C}_{{{{\rm{D}}}}}{V}_{\theta }^{2},$$

where ${\rho }_{{{{\rm{a}}}}}=$1.29 kg m^−3^ denotes the air density, ${C}_{{{{\rm{D}}}}}$ is the drag coefficient. Since ${C}_{{{{\rm{D}}}}}$ under high-wind conditions is proposed of level off, we simply apply an upper limit to the formula of ref. ^73^ to form ${C}_{{{{\rm{D}}}}}$ here:

6 $${10}^{3}{C}_{{{{\rm{D}}}}}=\left\{\begin{array}{c}1.2,\,{V}_{\theta } < 11{{{\rm{m}}}}{{{{\rm{s}}}}}^{-1}\\ \min \left[0.49+0.065{V}_{\theta },2.115\right],{V}_{\theta }\ge 11{{{\rm{m}}}}{{{{\rm{s}}}}}^{-1}\end{array}.\right.$$

The surface heat and freshwater fluxes are not considered as model forcing here. Considering the maximum TC LMI for the time period of 1975–2020, averaged as ~76 m s^−1^ (from IBTrACS-USA), we set ${V}_{\max }=$ 80 m s^−1^, ${R}_{\max }=$ 35 km, $\varphi=20^\circ$N, and a constant translation speed as 3 m s^−1^. The typical observed TC translation speed is higher. However, the slower speed (3 m s^−1^) adopted in model simulation was not intended to replicate the most common scenario of the ocean, but rather to create a demonstrative, idealized setup to maximize the difference between upwelling and no-upwelling cases.

Argo profiles are used to validate the simulated upwelling depth, and surface drifters are used to validate the simulated SST cooling (Supplementary Fig. 14). The TC-induced upwelling is conducted by analyzing Argo profile pairs before and after TC passage. Upwelling depth is measured as the vertical shift of the isopycnal below the mixed layer before and after the TC passage. Pre-TC profiles are selected from the 7-day period preceding TC arrival, while post-TC profiles cover the 7-day period following TC arrival. Both Argo/Argos are sampled within 50 km across the TC track, considering only strong TCs (Category 4 and 5). The results show good agreement, indicating that the model captures the ocean response reasonably well.

A total of 46 simulations were conducted, spanning the period 1975–2020. In each experiment, the initial temperature and salinity fields are derived from the annually averaged 137°E measurements between 14°–21°N for the corresponding year (Supplementary Fig. 15). These initial T/S fields were uniformly applied across the entire model domain. The simulations began with a quiescent ocean, which was then forced by the model TC moving steadily over the ocean. Two types of experiments were carried out to analyze the specific contributions of vertical mixing and upwelling to SST with ocean cooling. The mixing-only simulations were configured by disabling horizontal advection in the mixed layer^74^. The full dynamics simulations employed the model’s default settings, allowing both vertical mixing and upwelling to jointly influence SST evolution. The under-storm SST, which is defined as the averaged SST within 2Rmax (radius of maximum wind speed) after the TC passage, is considered to be able to directly influence TC intensity^75,76^.

Secondly, the tracer experiments were designed to track water movement at different depths during TC passage. Initially, the passive tracers were added in the surface (0–25 m), and subsurface (80–105 m) layers with a concentration of 100 respectively (Supplementary Fig. 16). To distinguish the two types of tracers, negative sign is assigned to the surface tracer concentration (Fig. 5c, d). To focus specifically on water advection rather than mixing effects, we maintain the KPP-generated viscosity but disable the diffusivity in the simulations^77^. The TC forcing was the same as described before.

### Estimating the depth of TC influence

We estimated the depth influenced by TCs in our study region using two different approaches. The first method is to estimate the well-mixed depth $H$. It was calculated as the range through which the well-mixing of the pre-storm temperature profile $T(z)$ yields the observed post-storm SST:

7 $${{{{\rm{SST}}}}}_{{{{\rm{post}}}}-{{{\rm{storm}}}}}=\frac{{\int }_{-H}^{0}T\left(z\right){dz}}{H}.$$

The spatial distribution of the observed well-mixed depth (Supplementary Fig. 8a) was computed through the following procedure. The post-storm SST is derived from daily OISST data after TC passage. $T(z)$ is calculated as the climatological summer temperature based on the World Ocean Atlas 2018 (WOA2018) dataset. The TCs are sourced from the IBTrACS-USA dataset and with only those reaching Category 4 or 5 intensity on the Saffir-Simpson scale being selected for analysis. For each TC position, we first extracted the average temperature profile within a surrounding 5°$\times$5° region.

The second method is to estimate the TC-affected depth (Supplementary Fig. 8b). Once water parcels undergo turbulent mixing, both their density and temperature/salinity (T/S) change, even without full well-mixing of the water column. Consequently, the TC-affected depth is typically deeper than the well-mixed depth. Based on this consideration, we compared the T-S profiles at a specific location before and after the passage of a TC. The T-S profiles are based on Argo floats, the pre-storm profiles are selected from the 7-day period preceding TC arrival, and the post-storm profiles are sampled within 0-7 days after TC passage and within 50 km across TC track. We then estimated the TC-affected depth by identifying the maximum depth at which the two curves diverged, indicating perceptible differences in their alignment (Supplementary Figs. 8c–e).

### Mechanism of subsurface water temperature dominating SST evolution during TC-induced mixing and upwelling

In our study region comprises two distinct layers: a warming surface mixed layer and a trendless subsurface layer, divided near 60 m depth (Supplementary Fig. 9). Based on this two-layer structure, we calculate the heat content of each layer (Fig. 6). The correlation between the heat content of these two layers was found to be only −0.06, suggesting their thermal independence. An analytical expression is derived for the correlation between the post-mixing SST and the initial pre-mixing thermal structure of the water column.

The mixed-layer temperature after a TC’s passage (${T}_{{{{\rm{mix}}}}}$) is calculated as:

8 $${T}_{{{{\rm{mix}}}}}=\frac{{\int }_{{-H}_{1}}^{0}T\left(z,t\right){dz}+{\int }_{-({H}_{1}+{H}_{2})}^{{-H}_{1}}T\left(z,t\right){dz}}{{H}_{1}+{H}_{2}},$$

where $T\left(z,t\right)$ is the pre-storm temperature profile, ${H}_{1}$ is the thickness of the surface layer, and ${H}_{1}+{H}_{2}$ is the well-mixed depth. Given this two-layer structure, the terms ${\int }_{{-H}_{1}}^{0}T\left(z,t\right){dz}$ and ${\int }_{({-H}_{1}+{H}_{2})}^{{-H}_{1}}T\left(z,t\right){dz}$ can be analyzed as integrated variables ${C}_{1}(t)$ and ${C}_{2}(t)$, representing the time-varying heat content of the surface and the subsurface layers. Then ${T}_{{{{\rm{mix}}}}}$ is given as:

9 $${T}_{{{{\rm{mix}}}}}=\frac{{C}_{1}\left(t\right)+{C}_{2}\left(t\right)}{{H}_{0}},$$

where ${H}_{0}$ denotes ${H}_{1}+{H}_{2}.$The correlations ($R$) of the post-mixing SST (${T}_{{{{\rm{mix}}}}}$) with each of the two initial layers’ temperature are calculated:

10 $$\left\{\begin{array}{c}R\left({T}_{{{{\rm{mix}}}}},{T}_{1}\right)=\frac{{{{\rm{Cov}}}}\left({T}_{{{{\rm{mix}}}}},{T}_{1}\right)}{\sigma {T}_{{{{\rm{mix}}}}}{{\cdot }}\sigma {T}_{1}}\\ R\left({T}_{{{{\rm{mix}}}}},{T}_{2}\right)=\frac{{{{\rm{Cov}}}}\left({T}_{{{{\rm{mix}}}}},{T}_{2}\right)}{\sigma {T}_{{{{\rm{mix}}}}}{{\cdot }}\sigma {T}_{2}}\end{array}\right.,$$

where ${T}_{1}$,${T}_{2}$ are assumed as the mean temperature of the surface and subsurface layer, respectively. Taking the calculation of the correlation with ${T}_{1}$ as an example, ${{{\rm{Cov}}}}({T}_{{{{\rm{mix}}}}},{T}_{1})$ is the covariance of ${T}_{{{{\rm{mix}}}}}$ and ${T}_{1}$, and $\sigma {T}_{{{{\rm{mix}}}}}$, $\sigma {T}_{1}$ are the standard deviations of ${T}_{{{{\rm{mix}}}}}$ and ${T}_{1}$. ${{{\rm{Cov}}}}({T}_{{{{\rm{mix}}}}},{T}_{1})$ can be derived as:

11 $$\begin{array}{c}{Cov}\left({T}_{{{{\rm{mix}}}}},{T}_{1}\right)={Cov}\left(\frac{{C}_{1}+{C}_{2}}{{H}_{0}},{T}_{1}\right),\\=\frac{1}{{H}_{0}}{Cov}\left({C}_{1},{T}_{1}\right)+\frac{1}{{H}_{0}}{Cov}\left({C}_{2},{T}_{1}\right),\\=\frac{1}{{H}_{0}}\sigma {C}_{1}{{\cdot }}\sigma {T}_{1}{{\cdot }}R\left({C}_{1},{T}_{1}\right)+\frac{1}{{H}_{0}}\sigma {C}_{2}{{\cdot }}\sigma {T}_{1}{{\cdot }}R\left({C}_{2},{T}_{1}\right).\end{array}$$

$\sigma {T}_{{{{\rm{mix}}}}}$ can be derived as:

12 $$\begin{array}{c}\sigma {T}_{{{{\rm{mix}}}}}^{2}={\left(\frac{\sigma {C}_{1}}{{H}_{0}}+\frac{\sigma {C}_{2}}{{H}_{0}}\right)}^{2},\\=\frac{1}{{H}_{0}^{2}}{\sigma }^{2}{C}_{1}+\frac{1}{{H}_{0}^{2}}{\sigma }^{2}{C}_{2}+\frac{2}{{H}_{0}}\sigma {C}_{1}{{\cdot }}\sigma {C}_{2}{{\cdot }}R\left({C}_{1},{C}_{2}\right),\\ \sigma {T}_{{{{\rm{mix}}}}}={\left[{\frac{1}{{H}_{0}^{2}}\sigma }^{2}{C}_{1}+\frac{1}{{H}_{0}^{2}}{\sigma }^{2}{C}_{2}+\frac{2}{{H}_{0}}\sigma {C}_{1}{{\cdot }}\sigma {C}_{2}{{\cdot }}R\left({C}_{1},{C}_{2}\right)\right]}^{\frac{1}{2}}.\end{array}$$

The correlation of ${T}_{{{{\rm{mix}}}}}$ and ${T}_{1}$ can be obtained from equations $\left(10\right)$, $\left(11\right)$ and $\left(12\right)$ as:

13 $$R\left({T}_{{{{\rm{mix}}}}},{T}_{1}\right)=\frac{\frac{1}{{H}_{0}}\sigma {C}_{1}{{\cdot }}\sigma {T}_{1}{{\cdot }}R\left({C}_{1},{T}_{1}\right)+\frac{1}{{H}_{0}}\sigma {C}_{2}{{\cdot }}\sigma {T}_{1}{{\cdot }}R\left({C}_{2},{T}_{1}\right)}{{\left[{\frac{1}{{H}_{0}^{2}}\sigma }^{2}{C}_{1}+\frac{1}{{H}_{0}^{2}}{\sigma }^{2}{C}_{2}+\frac{2}{{H}_{0}}\sigma {C}_{1}{{\cdot }}\sigma {C}_{2}{{\cdot }}R\left({C}_{1},{C}_{2}\right)\right]}^{\frac{1}{2}}{{\cdot }}\sigma {T}_{1}}.$$

Since the heat content of the subsurface layer is highly correlated with the core temperature of the water mass but is independent of the mixed-layer temperature, we have $R\left({C}_{1},{T}_{1}\right)\approx 1$ and $R\left({C}_{2},{T}_{1}\right)\approx 0$, therefore

14 $$R\left({T}_{{{{\rm{mix}}}}},{T}_{1}\right)\approx \frac{\sigma {C}_{1}}{{{H}_{0}\left[{\frac{1}{{H}_{0}^{2}}\sigma }^{2}{C}_{1}+\frac{1}{{H}_{0}^{2}}{\sigma }^{2}{C}_{2}+\frac{2}{{H}_{0}}\sigma {C}_{1}{{\cdot }}\sigma {C}_{2}{{\cdot }}R\left({C}_{1},{C}_{2}\right)\right]}^{\frac{1}{2}}}.$$

Similarly, the correlation between ${T}_{{{{\rm{mix}}}}}$ and ${T}_{2}$ is given by

15 $$R\left({T}_{{{{\rm{mix}}}}},{T}_{2}\right)\approx \frac{\sigma {C}_{2}}{{{H}_{0}\left[{\frac{1}{{H}_{0}^{2}}\sigma }^{2}{C}_{1}+\frac{1}{{H}_{0}^{2}}{\sigma }^{2}{C}_{2}+\frac{2}{{H}_{0}}\sigma {C}_{1}{{\cdot }}\sigma {C}_{2}{{\cdot }}R\left({C}_{1},{C}_{2}\right)\right]}^{\frac{1}{2}}}.$$

The ratio between them is given by:

16 $$\frac{R\left({T}_{{{{\rm{mix}}}}},{T}_{1}\right)}{R\left({T}_{{{{\rm{mix}}}}},{T}_{2}\right)}\approx \frac{\sigma {C}_{1}}{\sigma {C}_{2}},$$

that is, the ratio of the heat content standard deviations for the two layers. Based on previous results, ${C}_{1}$ is calculated as the heat content for the upper 60 m, and ${C}_{2}$ is calculated for the saline water (> 34.9 psu) mass above 150 m. While limited in depth, the latter was found to be highly correlated with the heat content of the entire water mass. The variances of the two components are respectively $\sigma {C}_{1}=$8.9 kJ cm^−2^, $\sigma {C}_{2}=$186.5 kJ cm^−2^. The subsurface layer’s heat content variability is approximately 20 times greater than that of the surface layer. This dominant variability allows the subsurface layer to control SST evolution, which may ultimately feed back onto the annual maximum LMI.

### Composite analyses of the surface oceanic and atmospheric conditions associated with TC intensity

we constructed composite analyses of SLP, wind stress, and MLD over the NPSH region for years with strong and weak TC activity. To objectively distinguish between strong and weak TC years, we used the standardized time series of annual maximum LMI (orange line in Fig. 4c). Years with standardized values greater than 0 were defined as strong TC events (1976, 1978−1986, 2009−2020), and those with values less than 0 as weak TC events (1975, 1977, 1987−2008). Composite analyses were then performed for the five years preceding each type of event to examine precursory signals in the ocean-atmosphere system.

### CMIP6 high-resolution models

The HighResMIP initiative is an integral component of CMIP6, aimed at enhancing model capabilities through improved horizontal resolution. To evaluate the ability of these high-resolution models to reproduce the observed relationship, we analyzed six models from HighResMIP^61^. The six high-resolution models are: Euro-Mediterranean Center on Climate Change coupled climate model version 2-VHR4 (CMCC-CM2-VHR4), Center National de Recherches Météorologiques model version 6.1-HR (CNRM-CM6−1-HR), PRocess-based climate sIMulation: AdVances in high-resolution modeling and European climate Risk Assessment (PRIMAVERA) version of European Community Earth System Model version 3P-HR (EC-Earth-3P-HR), European Center for Medium-Range Weather Forecasts-Integrated Forecasting System-HR (ECMWF-IFS-HR), Hadley Center Global Environmental Model 3-Global Coupled configuration 3.1-HM (HadGEM3-GC31-HM) and Max Planck Institute Earth System Model version 1.2-XR (MPI-ESM1-2-XR). Basic information for each model is provided in Supplementary Table 1.

The TC track and wind speed data from the six models used in our analysis were derived using the TRACK algorithm^78^. We utilize the simulated data derived from ocean-atmosphere coupled historical experiments (hist-1950). Although the hist-1950 simulations cover the period from 1950 to 2014, our analysis focuses on 1975−2014 to align with the temporal coverage of the observations. The corresponding monthly subsurface temperature and salinity data are then used to examine their association with the TC intensity simulated by the models.

## Supplementary information

Supplementary Information

Transparent Peer Review file

## Data Availability

The hurricane best-track data used in this study is the IBTrACS (v4r00) data, retrieved from the NOAA National Centers for Environmental Information https://www.ncei.noaa.gov/products/international-best-track-archive. The NCEP/NCAR Reanalysis 1 data can be downloaded from https://psl.noaa.gov/data/gridded/data.ncep.reanalysis.html. The OISST data can be downloaded at https://www.ncei.noaa.gov/products/optimum-interpolation-sst. The 137°E section data are publicly available at https://www.data.jma.go.jp/kaiyou/db/vessel_obs/data-report/html/ship/ship_e.php. A quality controlled and gridded subsurface temperature and salinity dataset (EN4) with a horizontal resolution of 1°$\times$1°, which is available from Met Office Hadley Center https://www.metoffice.gov.uk/hadobs/en4/download-en4-2-2.html#g10_profiles. The Argo data are collected and made freely available by the International Argo Program and the national programs http://www.argo.ucsd.edu. Six-hourly surface drifters are available from the Global Drifter Program at https://www.aoml.noaa.gov/phod/gdp/data.php. A global Mesoscale Eddy Trajectory Atlas (MET3.2 exp DT two-satellites, dataset provided by AVISO for eddy calculations can be downloaded from https://www.aviso.altimetry.fr/en/data/products/value-added-products/global-mesoscale-eddy-trajectory-product/meta3-2-dt.html. The monthly ERA5 data with a horizontal resolution of 0.25°$\times$0.25°, which is available on https://cds.climate.copernicus.eu/datasets/reanalysis-era5-single-levels-monthly-means?tab=overview. The monthly PDO index can be downloaded from https://www.ncei.noaa.gov/access/monitoring/pdo/. The climatological temperature and mixed layer depth filed from WOA2018 are publicly available on https://www.ncei.noaa.gov/products/world-ocean-atlas. Monthly ocean temperature and salinity data from CMIP6 models are available at https://esgf.ceda.ac.uk, and corresponding tropical cyclone data are available at https://data.ceda.ac.uk/badc/highresmip-derived/data/storm_tracks/TRACK.

## Code availability

The MITgcm model components are open source, which can be downloaded from https://mitgcm.org/source-code. The configuration of model simulations, the analysis code, and the source data for plotting can be obtained from https://zenodo.org/records/15853481. The MATLAB_R2025b is used for plotting.
