# Supplementary material for: Subsurface messenger for the annual maximum lifetime maximum intensity of tropical cyclones in the western North Pacific
Source: Nat Commun. 2026 May 7;17:6119. doi: 10.1038/s41467-026-72770-5 (PMC13358124; doi:10.1038/s41467-026-72770-5)
Supplement: Supplementary file 1 — Supplementary Information [file 41467_2026_72770_MOESM1_ESM.pdf]

Supplementary Materials for

Subsurface messenger for the annual maximum lifetime maximum intensity of tropical cyclones in the western North Pacific

Xinning Ni[1,2], Yu Zhang [1,2*], and Wei Wang [1,2*]

[1] Key Laboratory of Physical Oceanography and Frontiers Science Center for Deep Ocean Multispheres and Earth System/Academy of Future Ocean, Ocean University of China, Qingdao, China.

[2] Laboratory for Ocean Dynamics and Climate, Qingdao Marine Science and Technology Center, Qingdao, China.

*e-mail: yuz@ouc.edu.cn; wei@ouc.edu.cn

Content

Supplementary Figure 1–16

Supplementary Table 1

Supplementary References

Supplementary Figures

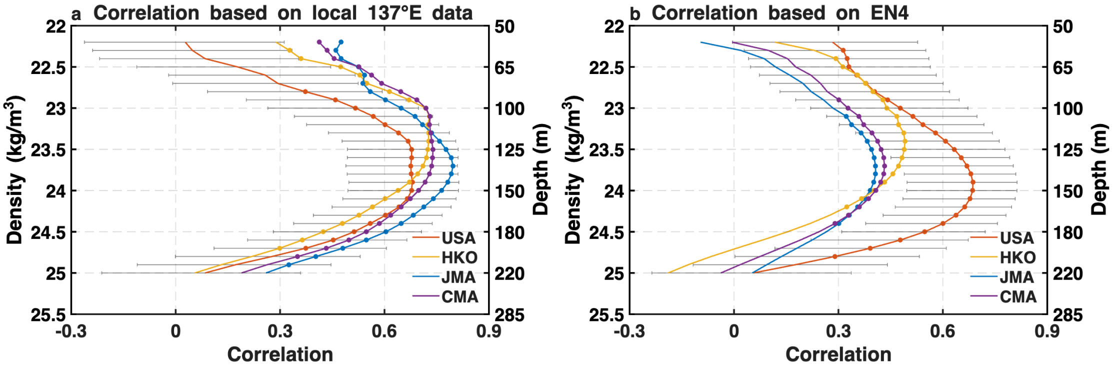

Supplementary Fig. 1. Correlation between annual maximum lifetime maximum intensity (LMI) and subsurface temperature. (a) Correlations between the annual maximum LMI and the temperature through the water column based on 137°E data. Temperature is extracted from a 4° latitudinal band centered on location of maximum LMI. **(b)** Correlations between the annual maximum LMI and the temperature through the water column based on EN4 data. The EN4 data are averaged over 120°–150°E, 14°–21°N. Colored markers indicate statistically significant results ($p < 0.05$). Gray bars represent the 95% confidence intervals based on the IBTrACS-USA dataset.

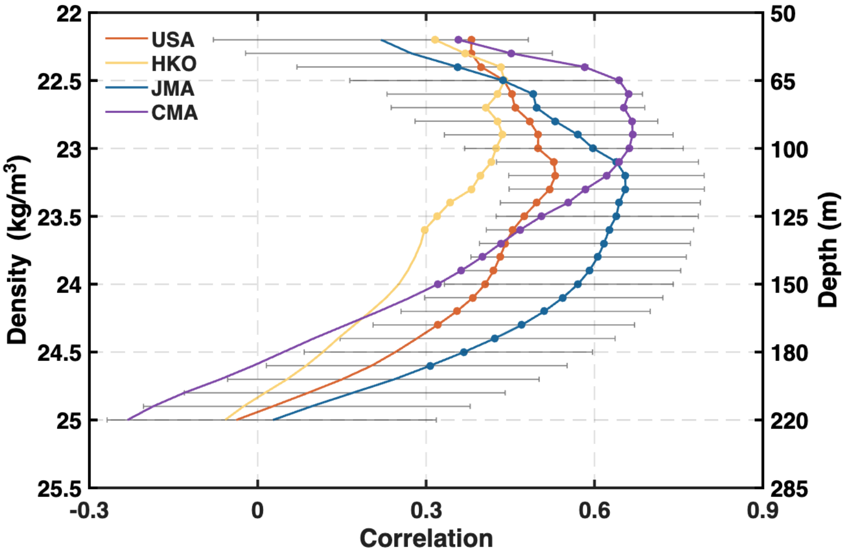

Supplementary Fig. 2. Correlation between tropical cyclone intensity increment and subsurface temperature. Colored markers indicate statistically significant results ($p < 0.05$). Gray bars represent the 95% confidence intervals based on the IBTrACS-JMA dataset.

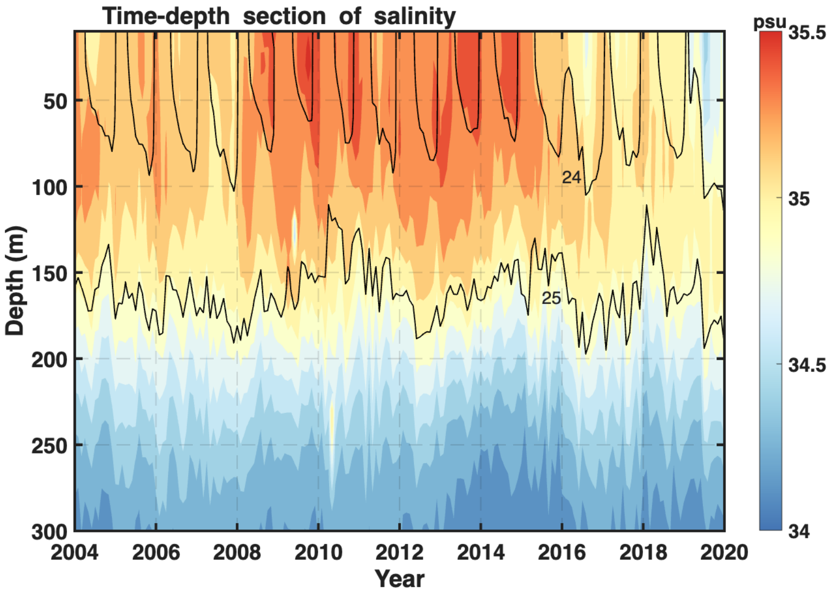

Supplementary Fig. 3. Water mass ventilated in the eastern North Pacific. Time-depth section of salinity (shade) in the region (200°–210°E, 23°–28°N) based on Argo data. Black lines represent isopycnals 24 kg m^{-3} and 25 kg m^{-3}.

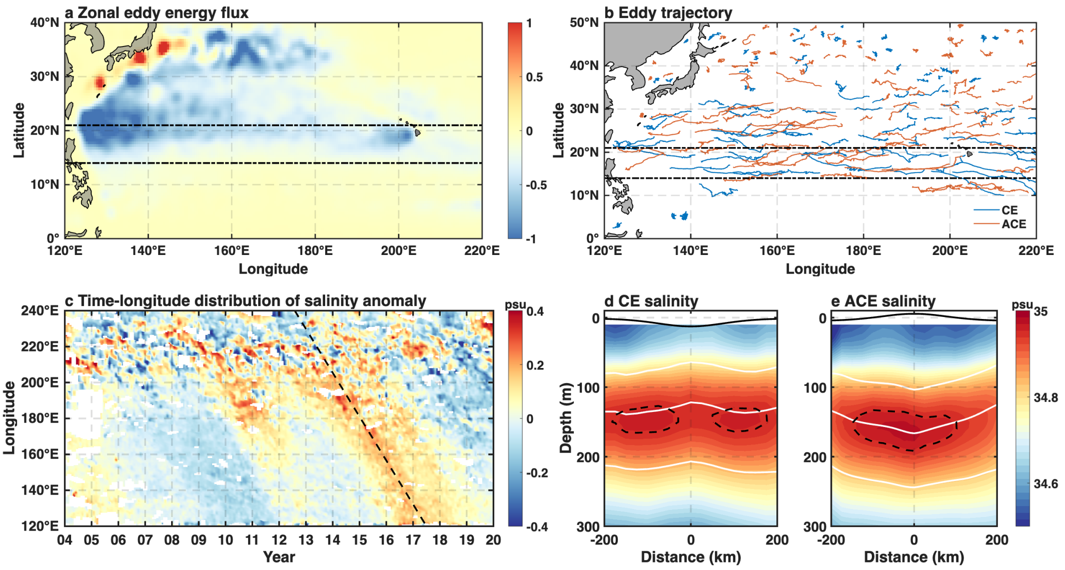

Supplementary Fig. 4 The process of mesoscale eddies carrying subsurface water westward. (a) Distribution of zonal flux of eddy potential energy. Energy flux is normalized by dividing their maximum value. **(b)** Trajectories of eddies, with life span exceeding 120 days, from June 2015 to December 2015. Red (blue) lines represent trajectories of anticyclonic (cyclonic) eddies. **(c)** Time-longitude distribution of salinity anomaly along the isopycnal 24.5 kg m^{-3} (14°–21°N) based on Argo data. **(d)** Composite salinity distribution inside cyclonic eddies (CEs). **(e)** Composite salinity distribution inside anticyclonic eddies (ACEs). The composite analysis in **(d)** and **(e)** is within 120°–150°E, 14°–21°N. The white lines represent isopycnals 23.0 kg m^{-3}, 24.0 kg m^{-3} and 25.0 kg m^{-3}. The black dashed contour denotes salinity > 34.95 psu. The black solid lines illustrate the sea surface height anomaly of eddies but amplified by 100 times.

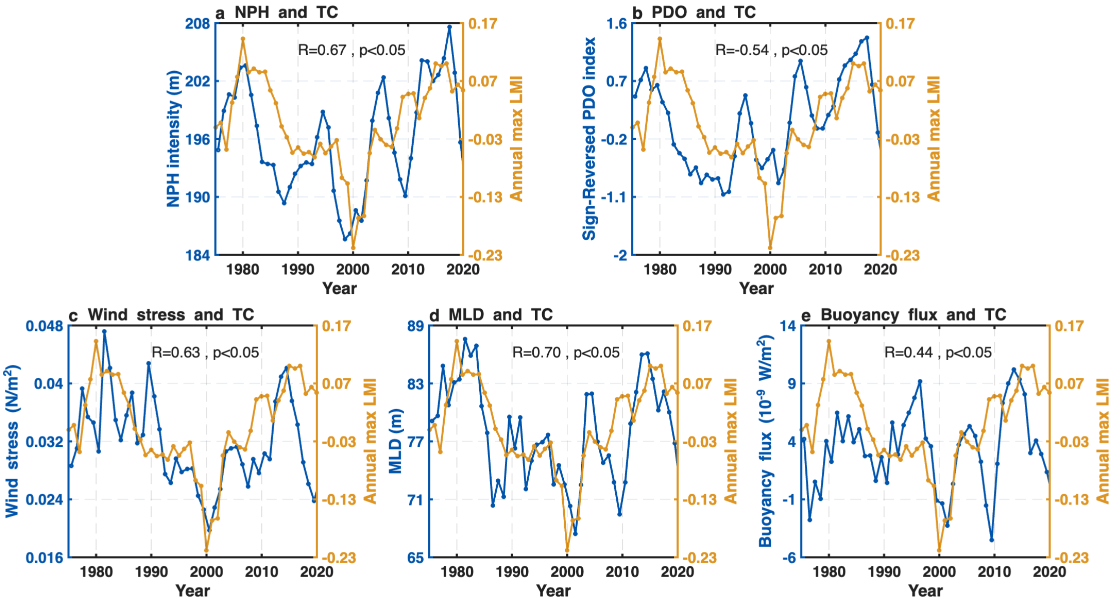

Supplementary Fig. 5 Relationship between the annual maximum lifetime maximum intensity (LMI) of tropical cyclones (TCs) and various variables that potentially influence the formation and subduction of the water mass. Time series of annual maximum LMI and **(a)** North Pacific High (NPH) intensity; **(b)** Pacific Decadal Oscillation (PDO) index; **(c)** March wind stress averaged over the water mass formation region; **(d)** January–March mean mixed layer depth (MLD) averaged over the formation region;**(e)** January–March mean buoyancy flux averaged over the formation region. The LMI time series is averaged from four agencies (USA, HKO, CMA and JMA) and is normalized. A 3-year smoothing was applied to time series. The water mass formation region is defined as 200°–240°E, 20°–35°N. In each panel, the time series of the influencing variable (blue) is shifted to the right by 5.5 years, and the corresponding lead correlation coefficient is indicated.

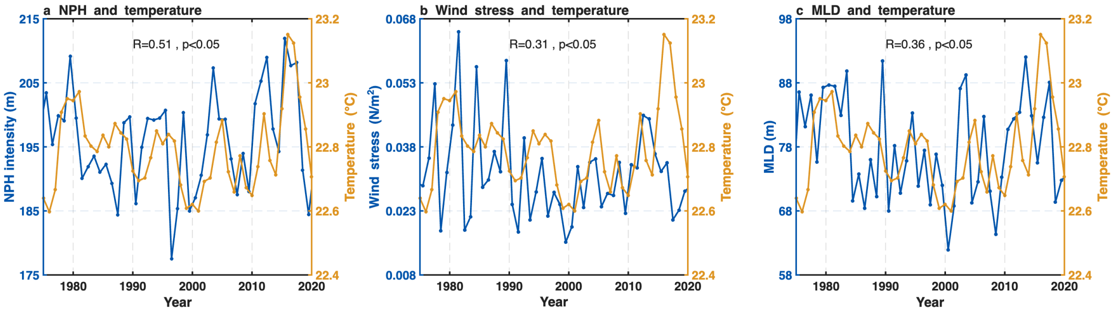

Supplementary Fig. 6. Relationship between the subsurface temperature and various variables. Time series of subsurface temperature along the isopycnal 24 kg m^{-3} in the TC region (120°–150°E, 14°–21°N) and **(a)** North Pacific High (NPH) intensity; **(b)** March wind stress averaged over the water mass formation region (200°–240°E, 20°–35°N); **(c)** January–March mean mixed layer depth (MLD) averaged over the formation region. A 1-year smoothing was applied to all time series. In each panel, the time series of the influencing variable (blue) is shifted to the right by 4.5 years, and the corresponding lead correlation coefficient is indicated.

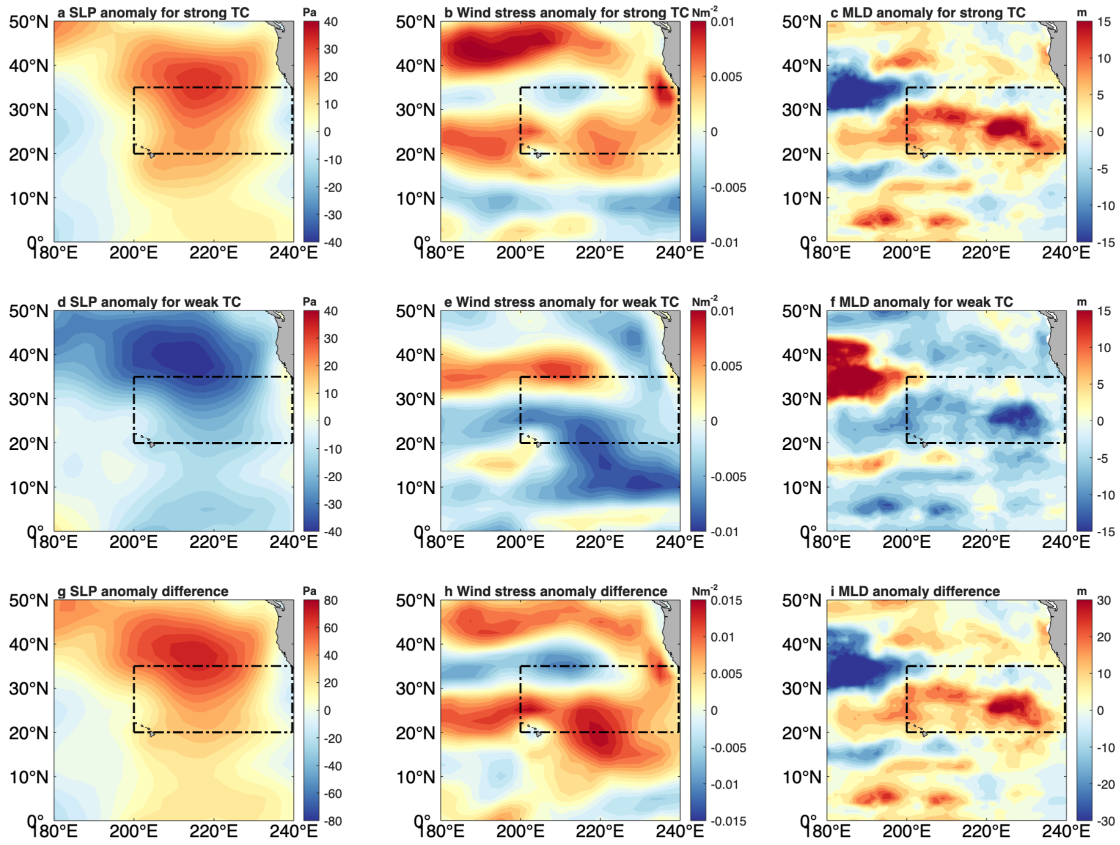

Supplementary Fig. 7. Composite analysis of ocean and atmospheric conditions five years prior to strong and weak tropical cyclone (TC) events. Shown are sea level pressure (SLP) anomaly **(a, d, g)**, March wind stress anomaly **(b, e, h)**, and January–March mean mixed layer depth (MLD) anomaly **(c, f, i)**. The composite fields are shown for **(a–c)** strong TC events, **(d–f)** weak TC events, and **(g–i)** their anomaly differences (strong minus weak). The black dashed box (200°–240°E, 20°–35°N) denotes the core water mass formation area.

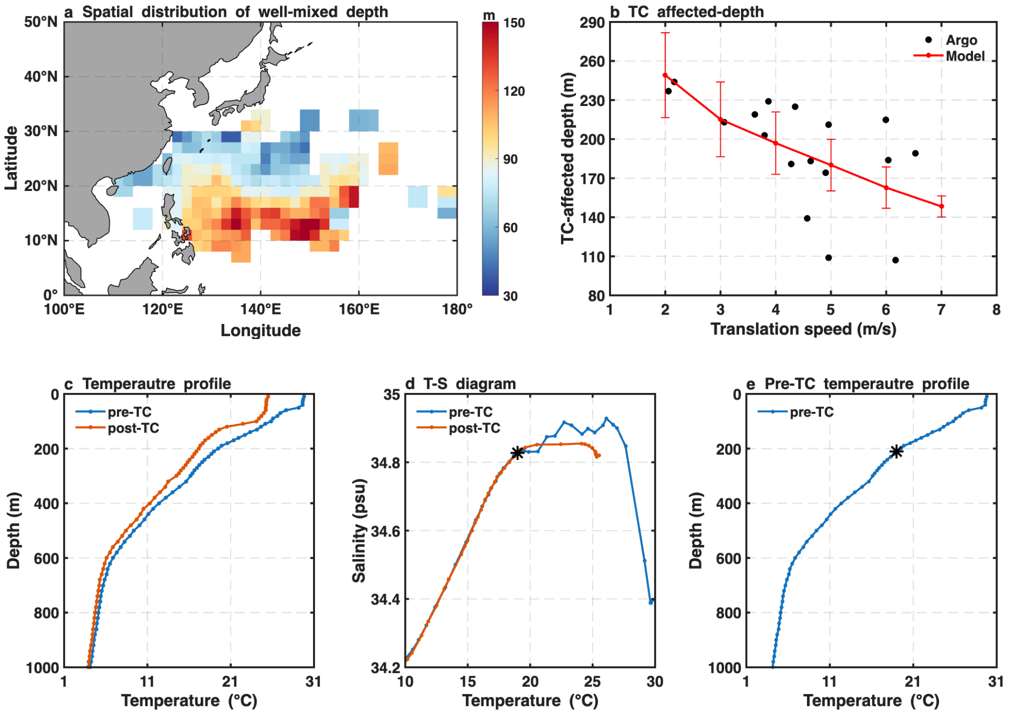

Supplementary Fig. 8. Well-mixed depth and tropical cyclone (TC)-affected depth induced by strong TCs. (a) Spatial distribution of well-mixed depth induced by TCs (Methods). **(b)** TC-affected depth as a function of translation speed (method details shown in panels **(c)** – **(e)**). Argo profiles are sampled within 0–7 days after TC passage and within 50 km across TC track, considering only strong TCs (Category 4 and 5). The model simulation is forced with a wind speed of 80 m s^{-1}. Red lines show the mean value within 50 km across the track, averaged within 0–7 days after TC passage, with error bars indicating one standard deviation. **(c)** Comparison of temperature vertical profiles at 8.5 days before (blue) and 1.5 days after (red) the passage of TC Choi-wan in September, 2009. **(d)** Temperature-Salinity (T-S) profiles at 8.5 days before (blue) and 1.5 days after (red) the passage of TC Choi-wan. The black star denotes where the two profiles start to diverge. **(e)** The temperature profile before the passage of TC Choi-wan with the star denoting the TC-affected depth obtained from **(d)**.

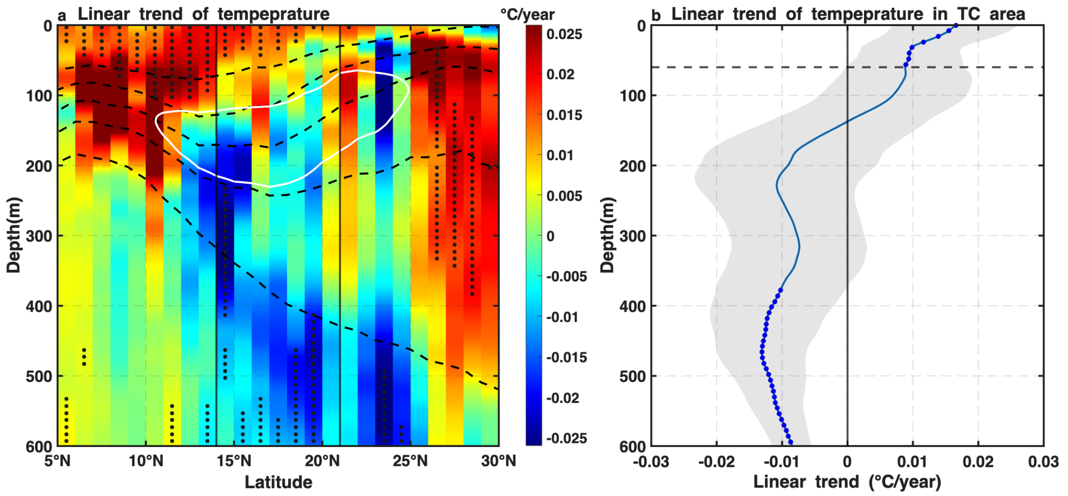

Supplementary Fig. 9. Linear trends of temperature based on 137°E dataset from 1975 to 2020. (a) Depth-latitude distribution of the temperature linear trend (shading). The black dashed contours denote isopycnals from 22 to 26 kg m^{-3} at intervals of 1 kg m^{-3}. Waters with salinity > 34.9 psu is bounded by the white contour. Black dots show results that are statistically significant ($p < 0.05$). Black solid lines denote 14°N and 21°N respectively. **(b)** Linear trend of temperature averaged over 14°–21°N in tropical cyclone (TC) area. Blue dots show results that are statistically significant ($p < 0.05$), and the gray shading represents the 95% confidence intervals. The black dashed line denotes 60 m depth.

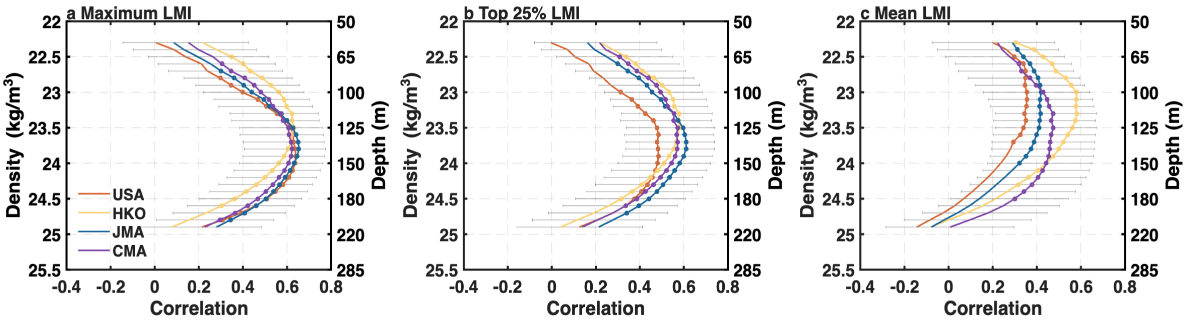

Supplementary Fig. 10. Correlation between tropical cyclone intensity and ocean temperature. Results are for **(a)** Maximum lifetime maximum intensity (LMI); **(b)** Mean LMI of the top 25% and **(c)** Mean LMI of all TCs. Colored markers indicate statistically significant results ($p < 0.05$). Gray bars represent the 95% confidence intervals based on the IBTrACS-CMA dataset. A 3-year running mean was applied to all time series.

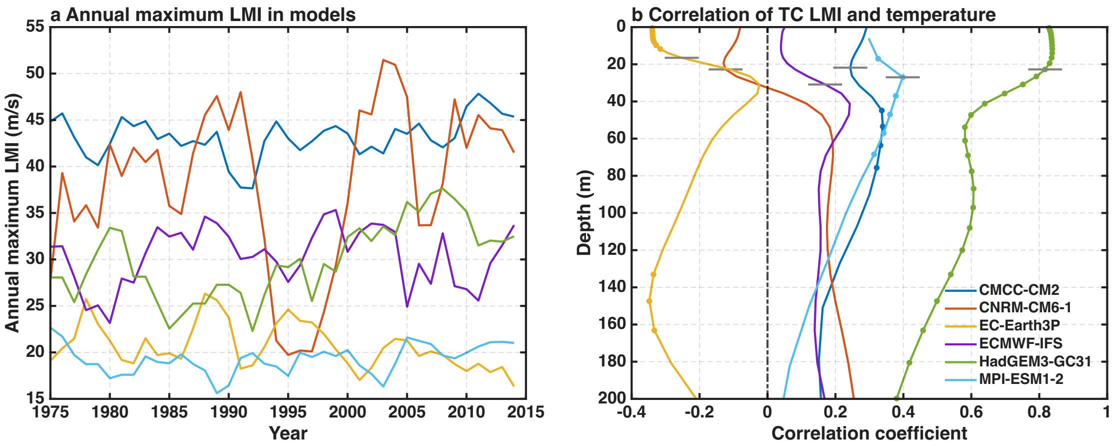

Supplementary Fig. 11. Relationship between annual maximum lifetime maximum intensity (LMI) of tropical cyclones and ocean temperature in HighResMIP models during 1975–2014. (a) Time series of the annual maximum LMI within the region 120°–150°E, 14°–25°N for six models. **(b)** Simultaneous correlation coefficients between the annual maximum LMI and the temperature through the water column for six models. Temperature is averaged over the same region 120°–150°E, 14°–25°N. Color dots show results statistically significant ($p < 0.05$). Gray bars denote the mixed layer depth in each model. A 3-year running mean smoothing was applied to all time series.

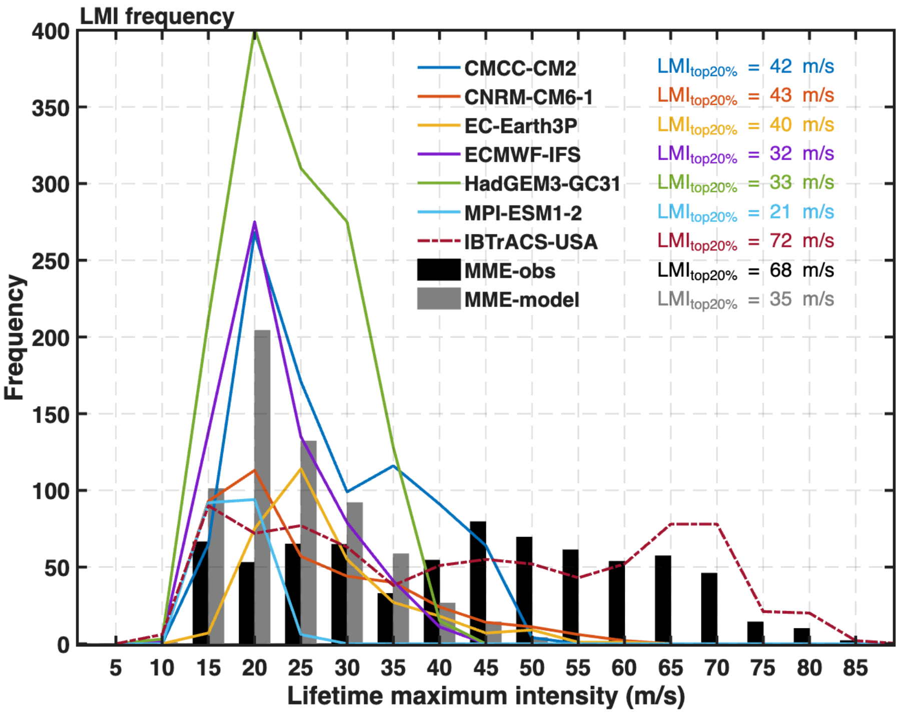

Supplementary Fig. 12. Comparison of tropical cyclone lifetime maximum intensity (LMI) distribution between observations and HighResMIP models in the western North Pacific (WNP) during 1975–2014. The intensity distribution is based on all storms in the WNP (120°–180°E, 0°–30°N). Colored solid lines represent the six individual HighResMIP models, and the gray bars indicate their multi-model ensemble mean (MME). Black bars represent the MME of four observational datasets (USA, CMA, HKO, JMA) from IBTrACS. The top 20% mean LMI for each model is denoted in the upper-right corner of the panel.

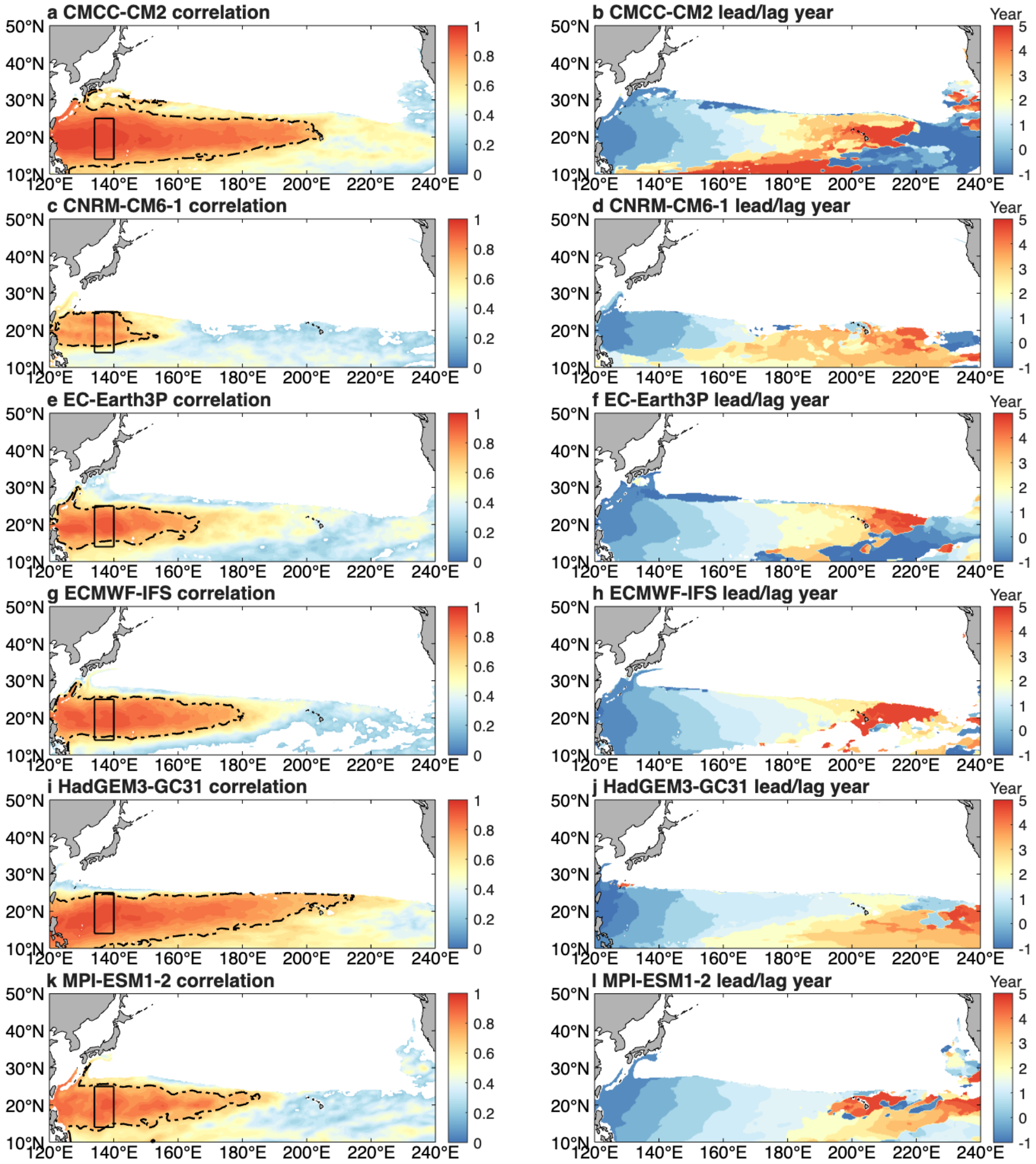

Supplementary Fig. 13. The transport path of the subsurface water in HighResMIP models. (a, c, e, g, i, k) Strongest correlations (above 95% confidence level) between the temperature within the black box (134°–140°E, 14°–25°N) and that of all other regions along the isopycnal 24.5 kg m^{-3}. The black dashed contour denotes a correlation of 0.7. **(b, d, f, h, j, l)** The corresponding lead/lag time at which the strongest correlation occurs. The black dashed contour denotes a correlation of 0.7. Results are shown for: **(a, b)** CMCC-CM2; **(c, d)** CNRM-CM6-1; **(e, f)** EC-Earth3P; **(g, h)** ECMWF-IFS; **(i, j)** HadGEM3-GC31; **(k, l)** MPI-ESM1-2.

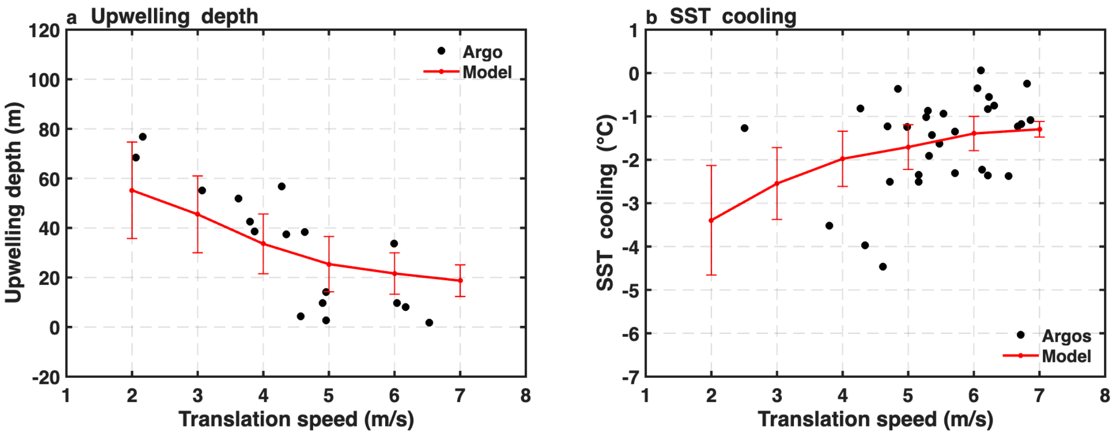

Supplementary Fig. 14. Verification of model-induced upwelling and sea surface temperature (SST) cooling. (a) Observed upwelling depth based on Argo (black dots) and model-simulated upwelling depth (red). **(b)** Observed SST cooling based on surface drifters (black dots) and model-simulated SST cooling (red). Argo and surface drifters are sampled within 0–7 days after tropical cyclone (TC) passage and within 50 km across the TC track, considering only strong TCs (Category 4 and 5). The model simulation is forced with a wind speed of 80 m s^{-1}. Red lines show the mean value within 50 km across the track, averaged within 0–7 days after TC passage, with error bars indicating one standard deviation.

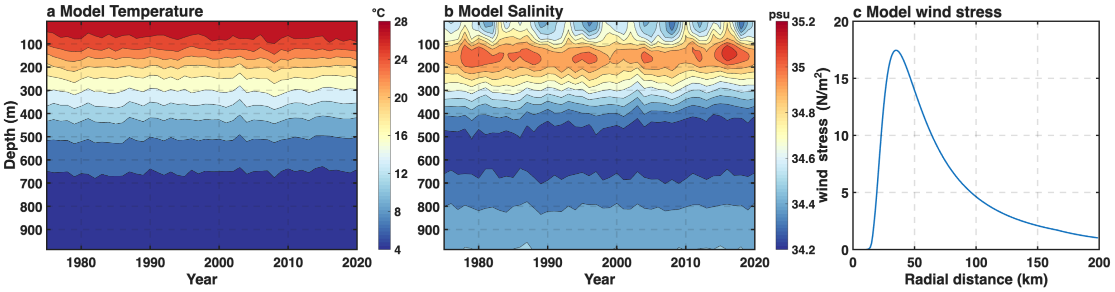

Supplementary Fig. 15. Initial model configuration. (a) Temperature profile from 1975–2020 based on 137°E section data, **(b)** the same as **(a)** but for the salinity profile. **(c)** Radial distribution of the wind stress for the model TC.

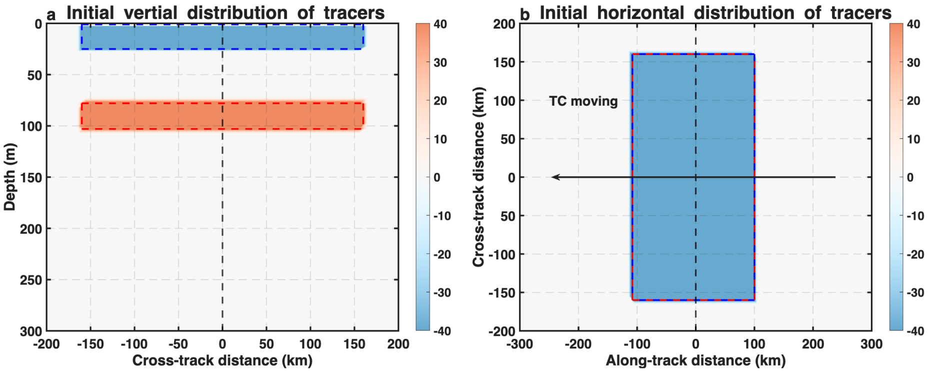

Supplementary Fig. 16. Initial tracer displacement. (a) Cross-track and vertical section of the tracer distribution initially located around 20 m (blue shade) and 90 m (red shade). **(b)** Horizontal distribution of the tracer at around 20 m.

Supplementary Table 1. Concise list of HighResMIP model configurations.

Model name	Atmospheric resolution (km)	Ocean resolution (°)	Ocean model levels	Reference
CMCC-CM2-VHR4	25	0.25	50	Scoccimarro et al., 2017
CNRM-CM6-1-HR	50	0.25	75	Voldoire, 2019
EC-Earth3P-HR	50	0.25	75	EC-Earth, 2018
ECMWF-IFS-HR	25	0.25	75	Roberts et al., 2017
HadGEM3-GC31-HM	50	0.25	75	Roberts, 2017
MPI-ESM1-2-XR	50	0.4	40	von Storch et al., 2017

Supplementary References:

1. Scoccimarro, E., Bellucci, A., & Peano, D. CMCC CMCC-CM2-VHR4 model output prepared for CMIP6 HighResMIP. *Earth System Grid Federation* https://doi.org/10.22033/ESGF/CMIP6.1367 (2017).
2. Voldoire, A. CNRM-CERFACS CNRM-CM6-1-HR model output prepared for CMIP6 HighResMIP. *Earth System Grid Federation* https://doi.org/10.22033/ESGF/CMIP6.1387 (2019).
3. EC-Earth Consortium. EC-Earth-Consortium EC-Earth3P-HR model output prepared for CMIP6 HighResMIP. *Earth System Grid Federation* https://doi.org/10.22033/ESGF/CMIP6.2323 (2018).
4. Roberts, C. D., Senan, R., Molteni, F., et al. ECMWF ECMWF-IFS-HR model output prepared for CMIP6 HighResMIP. *Earth System Grid Federation* https://doi.org/10.22033/ESGF/CMIP6.2461 (2017).
5. Roberts, M. MOHC HadGEM3-GC31-HM model output prepared for CMIP6 HighResMIP. *Earth System Grid Federation* https://doi.org/10.22033/ESGF/CMIP6.446 (2017).
6. von Storch, J.-S., Putrasahan, D., Lohmann, K., et al. MPI-M MPI-ESM1.2-XR model output prepared for CMIP6 HighResMIP. *Earth System Grid Federation* https://doi.org/10.22033/ESGF/CMIP6.10290 (2017).
