# Supplementary material for: Subsurface messenger for the annual maximum lifetime maximum intensity of tropical cyclones in the western North Pacific
Source: Nat Commun. 2026 May 7;17:6119. doi: 10.1038/s41467-026-72770-5 (PMC13358124; doi:10.1038/s41467-026-72770-5)
Supplement: Supplementary file 2 — Transparent Peer Review file [file 41467_2026_72770_MOESM2_ESM.pdf]

Peer Review File

Subsurface messenger for the annual maximum lifetime maximum intensity of tropical cyclones in the western North Pacific

Corresponding Author: Dr Yu Zhang

This file contains all reviewer reports in order by version, followed by all author rebuttals in order by version.

Attachments originally included by the reviewers as part of their assessment can be found at the end of this file.

Version 0:

Reviewer comments:

Reviewer #1

(Remarks to the Author)
In this study, Ni et al. show using a combination of observations and numerical experiments that the intensity of TCs in the Western Pacific is primarily controlled by sub-surface water temperatures. Further they also show that these waters are subducted from the Eastern Pacific 3-4 years earlier. The first half of this work is devoted to showing the relevance of sub-surface ocean temperature for TC intensification, which I believe has been demonstrated already in previous studies and is not surprising. However, the second half of the work, where they show the link between sub-surface waters in the TC region and the subduction process is more interesting and novel. Also, it has practical implications as it is related to predictability. If the authors can show that surface ocean conditions in the subduction region of Eastern Pacific directly controls TC activity in the Western Pacific at lead times of 3-4 years, it'll make the study much more impactful. Other comments and suggestions for improving the study are provided below.

Lines 76-80: I'm not sure this statement is entirely accurate. Previous studies have indeed demonstrated the significance of the ocean sub-surface thermal structure for Eastern Pacific TCs (Balaguru et al., 2013; Jin, F-F. et al., 2014).

Fig. 1a: Do you include all TC track locations in this? If that's the case, I would suggest trying with only those track locations where the wind speeds are higher. I'm not sure locations where the TC winds are weak (eg. Tropical Storm, Cat 1-2) the storms can mix down to a depth of ~200 m.

The results presented in Fig. 2a and 2b are consistent with Lin, I-I et al. (2013), who showed the importance of the sub-surface ocean temperature in controlling the LMI of TCs in the western Pacific.

In Figs. 1b, 2a, 2b and 2d, please indicate uncertainty bounds. Also, the statistical significance of correlation coefficients must be indicated in Fig. 2c.

Also, in Fig. 2c, I'd replace SLP with mid-level relative humidity. While the role of atmospheric moisture in modulating TC intensity is well-known, it is unclear to me how SLP affects TCs (unless we're talking about SLP in the context of subtropical high pressure and steering flow).

Lines 292-301: This analysis is quite interesting, as it relates to predictability. While the authors show that the sub-surface conditions in the TC region are correlated to surface conditions in the subduction region with a lead time of 3-4 years, can they extend this to TCs directly? In other words, if they use a metric like ACE in the TC region, how well is this correlated with surface conditions in the subduction region 3-4 years prior?

Lines 347-349: While mixing is always important, upwelling typically plays a role only in slow-moving TCs.

(Remarks on code availability)

Reviewer #2

(Remarks to the Author)
Attached as pdf.

[Editorial Note: See end of file]

(Remarks on code availability)

Reviewer #3

(Remarks to the Author)
Review of "The subsurface messenger for the maximum intensity of tropical cyclones in the western North Pacific" by Ni et al.

The manuscript shows an interesting correlation between an annual time series of TC lifetime maximum intensity and ocean temperature at depths of about 100-200 m. Numerical ocean model experiments are conducted to show that super TCs (LMI>70 m/s) cause deep mixing and upwelling, consistent with the observed relationship between subsurface temperature and LMI. Examination of basin-wide subsurface salinity shows a connection between conditions in the TC region and water mass formation in the northeastern Pacific, providing a possible explanation for the variations of subsurface temperature and LMI in the TC region.

The results are interesting and potentially important for predicting the occurrence of strong typhoons in the western Pacific, if ocean conditions in the northeastern Pacific do lead those in the TC region by several months and subsurface temperature has a strong impact on TC-induced ocean cooling, TC intensity, and LMI. However, the manuscript does not conclusively show either of these. Much of the main analysis is based on correlations (e.g., covariability of subsurface temperature with LMI) without in-depth physical reasoning. The mechanism of subsurface temperature variability in the TC region (propagation from the northeastern Pacific) is inconclusive because the subsurface salinity propagation pathway is shown, not temperature. These major issues need to be addressed.

1. Evidence of the importance of subsurface temperature for super TCs.
It's well known that SST is the most important factor affecting TC intensity. Subsurface conditions (ocean heat content, T/S stratification) affect TCs through their impact on SST. How can subsurface temperature (150 m depth) be more important than SST? I'm not aware of any other studies that come to a similar conclusion except maybe for specific case studies. In the present study, annual maximum LMI (defined as max TC intensity achieved by any TC in a given year?) is used instead of intensity, and LMI seems like a less robust metric compared to integrated/averaged metrics like ACE. More in-depth analysis is needed to explain why subsurface temperature is so much more important than SST. Is SST very uniform spatially and/or does is not change much year to year? If subsurface temperature has stronger variability than SST, that could help explain it. However, SST is 100% effective at modifying the surface enthalpy flux and TC intensity (any change in SST directly affects the energy exchange), whereas subsurface temperature, especially as deep as 100-200 m, is much less effective because it first has to be mixed/upwelled to the surface. If a warm temperature anomaly at 100 m depth is 1C and it decreases linearly to the surface, and the TC mixes completely down to 100 m, the subsurface temperature anomaly will result in an SST anomaly of only 0.5C. That means variability of subsurface temperature has to be twice as large as variability of SST just for subsurface temperature to have a similar impact as SST. However, you show in Fig. 2c that the influence of subsurface temperature dominates that of SST, and the maximum correlation is at ~150 m depth. That means that subsurface temperature variability has to be a lot larger than that of SST, probably at least 5-6 times larger. Is that the case?

Another possibility is that subsurface temperature is correlated with something else that affects LMI, such as TC atmospheric steering flow. Changes in the large-scale flow could keep TCs over more or less conducive conditions for intensification for longer or shorter amounts of time, resulting in changes in LMI that are physically unconnected to changes in subsurface temperature. At the same time, the steering flow anomalies could also force subsurface temperature changes through Ekman convergence/divergence, resulting in the observed correlation without causation.

2. Evidence of subsurface temperature propagation to the TC region.
The analysis showing interannual variations in subsurface salinity in the TC region that are connected to formation regions in the northeastern Pacific makes sense. However, the argument made throughout the manuscript is that changes in subsurface temperature strongly affect TC LMI, not subsurface salinity. Does subsurface temperature show similar lat-lon distributions and lead-lag relationships as salinity? This is crucial to the argument of remote forcing and predictability of super TC activity in the western North Pacific.

Other comments:

Line 89: Please define 'super TC' (LMI > 70 m/s).

Lines 101-104: Please explain why LMI has the most direct relationship with regional oceanic thermal conditions. For a given TC, it will depend strongly on atmospheric conditions such as wind shear and humidity. The maximum LMI over a whole year might also be very sensitive to atmospheric conditions. For example, if the ocean is the same in two different years, but a TC happens to experience much more conducive atmospheric conditions along its track in one year compared to the other (because of steering flow and/or synoptic weather variations), that year will have a higher total LMI despite no differences in the ocean. These subtleties deserve more consideration and discussion.

Fig. 1: Another interpretation is that TCs form to the south and generally move to the west/northwest and take time to reach max. intensity. That's why strong TCs are most common in the 15-20N region.

There are different metrics used in the manuscript and they are not always well defined: super TC strength ("maximum LMI of all TCs attained in each year" - maximum LMI across all TCs (a single value) or LMI averaged across all TCs?), "annual maximum LMI" (Fig. 2 caption), "maximum LMI" (line 204). More generally, the use of "maximum" to describe LMI is confusing because LMI is "lifetime maximum intensity." What does "maximum lifetime maximum intensity" mean?

Lines 334-336: I don't understand the statement "underlying SST has already lost its pre-TC signature." The pre-storm SST still matters, whether it's 27C or 29C. The TC-induced cooling starts from the pre-storm value. If pre-storm SST is lower, under-storm SST will be lower. In that respect, the pre-storm SST signature remains. Even if the impact of SST on TC intensification is nonlinear, for example if SST=24 and SST=26 have the same effect, the pre-storm SST helps determine whether that nonlinear threshold will be reached. On the other hand, if super TCs are so strong that they always bring SST below the nonlinear threshold, then variations of subsurface temperature will not impact TC intensity.

Line 639: A translation speed of 3 m/s if very low. The observed mean translation speed is probably closer to 6 m/s. Therefore, please verify that the modeled upwelling agrees well with the upwelling estimated from the Argo profiles. Please also verify that the model produces TC-induced SST cooling that is similar to observed values.

Lines 655-656: "disabling horizontal advection" - In the main text it's stated that horizontal divergence is set to zero. Which one is it?

(Remarks on code availability)

Version 1:

Reviewer comments:

Reviewer #1

(Remarks to the Author)
The authors have addressed my comments from the last round of reviews fairly well. I only have two additional suggestions for them.

1) It is nice that they have shown the relationship between the North Pacific high in the subtropical eastern Pacific and TC activity in the western Pacific. While they have used observations to demonstrate this, I wonder if they can confirm the robustness of this relationship (eg. ~5 years) using models. One possibility is the use of TC tracks from the HighResMIP suite of models, which are already provided here:
https://catalogue.ceda.ac.uk/uuid/e82a62d926d7448696a2b60c1925f811/.

2) To complement the existing analysis in Fig. 4, I suggest showing composites of surface pressure (and winds/MLD) over the NPSH region for years with strong and weak TCs in the western Pacific. Beyond a simple correlation, this would help see things a bit more clearly.

(Remarks on code availability)

Reviewer #3

(Remarks to the Author)
Second review of "Subsurface messenger for the annual maximum LMI of tropical cyclones in the western North Pacific" by Ni et al.

The authors have responded very well to the reviewers' comments and have provided more robust and in-depth analyses to support the main conclusions. These include (1) comparisons of the impact of subsurface temperature on maximum LMI to the impacts of other environmental conditions such as wind shear, humidity, and vorticity, showing that subsurface temperature is most important; (2) analysis of atmospheric forcing of subduction in the eastern Pacific and the connection to subsurface temperature in the western Pacific; (3) quantitative analysis supporting the greater impact of subsurface temperature variability on under-TC SST compared to pre-storm SST.

I have a few more comments aimed at making the analysis and conclusions even more robust:

Lines 80-91: Why not use an upper quantile (90% or 95%) as in previous studies (Elsner et al. 2008)? Using the maximum intensity achieved by any single TC in a year can be strongly affected by any random confluence of synoptic weather conditions. For example, this was likely the case for Hurricane Patricia in the eastern Pacific, which was a very small hurricane and had nearly ideal SST and atmospheric conditions as it moved quickly, making subsurface ocean conditions unimportant. Cases like that probably don't have a very big impact on decadal variability in the western Pacific, which has high annual TC counts, but it would be helpful to know that your results are not sensitive to the TC intensity treshold for reasonable choices (top 10%, 5%, 1% of LMI, for example).

The variable that connects changes in subsurface temperature to LMI is TC-induced SST cooling (i.e., cold wake). With satellite SST, this can be calculated accurately since 1982. Are SST cold wakes weaker when max LMI is higher? It would be helpful to see a plot like Fig. 2d for max LMI and average cold wake to make a direct connection between subsurface temperature and max LMI.

What is the average translation speed for TCs with max LMI in the region you consider? This would help to put the mixing depths in context (Figs. S7, S9).

Lines 53-55: The interplay of these different factors for SST cooling is also described nicely in this recent paper: doi:10.1029/2024JC021610.

(Remarks on code availability)

Version 2:

Reviewer comments:

Reviewer #1

(Remarks to the Author)
The authors have adequately responded to my comments, I have no further suggestions for them.

(Remarks on code availability)

Reviewer #3

(Remarks to the Author)
The authors have responded well to the last round of reviews and further strengthened the manuscript. I recommend publication.

(Remarks on code availability)

Open Access This Peer Review File is licensed under a Creative Commons Attribution 4.0 International License, which permits use, sharing, adaptation, distribution and reproduction in any medium or format, as long as you give appropriate credit to the original author(s) and the source, provide a link to the Creative Commons license, and indicate if changes were made.
In cases where reviewers are anonymous, credit should be given to 'Anonymous Referee' and the source.
The images or other third party material in this Peer Review File are included in the article’s Creative Commons license, unless indicated otherwise in a credit line to the material. If material is not included in the article’s Creative Commons license and your intended use is not permitted by statutory regulation or exceeds the permitted use, you will need to obtain permission directly from the copyright holder.
To view a copy of this license, visit https://creativecommons.org/licenses/by/4.0/

We would like to express our sincere gratitude to the reviewers for their thoughtful and constructive feedback. Their insights have been invaluable in improving the quality of our manuscript. In response to the collective comments, we have made significant revisions throughout the manuscript. Below, we provide a general summary of the main themes that emerged from the reviewers' feedbacks, followed by a detailed point-by-point response to each individual reviewer's comments.

General Response

1.Primary objective of our study

First and foremost, we would like to restate the primary objective of our study to provide context for the revisions we have made and to clarify how the reviewers' feedback has influenced our approach. Our central focus is understanding how the strongest tropical cyclones (TCs), those with the most catastrophic impacts on human populations---evolve under a changing climate over the past four decades (1975-2020), and the role that the ocean plays in this process. Since strong TCs are characterized by intense turbulent mixing and upwelling that bring large amounts of subsurface water to the surface, the state of the subsurface ocean is significant to their development. Our goal is to identify both the general phenomena and the dominant mechanisms regarding the impact of the subsurface ocean on the strongest TCs. As highlighted by Reviewer 1 and Reviewer 2, many studies have suggested the significance of subsurface conditions to TCs. Several foundational works including Lin et al. 2013, Kang et al. 2024, Balaguru et al. 2013 and Jin et al. 2014, established that the TC activity is more closely related to the overall pre-storm upper ocean heat content such as T80 (average temperature for the upper 80 m) in Lin et al. 2013 and T105 in Jin et al. 2014, rather than the pre-storm sea surface temperature (SST) alone. These prior studies have focused on the average behavior of TCs across different categories (both strong and weak), such as the lifetime maximum intensity of all TCs in Lin et al. 2013, mean intensification rate in Kang et al. 2024, PDI (Power Dissipation Index) in Balaguru et al. 2013 and ACE (Accumulated Cyclone Energy) in Jin et al. 2014. That said, compared to the approaches employed by these pioneering studies, ours differ in two major aspects.

(1) We focus on a TC key region with a specific subsurface water mass.

The region studied is located between 120°-150°E and 14°-21°N, along the northern edge of the North Equatorial Current (NEC). This region experiences high frequency of the most intense TCs (Fig. 1). For example, regarding TCs with LMI exceeding 70 m/s in the western North Pacific, 82 out of 123 since year 1980 and 47 out of 72 since year 2000 occurred in this area (based on IBTrACS-USA data).

In addition, this area also overlies a subsurface water mass characterized by high temperature and high salinity. These properties create favorable thermodynamic conditions for TC intensification. More importantly, the thermal characteristics of this warm and salty subsurface water mass exhibit a V-shaped multi-decadal variation, in stark contrast to much of the surface ocean, which shows a long-term warming trend alongside with global warming.

(2) We focus on the annual maximum lifetime peak intensity (annual maximum LMI) rather than mean or integrated metrics.

The LMI refers to the lifetime maximum intensity attained by a given TC. The annual maximum LMI (annual maximum LMI) is defined as the highest LMI value observed among all TCs in a given year, serving as an indicator of TC's peak intensity. This metric was chosen because our study aims to examine how the subsurface ocean influences the strongest TCs, particularly through mixing and

upwelling processes that bring subsurface water to the surface, thereby modulating SST. Although the precise wind speed threshold for subsurface entrainment remains uncertain, stronger TCs typically induce deeper mixing, incorporating more subsurface water into the mixed layer, which in turn amplifies its thermal influence. Therefore, the annual maximum LMI, which represents the most intense storm of the year, reflects the upper limit of the subsurface ocean's capacity to sustain TC intensification.

We acknowledge that mean and integrated metrics, such as the intensification rate, PDI or ACE, are valuable for capturing the full spectrum of TC activity, as they account for various factors including storm frequency, duration, and track, which can be more dominated by large-scale atmospheric conditions. Additionally, these quantities include both strong and weak storms. Weak winds generally induce shallow mixing and are therefore more strongly influenced by ocean surface conditions. These effects introduce noise, making it harder to isolate and understand the specific role of subsurface ocean temperatures in TC intensification.

We would like to express our sincere gratitude to the reviewers for their constructive feedback regarding the use of the term "TC activity" in reference to the annual maximum LMI. We acknowledge that this was an oversight on our part, and we apologize for any confusion caused by the imprecision of this term. In line with the reviewer's suggestion, we have carefully revised the manuscript, including the title and relevant sections, to consistently use "annual maximum LMI" rather than the more general term "TC activity". This revision ensures that our terminology more accurately reflects the focus of the study and maintains the clarity needed for readers.

Taken together, our study focuses on the role of the subsurface ocean in modulating the annual maximum LMI of TCs, rather than making broad claims about overall TC activity in the context of global warming. We sincerely thank the reviewers for their constructive feedback, which has been invaluable in improving both the clarity and precision of our work.

2.Mechanism of subsurface water temperature dominating SST evolution during TC-induced mixing.

As the engine of TC intensification, the ocean supplies the enthalpy flux to the atmosphere, which is largely determined by the magnitude of SST. It is well established that the evolving SST, rather than the pre-storm SST, plays a more critical role, due to the substantial SST cooling effect caused by TC-induced mixing and upwelling during a TC's passage. Warm subsurface temperatures can effectively mitigate SST cooling, thereby enhancing TC intensification. As supporting evidence, regions of high ocean heat content within upper 200 m closely aligns with areas where most strong TCs are concentrated in the western North Pacific (Fig. 1 of the manuscript). However, the influence of subsurface thermal conditions on the temporal variation of TCs remains unclear. While we have established a strong linkage between decadal-to-multidecadal variations in annual maximum LMI and the variability of a particular layer of subsurface water in our study region, a mechanistic explanation for this relationship remains lacking.

We would like to particularly thank reviewer 3 for his/her valuable and insightful comments. Inspired by these comments, we developed a simple theoretical model to explain the role of subsurface water in dominating SST variation during the TC-induced mixing process. Based on observations, we identified two distinctive layers within the depth range affected by strong TCs in our study region. The surface mixed layer exhibits a strong linear warming trend, while the subsurface layer---primarily occupied by the warm and salty water mass---shows no significant

long-term trend since 1975. However, it exhibits much greater variability (variance) in heat content. During TC-induced vertical mixing and upwelling, this more variable subsurface water becomes the dominant factor in modulating mixed-layer temperature and, ultimately, SST. Further details of our findings are provided below.

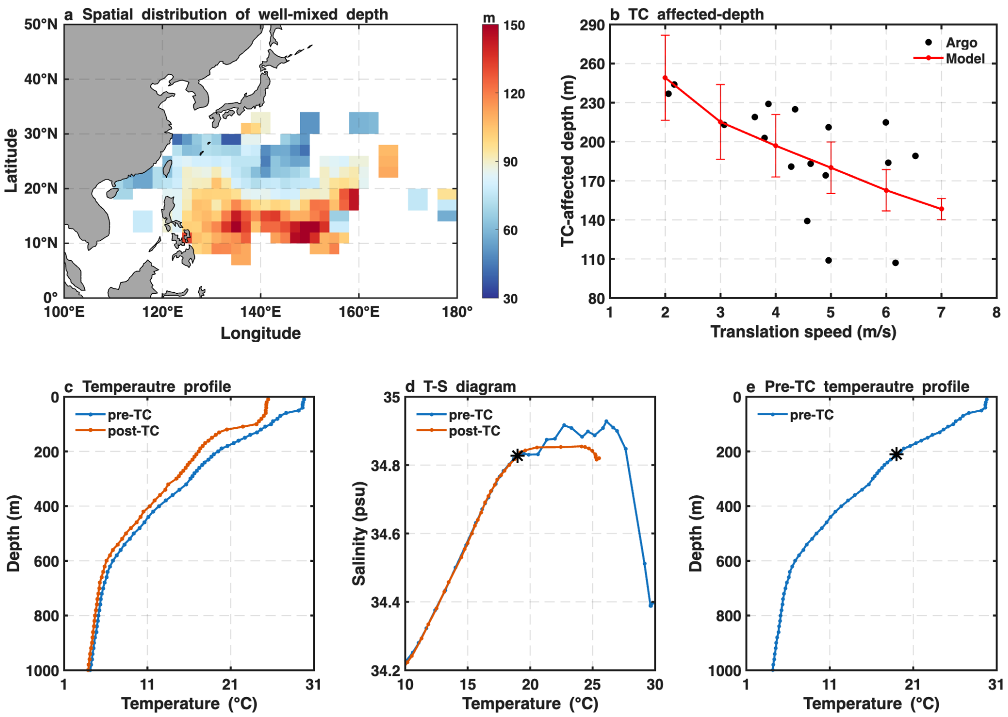

Fig. R1 TC-induced well-mixed depth and TC-affected depth for strong TCs. (a) *Spatial distribution of TC induced well-mixed depth (Methods).* ***(b)*** *TC-affected depth as a function of translation speed (method details shown in panels* ***(c)***–***(e)****). Argo profiles are sampled within 0-7 days after TC passage and within 50 km across TC track, considering only considering only strong TCs (Category 4 and 5). The model simulation is forced with a wind speed of 80 m/s. Red lines show the mean value within 50 km across the track, averaged within 0-7 days after TC passage, with error bars indicating one standard deviation.* ***(c)*** *Comparison of temperature vertical profiles at 8.5 days before (blue) and 1.5 days after (red) the passage of TC Choi-wan in September, 2009.* ***(d)*** *Temperature-Salinity profiles at 8.5 days before (blue) and 1.5 days after (red) the passage of TC Choi-wan. The black star denotes where the two profiles start to diverge.* ***(e)*** *The temperature profile before the passage of TC Choi-wan with the star denoting the TC-affected depth obtained from* ***(d)****.*

First of all, we estimated the depth affected by strong TCs (Category 4 and 5) in our study region using two different approaches. According to the first method, the well-mixed depth H was calculated as the range through which the well-mixing of the pre-storm temperature T yields the observed post-storm SST:

$$SST_{post-storm} = (\int_{-H}^{0} T dz)/H$$

This depth was found to exceed 100 m, reaching approximately 150 m, which corresponds to the central depth of the subsurface water mass (Fig. R2a). In comparison, the well-mixed depth for TCs with lower LMI (Category 3 and 4) is typically around 100 m (Price, 2009), substantially shallower than that of strong TCs. On the other hand, once water parcels undergo turbulent

mixing, both their density and T/S change. Based on this consideration, we compared the T-S profiles at a specific location before and after the passage of a TC (Figs. R1cde). We estimated the TC-affected depth by identifying the maximum depth at which the two curves diverged, indicating perceptible differences in their alignment (Figs.1Rcde). The resulting depth averaged above 150 m and could reach as deep as 230 m (Fig. R1b), which is deeper than the bottom of the subsurface water mass. In comparison to the well-mixed depth, the TC-affected depth is significantly greater, thus representing an upper limit of the depth influenced by TCs. Though they are defined differently, estimates of the well-mixed depth and the TC-affected depth suggest that effects of strong TCs readily extend far beyond the surface mixed layer, entrain much of the subsurface water mass and cause it to mix with waters above.

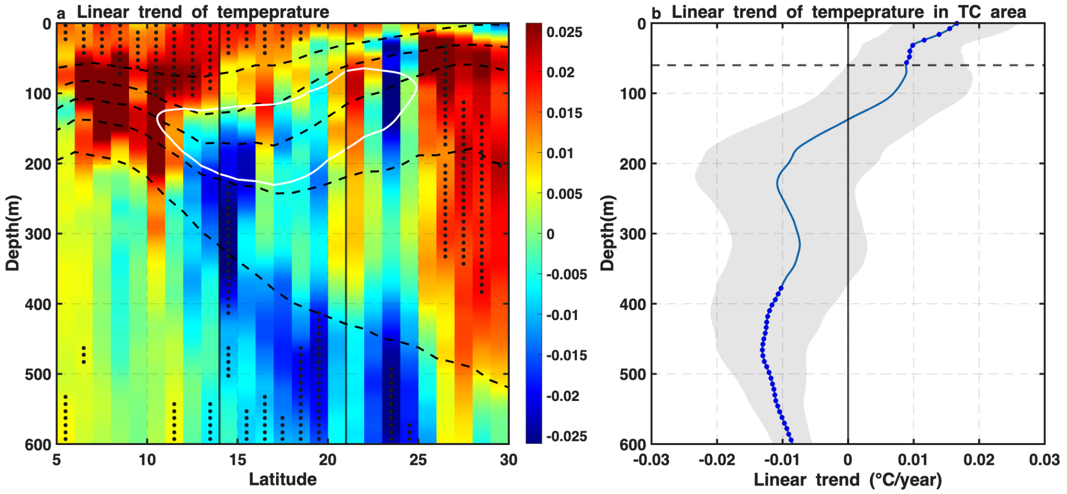

Fig. R2 Linear trends of temperature based on 137°E dataset from 1975 to 2020. (a) *Depth-latitude distribution of the temperature linear trend (shading). The black dashed contours denote isopycnals from 22 to 26 kg/m^3 at intervals of 1 kg/m^3. Waters with salinity > 34.9 psu is bounded by the white contour. Black dots show results that are statistically significant (p<0.05). Black solid lines denote 14°N and 21°N respectively.* ***(b)*** *Linear trend of temperature averaged in 14°-21°N. Blue dots show results that are statistically significant (p<0.05), and the gray shading represents the 95% confidence intervals. The black dashed line denotes 60 m depth.*

Secondly, we calculated linear trends of temperature as a function of depth and latitude along the 137°E meridional section in the western North Pacific (Fig. R2). The surface mixed layer (~60 m deep) exhibits a widespread and significant warming trend over the past four decades. Subsurface temperature also exhibits warming in both tropical and higher-latitude region, except between 14°N -21°N. In this special area, the subsurface layer (from ~60 m to ~350 m) does not display a significant linear trend, particularly within the warm and salty subsurface water mass, as outlined by the white salinity contour of 34.9 psu in Fig. R2a.

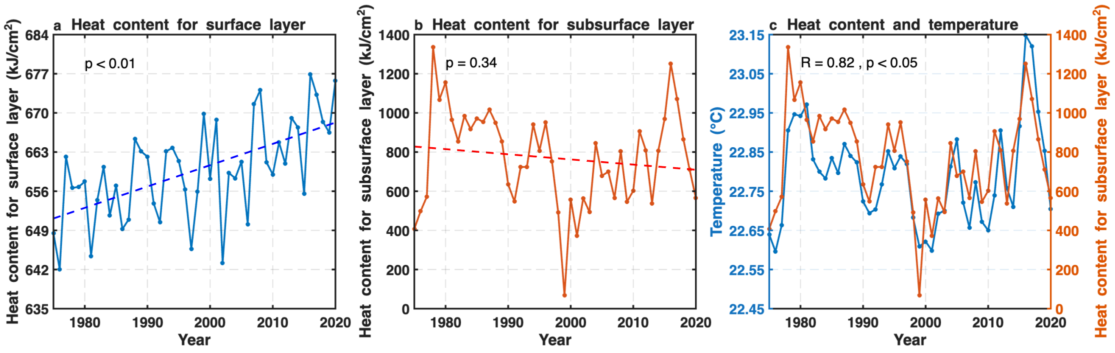

Fig. R3 Heat content in the surface and subsurface layers. (a) *Time series of surface layer heat content.* ***(b)*** *Time series of subsurface layer heat content.* ***(c)*** *Comparison of the temperature along the 24 kg/m³ isopycnal and the subsurface layer heat content. Dashed lines in* ***(a)*** *and* ***(b)*** *show the linear regression with p-value denotes on each panel.*

Based on this two-layer structure, we calculated the heat content of each layer (Figs. R3ab), with the subsurface layer defined as water with salinity greater than 34.9 psu. The correlation between the heat content of these two layers was found to be only -0.06, suggesting their thermal independence. In addition, the heat content of the subsurface salty layer is highly correlated with the temperature along the 24 kg/m³ isopycnal, both following a V-shaped multi-decadal variation pattern (Fig. R3c). This close consistency between heat content and isopycnal temperature indicates the co-variation of temperature and thickness of the warm and salty water mass. To explicitly assess the influence of this particular vertical structure of water column on SST under TC-induced strong mixing, we derived an analytical expression for the correlation between the post-mixing SST and the initial pre-mixing thermal structure of the water column.

The mixed-layer temperature after a TC's passage (T_{mix}) is calculated as:

$$T_{mix} = \frac{\int_{-H_1}^{0} T(z,t)\,dz + \int_{-(H_1+H_2)}^{-H_1} T(z,t)\,dz}{H_1 + H_2} \quad (1)$$

where $T(z,t)$ is the pre-storm temperature profile, H_1 is the thickness of the surface layer, and $H_1 + H_2$ is the well-mixed depth. Given this two-layer structure, the terms $\int_{-H_1}^{0} T(z,t)\,dz$ and $\int_{-(H_1+H_2)}^{-H_1} T(z,t)\,dz$ can be analyzed as integrated variables $C_1(t)$ and $C_2(t)$, representing the time-varying heat content of the surface and the subsurface layers. Then T_{mix} is given as:

$$T_{mix} = \frac{C_1(t) + C_2(t)}{H_0} \quad (2)$$

where H_0 denotes $H_1 + H_2$. The correlations (R) of the post-mixing SST (T_{mix}) with each of the two initial layers' temperature are calculated:

$$\begin{cases} R(T_{mix}, T_1) = \dfrac{Cov(T_{mix}, T_1)}{\sigma T_{mix} \cdot \sigma T_1} \\ R(T_{mix}, T_2) = \dfrac{Cov(T_{mix}, T_2)}{\sigma T_{mix} \cdot \sigma T_2} \end{cases} \quad (3)$$

where T_1, T_2 *are assumed as the mean temperature of the surface and subsurface layer, respectively. Taking the calculation of the correlation with* T_1 *as an example,* $Cov(T_{mix}, T_1)$ *is the covariance of* T_{mix} *and* T_1*, and* $\sigma T_{mix}, \sigma T_1$ *are the standard deviations of* T_{mix} *and* T_1. $Cov(T_{mix}, T_1)$ *can be derived as:*

$$Cov(T_{mix}, T_1) = Cov\left(\frac{C_1 + C_2}{H_0}, T_1\right)$$

$$= \frac{1}{H_0} Cov(C_1, T_1) + \frac{1}{H_0} Cov(C_2, T_1)$$

$$= \frac{1}{H_0} \sigma C_1 \cdot \sigma T_1 \cdot R(C_1, T_1) + \frac{1}{H_0} \sigma C_2 \cdot \sigma T_1 \cdot R(C_2, T_1) \quad (4)$$

σT_{mix} *can be derived as:*

$$\sigma T_{mix}^2 = \left(\frac{\sigma C_1}{H_0} + \frac{\sigma C_2}{H_0}\right)^2$$

$$= \frac{1}{H_0^2} \sigma^2 C_1 + \frac{1}{H_0^2} \sigma^2 C_2 + \frac{2}{H_0} \sigma C_1 \cdot \sigma C_2 \cdot R(C_1, C_2)$$

$$\sigma T_{mix} = \left[\frac{1}{H_0^2} \sigma^2 C_1 + \frac{1}{H_0^2} \sigma^2 C_2 + \frac{2}{H_0} \sigma C_1 \cdot \sigma C_2 \cdot R(C_1, C_2)\right]^{1/2} \quad (5)$$

The correlation of T_{mix} *and* T_1 *can be obtained from equations (3), (4) and (5) as:*

$$R(T_{mix}, T_1) = \frac{\frac{1}{H_0} \sigma C_1 \cdot \sigma T_1 \cdot R(C_1, T_1) + \frac{1}{H_0} \sigma C_2 \cdot \sigma T_1 \cdot R(C_2, T_1)}{\left[\frac{1}{H_0^2} \sigma^2 C_1 + \frac{1}{H_0^2} \sigma^2 C_2 + \frac{2}{H_0} \sigma C_1 \cdot \sigma C_2 \cdot R(C_1, C_2)\right]^{\frac{1}{2}} \cdot \sigma T_1} \quad (6)$$

Since the heat content of the subsurface layer is highly correlated with the core temperature of the water mass but is independent of the mixed-layer temperature, we have $R(C_1, T_1) \approx 1$ *and* $R(C_2, T_1) \approx 0$*, therefore*

$$R(T_{mix}, T_1) \approx \frac{\sigma C_1}{H_0 \left[\frac{1}{H_0^2} \sigma^2 C_1 + \frac{1}{H_0^2} \sigma^2 C_2 + \frac{2}{H_0} \sigma C_1 \cdot \sigma C_2 \cdot R(C_1, C_2)\right]^{\frac{1}{2}}} \quad (7)$$

Similarly, the correlation between T_{mix} *and* T_2 *is given by*

$$R(T_{mix}, T_2) \approx \frac{\sigma C_2}{H_0 \left[\frac{1}{H_0^2} \sigma^2 C_1 + \frac{1}{H_0^2} \sigma^2 C_2 + \frac{2}{H_0} \sigma C_1 \cdot \sigma C_2 \cdot R(C_1, C_2)\right]^{\frac{1}{2}}} \quad (8)$$

The ratio between them is given by:

$$\frac{R(T_{mix}, T_1)}{R(T_{mix}, T_2)} \approx \frac{\sigma C_1}{\sigma C_2} \quad (9)$$

that is, the ratio of the heat content standard deviations for the two layers. Based on previous results, C_1 *is calculated as the heat content for the upper 60 m, and* C_2 *is calculated for the salty water (>34.9 psu) mass above 150 m. While limited in depth, the latter was found to be highly correlated with the heat content of the entire water mass. The variances of the two components are respectively* σC_1 *=8.9,* σC_2 *=186.5. Although they exhibit comparable magnitudes of heat content, the subsurface layer's heat content variability is approximately 20 times greater than that of the surface layer. This dominant variability allows the subsurface layer to control SST evolution, which may ultimately feed back onto the annual maximum LMI.*

Taken together, effects of strong TCs in the study region extend far beyond the surface mixed layer,

causing much of the subsurface warm and salty water mass to mix with surface waters. Due to the greater variability in heat content of the lower layer, the SST resulting from the turbulent mixing is highly correlated with the thermal conditions of the subsurface layer rather than the surface layer.

3、Novelty of our work

We would like to sincerely thank Reviewer 2 for his/her thoughtful and constructive feedback regarding the novelty of our study. We acknowledge that the original version of the manuscript did not fully highlight the unique aspects of our work. In response to this comment, we have revised the manuscript to more clearly emphasize the novel contributions of our study.

(1) Focusing on the annual maximum LMI to highlight subsurface influence

As described in our General Response 1(page 1-2), we investigated the annual maximum LMI rather than mean or integrated properties such as PDI, ACE, mean LMI and mean intensification rate. The novelty lies in our targeted analysis of the most intense TCs, which allows us to isolate and highlight as much as possible the subsurface conditions that may otherwise be obscured in broader studies that aggregate all TC events. While the annual maximum LMI metric may not fully capture overall TC activity, it serves as a more precise indicator of the ocean's maximum capacity to sustain the most intense TC events. This approach provides new insights into how subsurface ocean conditions influence the most powerful storms, a topic that has been less explored in existing research.

(2) Discovery of the V-shaped multi-decadal variation of the annual maximum LMI.

Our study focuses on a critical region in the western North Pacific (120°-150°E, 14°-21°N), known for its high frequency and intensity of strong TCs. This region is underlain by a warm, salty subsurface water mass, which provides a favorable thermal background for development of intense TCs. More importantly, we have identified a novel and unexpected multi-decadal variation in the behavior of the annual maximum LMI, which closely mirrors changes in the subsurface warm and salty water mass.

This finding is somewhat surprising because it challenges the expectation that global warming will result in intensification of TCs (Stowasser et al., 2007; Mei et al., 2015). Instead, the observed V-shaped pattern suggests that the annual maximum LMI is more closely tied to a particular subsurface layer than to the surface temperatures or to the upper ocean overall heat content such as T80 in Lin et al. 2013 or T105 in Jin et al. 2014. This contrasts with studies of mean or integrated metrics (such as mean intensification rate) that reflect properties of not only strong TCs but weak ones. Weak TCs are more affected by ocean surface conditions, so mean or integrated metrics exhibit similar increasing trend to global warming (Kang et al. 2024).

(3) Discovery of the mechanism underlying the V-shaped multi-decadal variation of the annual maximum LMI.

While the presence of the warm and salty subsurface water mass is essential for creating favorable conditions for strong TCs, this background mean state alone does not explain the observed V-shaped variation in annual maximum LMI. Our further analysis (as described in detail in General Response 2 (page 2-6), reveals two distinctive thermal characteristics of this subsurface layer that collectively explain its decisive role. First, the subsurface water mass shows no statistically significant warming trend, in contrast to and independent of the broad and pronounced surface warming. Second, it possesses substantially greater variability in heat content than the surface layer, despite comparable magnitudes. Based on a theoretical derivation describing the relation between the after-mixing SST and the pre-mixing thermal structure of the water column, we found the

subsurface water mass dominates the evolution of after-mixing SST, thereby regulating the annual maximum LMI of TCs.

(4) Discovery of direct linkage between the annual maximum LMI in the western North Pacific and the remote surface condition in the eastern North Pacific.

We would like to sincerely thank Reviewer 1 for his/her valuable comment regarding the direct linkage between the TC property and the ocean surface conditions in the subduction region of the warm and salty water mass. Following the reviewer's suggestion, we directly connect the annual maximum LMI to the remote surface conditions. By tracking the subsurface temperature signal, we identify a pathway originating from the subtropical eastern North Pacific (Fig. R4a). The warm and salty water mass forms and is subducted beneath the North Pacific High (NPH), a large-scale pressure system centered near (220°E, 35°N) in the climatological mean. A 4.5-year lag is observed between the NPH and the core temperature of the subsurface water mass in the TC region under 1-year smoothing (Fig. R4b), demonstrating the role of the oceanic subsurface passage in signal transmission. A direct comparison shows a strong relationship between the annual maximum LMI and the NPH intensity, with the NPH leading TC intensity by approximately 5.5 years under 3-year smoothing (Fig. R4c). While the discrepancy in these time lags estimates may result from the smoothing procedure, the overall lead-lag relationship remains robust.

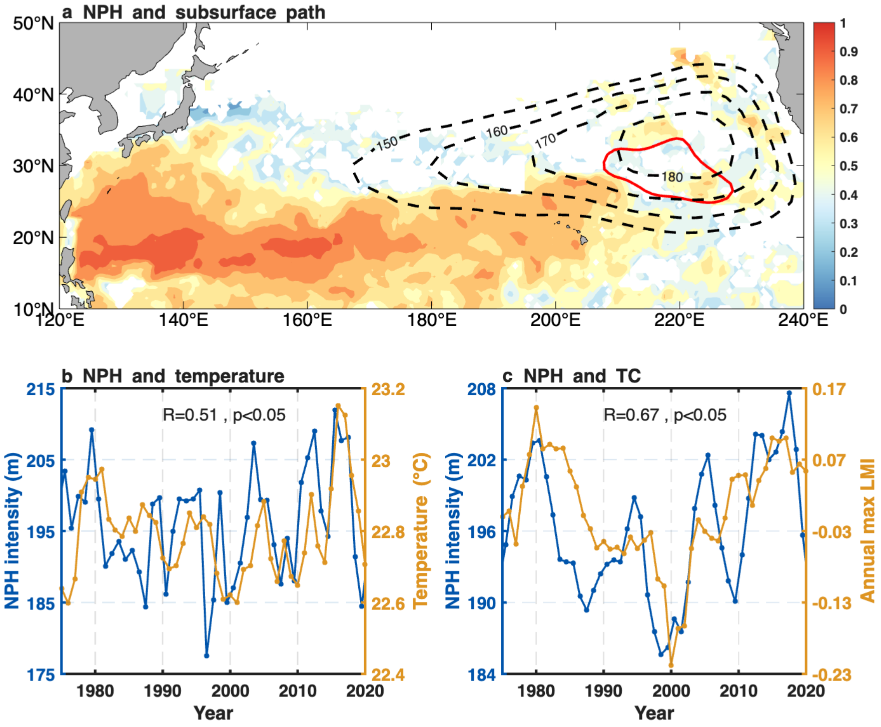

Fig. R4 The relation among the North Pacific High (NPH), annual maximum LMI and the subsurface water temperature. (a) *Spatial distribution of the NPH and the subsurface water mass transport path. The shading denotes the correlations between the temperature within the TC active area (134°-140°E, 14°-21°N, as the same showed in Fig. 3c of the manuscript) and that of all other regions along the 24 kg/m*^{3} *isopycnal. The dashed contours represent the climatology NPH spatial distribution. The red contour marks areas in the eastern North Pacific where the mixed layer depth in February exceeds 120 m.* ***(b)*** *Time series of NPH intensity (blue) and subsurface temperature along the 24 kg/m*^{3} *isopycnal (orange) based on 137°E dataset during 1975-2020. The line of NPH intensity was shifted to*

the right for 4.5 years. A 1-year smoothing was applied to both time series. ***(c)*** *Time series of NPH intensity (blue) and annual maximum LMI (orange). The annual maximum LMI time series is averaged by four agencies (USA, HKO, CMA and JMA). The NPH intensity is calculated as the average of the ten largest values of geopotential height at 1000 dbar. The line of NPH intensity was shifted to the right for 5.5 years. A 3-year smoothing was applied to both time series.*

In summary, our findings reveal a coherent predictive chain linking remote atmospheric forcing to annual maximum LMI. Water masses formed under the NPH effectively preserve atmospheric signals and are transported conservatively via subsurface pathways. The subsequent V-shaped variation in annual maximum LMI thus appears to be a delayed response to these earlier, remotely established atmospheric and oceanic conditions, given the fact that variation of NPH is probably a result of air-sea interaction process. This mechanism illustrates how large-scale atmospheric/oceanic variability is stored in the ocean and subsequently influences TC intensity years later.

Reference:
Lin, I. I. et al. An ocean coupling potential intensity index for tropical cyclones. Geophys. Res. Lett. ***40****, 1878–1882 (2013).*

Kang, S. K. et al. The North Equatorial Current and rapid intensification of super typhoons. Nat. Commun. ***15****, 1742 (2024).*

Balaguru, K., Leung, L. R., & Yoon, J. H. Oceanic control of northeast Pacific hurricane activity at interannual timescales. Environ. Res. Lett. ***8****, 044009 (2013).*

Jin, F. F., Boucharel, J., & Lin, I. I. Eastern Pacific tropical cyclones intensified by El Niño delivery of subsurface ocean heat. Nature ***516****, 82-85 (2014).*

Price, J. F. Metrics of hurricane-ocean interaction: vertically-integrated or vertically-averaged ocean temperature?. Ocean Sci. 5, 351–368 (2009).

Stowasser, M., Wang, Y., & Hamilton, K. Tropical cyclone changes in the western North Pacific in a global warming scenario. J. Clim. ***20****, 2378-2396 (2007).*

Mei, W., Xie, S. P., Primeau, F., McWilliams, J. C., & Pasquero, C. Northwestern Pacific typhoon intensity controlled by changes in ocean temperatures. Sci. Adv. ***1****, e1500014 (2015).*

Reviewer #1

1. In this study, Ni et al. show using a combination of observations and numerical experiments that the intensity of TCs in the Western Pacific is primarily controlled by sub-surface water temperatures. Further they also show that these waters are subducted from the Eastern Pacific 3-4 years earlier. The first half of this work is devoted to showing the relevance of sub-surface ocean temperature for TC intensification, which I believe has been demonstrated already in previous studies and is not surprising. However, the second half of the work, where they show the link between sub-surface waters in the TC region and the subduction process is more interesting and novel. Also, it has practical implications as it is related to predictability. If the authors can show that surface ocean conditions in the subduction region of Eastern Pacific directly controls TC activity in the Western Pacific at lead times of 3-4 years, it'll make the study much more impactful. Other comments and suggestions for improving the study are provided below.

Response: We would like to express our sincerest gratitude to you for drawing our attention to the linkage between the surface ocean conditions in the subduction region and the TC activity directly, which has greatly enriched our understanding of the physical processes.

To identify the source of the subsurface water mass, we calculated the correlation between the temperature within our study area (134°-140°E, 14°-21°N) and that of all other regions along the 24 kg/m³ isopycnal. This isopycnal surface, as demonstrated in Fig. 3, is at the core of the subsurface water mass. The result clearly reveals that the subsurface transport pathway originates from the subtropical outcrop region in the eastern North Pacific (Fig. R4a). This region, which also coincides with the area where the climatological mixed layer depth (MLD) in late winter (February) exceeds 120 m (red contour in Fig. R4a), is characterized by deep mixing and is a known formation zone of the North Pacific Eastern Subtropical Mode Water (NPESTMW) (Suga et al., 2004; Katsura et al., 2013; Guo et al., 2018). Moreover, as we turn our attention to the atmosphere, the formation site is under the center of the North Pacific High (NPH) (Schwing et al., 2002), a large-scale pressure system in the eastern Pacific Ocean with the climatological mean position around (220°E, 35°N). Thus, the source region of this subsurface water mass is characterized by a two-fold coincidence: it is simultaneously a mode-water formation area and an atmospheric high-pressure center. This convergence implies a close dynamical linkage with surface oceanic and atmospheric conditions in the formation and subduction of the NPESTMW.

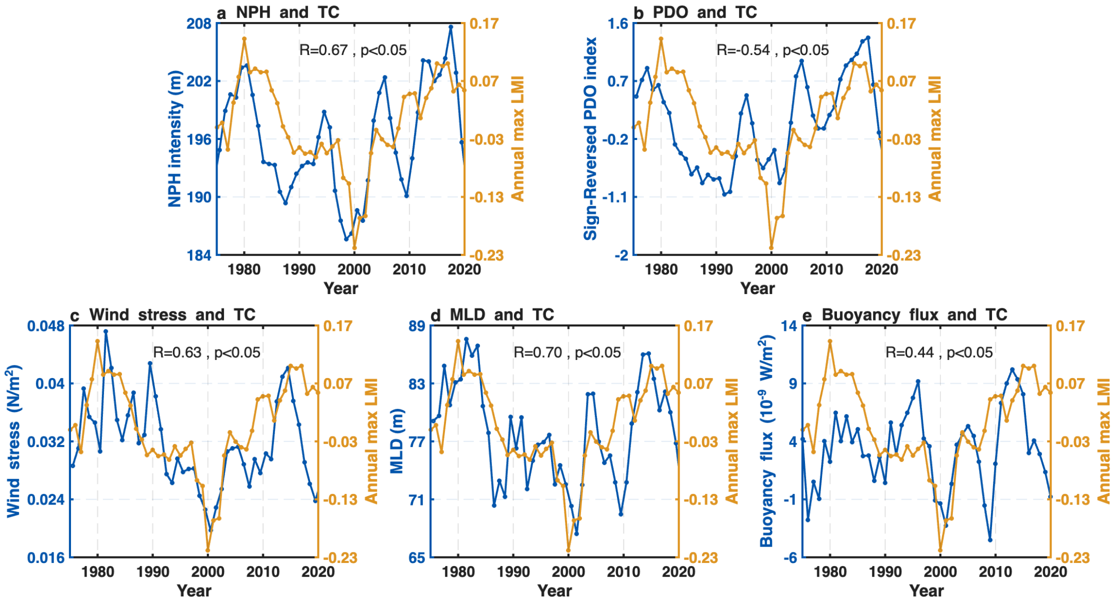

Fig. R5 Relationship between the annual maximum lifetime maximum intensity (LMI) of TCs and various variables potentially significant for formation and subduction of the water mass. *Time series of annual maximum LMI and* ***(a)*** *North Pacific High (NPH) intensity;* ***(b)*** *Pacific Decadal Oscillation (PDO) index;* ***(c)*** *March wind stress averaged over the water mass formation region;* ***(d)*** *January-March mean mixed layer depth (MLD) averaged over the formation region;****(e)*** *January-March mean buoyancy flux averaged over the formation region. The LMI time series is averaged by four agencies (USA, HKO, CMA and JMA). A 3-year smoothing was applied to time series. The water mass formation region is defined as 200°-240°E, 20°-35°N. In each panel, the time series of the variable (blue) is shifted to the right by 5.5 years, and the corresponding lead correlation coefficient is indicated.*

Previous studies indicate that decadal variations in the volume of NPESTMW originate from Pacific Decadal Oscillation (PDO)-induced anomalies in air-sea buoyancy flux. These buoyancy flux anomalies drive decadal changes in mixed layer depth, leading to anomalous subduction (Guo et al., 2018). Concurrently, sea level pressure (SLP) is another key factor in NPESTMW formation (Wang et al., 2024). SLP variability modulates large-scale wind patterns, which subsequently affect latent heat flux and thereby the subduction rate of mode water. As the dominant SLP system in this region, the NPH likely influences water mass formation through a similar atmospheric pathway.

We systematically compared the relationships between annual maximum LMI of TCs and several key surface variables. These include wintertime wind stress, mixed layer depth, and buoyancy flux averaged over the water mass formation region (200°-240°E, 20°-35°N), as well as two large-scale climate indices, the NPH intensity and the PDO index (Fig. R5). Under a 3-year smoothing, all examined variables lead annual maximum LMI by approximately 5.5 years. Among them, the NPH, wind stress, and mixed layer depth show the strongest correlations with the annual maximum LMI, implicating them as primary yet closely related influences on the formation and characteristics of the source water mass. As indicated by our result in Fig. 3d (based on 1-year smoothing), it takes the subsurface water mass about 3-4 years translating from the subduction region to the TC region, which is significantly shorter than the 5.5-year lag time suggested by Fig. R5.

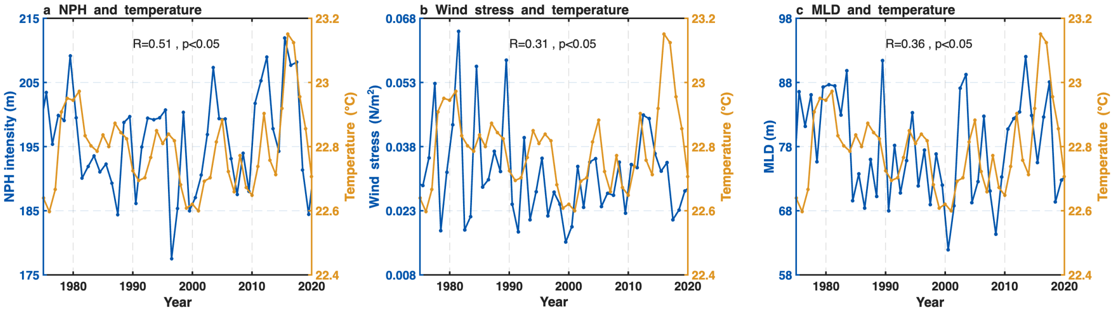

Fig. R6 Relationship between the subsurface temperature and various variables. *Time series of subsurface temperature along the 24 kg/m^3 isopycnal in the TC region (120°-150°E, 14°-21°N) and (**a**) North Pacific High (NPH) intensity; (**b**) March wind stress averaged over the water mass formation region; (**c**) January-March mean mixed layer depth (MLD) averaged over the formation region. A 1-year smoothing was applied to time series. In each panel, the time series of the influence variable (blue) is shifted to the right by 4.5 years, and the corresponding lead correlation coefficient is indicated.*

To explain this discrepancy, we compared various variables with the temperature along the 24 kg/m3 isopycnal in the TC region using a 1-year smoothing (Fig. R6). The NPH showed the most significant correlation, leading the temperature variation by 4.5 years. Over the entire period (1975-2020), the correlation coefficient between the two is 0.51. After 2000, their lead-lag correlation increased markedly over the second half period, reaching 0.70. Therefore, the discrepancy in these time lag estimates likely stems from the smoothing procedure. Nevertheless, the overall lead-lag relationship remains robust regardless of the smoothing period.

This warm and salty water mass exists a lag between its formation and the initiation of its westward transport. Its initial formation is preconditioned by the previous winter's mixed layer dynamics under surface forcing. As a weak seasonal thermocline develops and the mixed layer shoals in late spring, the water mass is isolated in the subsurface (Marshall et al., 1993; Guo et al., 2018). Therefore, these waters are subsequently retained for a few months in the formation area, undergoing processes like ventilating and evaporating, before being subducted into the thermocline and ultimately released. That said, the 4.5-year lag between NPH and the water mass core temperature in the TC region is reasonable.

In summary, our findings illustrate a coherent chain of influence. In the source region, water masses formed under the NPH effectively preserve atmospheric signals (Fig. R4-R6) and are subsequently transported quasi-conservatively through subsurface pathways. Over the past four decade, changes of NPH consist of quasi-decadal oscillations superimposed on a striking multi-decadal V-shaped structure. The observed V-shaped variation of the annual maximum LMI is therefore likely a consequence of the earlier changes of dynamical and thermodynamical factors such as NPH in the eastern North Pacific.

Once again, we are grateful for your insightful suggestion, which was instrumental in uncovering the direct linkage between the annual maximum LMI and the NPH. This key finding is a novel and impactful contribution of our study (please refer to General Response 3 (page 7-8) for a discussion about the novel features of the study). A new paragraph has been added in Lines 261-319 of our revised manuscript to discuss this key finding.

2. Lines 76-80: I'm not sure this statement is entirely accurate. Previous studies have indeed demonstrated the significance of the ocean sub-surface thermal structure for Eastern Pacific TCs (Balaguru et al., 2013; Jin, F-F. et al., 2014).

Response: We thank you for pointing out our oversight regarding previous research. We agree that Balaguru et al. 2013, Jin et al. 2014 and Lin et al. 2013 have demonstrated the significance of subsurface water temperatures to TC intensification. However, as described in our General Response 1(page 1-2), to understand the evolution of the most intense TCs and its dependence on the subsurface ocean, our study focuses on the annual maximum LMI, rather than other integrated or mean metrics of TC such as the LMI for all TCs in Lin et al. 2013, ACE in Jin et al. 2014, and PDI in Balaguru et al. 2013. The annual maximum LMI reflects the upper limit of the subsurface ocean's capacity to sustain TC intensification. Mean or integrated metrics are valuable for characterizing the average TC activity, as they account for various factors including storm frequency, duration, and track, but these metrics cannot isolate the subsurface ocean's thermal influence, as they are more influenced by large-scale atmospheric conditions. Additionally, the mean or integrated metrics include lower wind speeds, which are more strongly influenced by ocean surface conditions. These surface effects introduce noise, making it harder to isolate and understand the specific role of subsurface ocean temperatures in TC intensification.

Through our investigation in the key TC region in the western North Pacific, we found the interannual-to-multidecadal variation of the annual maximum LMI is much more closely consistent with that of thermal properties of a particular subsurface layer, than evolution of surface temperatures or evolution of the overall heat content of the upper ocean as T80 in Lin et al. 2013 or T105 in Jin et al. 2014. In accordance with your recommendation, we have revised the Introduction section in Lines 63-79 and Discussion section in Lines 440-442 of the revised manuscript.

3. Fig. 1a: Do you include all TC track locations in this? If that's the case, I would suggest trying with only those track locations where the wind speeds are higher. I'm not sure locations where the TC winds are weak (eg. Tropical Storm, Cat 1-2) the storms can mix down to a depth of ~200 m.

Response: We thank you for pointing out the need for clarification. We agree that weaker TC winds are unlikely to induce mixing to depths of ~200 m. Therefore, as you reasonably inferred, Fig. 1a does not display all TC tracks, but only those of TCs with LMI greater than 70 m/s since 2010. The figure caption has been revised to clearly state (Fig. R7) in Lines 146-149 of the revised manuscript: " Tracks of super TCs since year 2010, superimposed on the mean temperature for the upper 200 m based on Argo data (shade). The Black lines represent TC tracks with LMI exceeding 70 m/s. Black dots overlaid on the tracks indicate locations where the wind speed exceeds 70 m/s."

4. The results presented in Fig. 2a and 2b are consistent with Lin, I-I et al. (2013), who showed the importance of the sub-surface ocean temperature in controlling the LMI of TCs in the western Pacific.

Response: We thank you for highlighting this important connection. We agree that Lin et al. 2013 demonstrated the importance of subsurface ocean temperature in controlling the LMI of TCs in the western North Pacific, which indeed provides an important foundation for our work. However, their analysis considered the LMI of all TCs and examined its relationship with upper-ocean heat content (e.g., T80), an approach that may also reflect the influence of surface conditions. In contrast, our

study focuses specifically on the annual maximum LMI, which emphasizes the most intense events each year. By analyzing the vertical structure of ocean temperature, we find that in our study region, the annual maximum LMI is most strongly linked to subsurface water masses rather than surface or the upper-layer heat content.

5. In Figs. 1b, 2a, 2b and 2d, please indicate uncertainty bounds. Also, the statistical significance of correlation coefficients must be indicated in Fig. 2c.

Response: We thank you for this comment. We have indicated uncertainty bounds in Figs. 1b,2a,2b and 2d. In Fig. R8a (Fig. 2a of the manuscript), we use the results from the USA dataset to exemplify the uncertainty bounds (gray bars) for clarity. The complete set of results from all four agencies (USA, HKO, JMA, and CMA) is presented respectively in Fig. R9 to facilitate a detailed comparison.

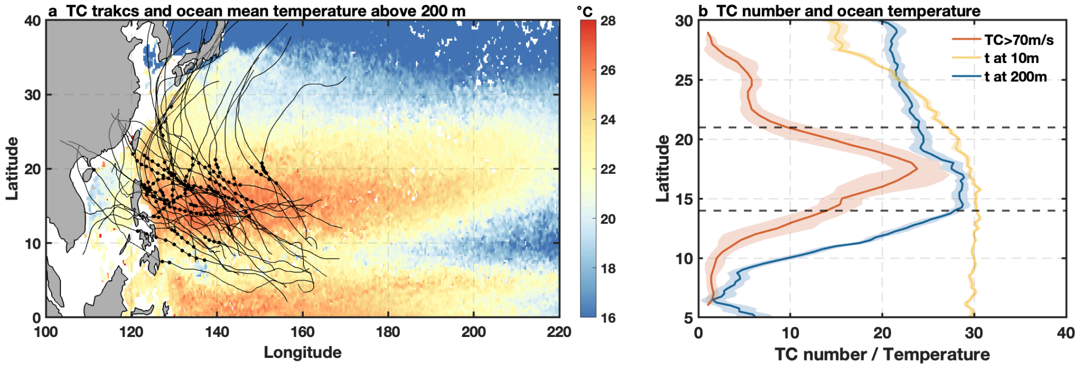

Fig. R7 Super TCs and ocean water temperature. *(**a**) Tracks of super TCs since year 2010, superimposed on the mean temperature for the upper 200 m based on Argo data (shade). The Black lines represent TC tracks with LMI exceeding 70 m/s. Black dots overlaid on the tracks indicate locations where the wind speed exceeds 70 m/s. (**b**) Meridional distribution of the accumulated occurrence (red line, in 6 hours) of super TCs for 1975-2020 and ocean surface (yellow line) and subsurface (blue line) temperature. The shading denotes the standard deviation. Temperatures shown are normalized values (Methods). TC data used in (**a**) and (**b**) are from IBTrACS-USA.*

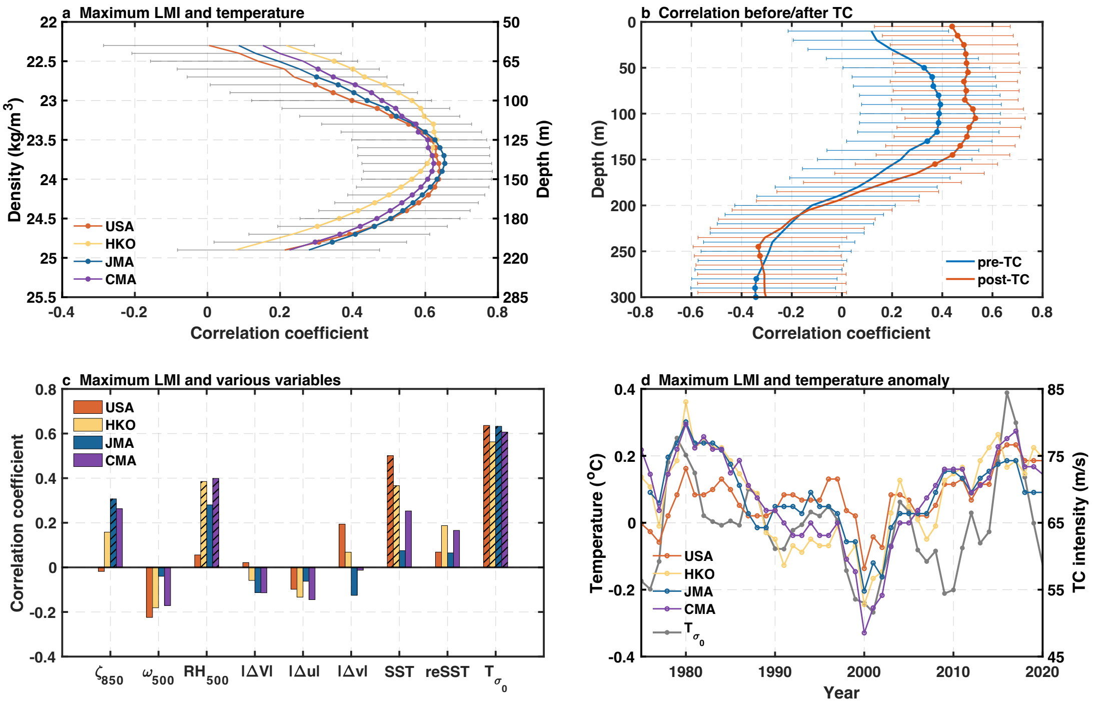

Fig. R8 Correlation between TC intensity and various oceanic and atmospheric variables. (a) *Simultaneous correlations between the annual maximum LMI and the temperature through the water column along the 137°E meridian. Color dots show results statistically significant (p<0.05), and the gray bars show the 95% confidence intervals of analysis based on USA. (**b**) Correlations between TC intensity (LMI>70m/s) and the local temperature at different depths before (blue) and after (red) TC passage based on Argo data. Color dots show results statistically significant (p<0.05) with blue and red bars show the 95% credible intervals. (**c**) Simultaneous correlation between the annual maximum LMI and various atmospheric and ocean variables.* ζ_{850}*, vorticity at 850 hpa;* ω_{500}*, omega* (dp/dt) *at 500 hpa, p is the pressure;* RH_{500}*, relative humidity at 500 hpa;* $|\Delta V|$*,full shear of 200-850 hpa;* $|\Delta u|$*, shear of zonal wind of 200-850 hpa;* $|\Delta v|$*, shear of meridional wind of 200-850hpa; SST, sea surface temperature over the TC region; Relative SST, SST over the TC region relative to North Pacific tropical mean SST;* T_{σ_0}*, ocean temperature along isopycnal 24 kg/m*^{3}*. Data used are from summer months (July-September). Black slashes indicate correlation coefficients that are statistically significant (p<0.05). (**d**) Time series of the maximum LMI and temperature anomaly along the isopycnal 24 kg/m*^{3}*. TC data used in (**b**) are from IBTrACS-USA. A 3-year running mean smoothing was applied to all time series.*

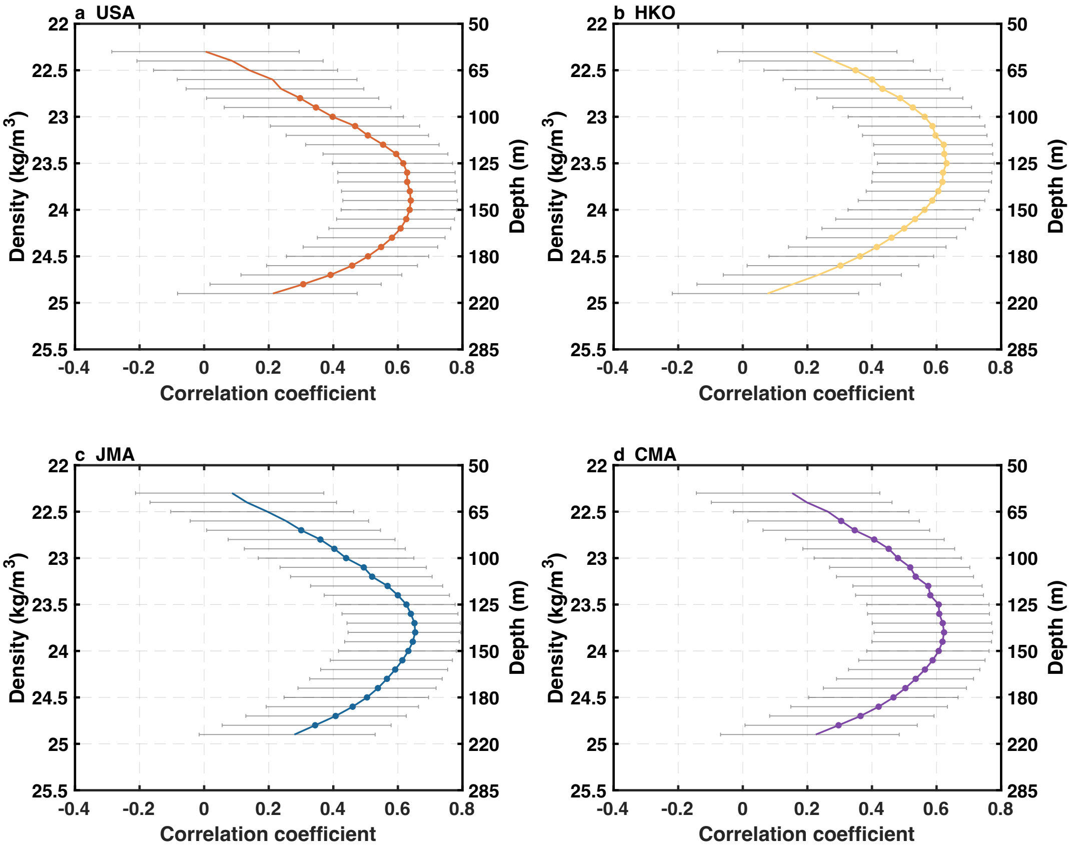

Fig. R9 Correlation between TC intensity and ocean subsurface temperature across four agencies. *(**a**) Simultaneous correlations between the annual maximum LMI and the temperature through the water column along the 137°E meridian. Red dots show results that are statistically significant ($p<0.05$). Gray bars show the 95% credible intervals of analysis based on USA. (**b**)-(**d**) Same as (**a**), but for HKO, JMA and CMA respectively.*

6. Also, in Fig. 2c, I'd replace SLP with mid-level relative humidity. While the role of atmospheric moisture in modulating TC intensity is well-known, it is unclear to me how SLP affects TCs (unless we're talking about SLP in the context of subtropical high pressure and steering flow).

Response: Following your recommendation, we have replaced SLP with mid-level relative humidity in our analysis, with statistical significance of the correlation coefficients indicated by black slashes. For HKO and CMA datasets, the correlation between the annual maximum LMI and relative humidity is weak despite passing the significance test. For the USA and JMA datasets, the correlation is not statistically significant ($p>0.05$). This inconsistency indicates that the relationship between relative humidity and TC intensity is not robust across the four datasets. In contrast, subsurface temperature shows consistently significant correlations with the annual maximum LMI from all four agencies, demonstrating a more stable and physically coherent linkage. We also have replaced Fig.2 with this Fig.R8 in the revised manuscript.

7. Lines 292-301: This analysis is quite interesting, as it relates to predictability. While the authors

show that the sub-surface conditions in the TC region are correlated to surface conditions in the subduction region with a lead time of 3-4 years, can they extend this to TCs directly? In other words, if they use a metric like ACE in the TC region, how well is this correlated with surface conditions in the subduction region 3-4 years prior?

Response: please refer to our response to your general comments.

8. Lines 347-349: While mixing is always important, upwelling typically plays a role only in slow-moving TCs.

Response: We would like to thank you for pointing out the imprecision in our statement. In response to this valuable feedback, we have rephrased the sentence to enhance clarity in Lines 372-374 of the revised manuscript.

Reference:

Suga, T., Motoki, K., Aoki, Y., & Macdonald, A. M. The North Pacific climatology of winter mixed layer and mode waters. J. Phys. Oceanogr. ***34****, 3-22(2004).*

Katsura, S., Oka, E., Qiu, B., & Schneider, N. Formation and subduction of North Pacific tropical water and their interannual variability. J. Phys. Oceanogr. ***43****, 2400-2415 (2013).*

Guo, Y., Lin, X., Wei, M., Liu, C., & Men, G. Decadal variability of North Pacific eastern subtropical mode water. J. Geophys. Res.: Oceans ***123****, 6189-6206 (2018).*

Schwing, F. B., Murphree, T., & Green, P. M. The Northern Oscillation Index (NOI): a new climate index for the northeast Pacific. Prog. Oceanogr. ***53****, 115-139 (2002).*

Wang, J., Liu, L., Lin, Y., Yu, J., & Wang, F. A see-saw of subduction rate in the North Pacific western and eastern subtropical mode water formation areas induced by the Aleutian Low. Geophys. Res. Lett. ***51****, e2024GL110837 (2024).*

Marshall, J. C., Williams, R. G., & Nurser, A. J. G. Inferring the subduction rate and period over the North Atlantic. J. Phys. Oceanogr. ***23****, 1315-1329 (1993).*

Balaguru, K., Leung, L. R., & Yoon, J. H. Oceanic control of northeast Pacific hurricane activity at interannual timescales. Environ. Res. Lett. ***8****, 044009 (2013).*

Lin, I. I. et al. An ocean coupling potential intensity index for tropical cyclones. Geophys. Res. Lett. ***40****, 1878-1882 (2013).*

Jin, F. F., Boucharel, J., & Lin, I. I. Eastern Pacific tropical cyclones intensified by El Niño delivery of subsurface ocean heat. Nature ***516****, 82-85 (2014).*

Reviewer#2

This manuscript seeks to understand oceanic controls behind the annual lifetime maximum intensity (aLMI) of TCs in the western north Pacific Ocean. To do so, the authors first show that the empirical frequency of TCs with wind speed > 70 m/s is greatest at the latitude (typhoon alley) where both the ocean surface and subsurface are above 26 degrees Celsius (Fig. 1b). As further confirmation, the authors compute correlations between many variables and aLMI in the region, and find that ocean temperature along the ~1024 kg/m3 isopycnal holds the greatest correlation (Fig. 2c).

After establishing that subsurface, and not surface, ocean temperatures hold the strongest statistical correlation with aLMI, the authors dedicate Figs. 3-5 to:

i) understanding how the ventilation of subtropical waters regulates subsurface temperature in typhoon alley

ii) how upwelling and turbulent mixing act together to allow the entrainment of deep water temperatures onto the surface mixed layer.

I will center my main comments around three points that caught my attention in the Discussion section.

1、*Lines 356-357: The annual peak intensity of TC in the western North Pacific is exclusively highly correlated with the temperature of a subsurface warm and saline water mass [...]*

At surface level, this is an interesting result. However, once I think about it carefully, I struggle to see how this is surprising or how it merits publication in a prestigious journal. The well-known results in Lin et al (2013, GRL) already established that the true limiting factor for enthalpy fluxes to a TC is not set by the pre-TC SST, but rather by the likely evolution of SST under the TC. If this result is not obvious from the existing literature, the authors need to do a better job at explaining how this is surprising. The focus on this particular area of the north Pacific is valuable, but it was already addressed in this journal and on a similar fashion by Kang et al (2024, Nat. Comms.)

Response: We thank you for raising the important point regarding the foundational work of Lin et al. (2013) and the recent study by Kang et al. (2024). Lin et al. 2013 focused on the LMI for all TCs and found it to be more closely associated with the pre-storm upper ocean heat content T80 instead of the pre-storm surface temperature. Kang et al. 2024 demonstrated that the mean intensification rate of all TCs has shown an increasing trend over the past four decades, which is consistent with the observed warming trend in the upper ocean (T26). Both studies highlight the average TC activity --- whether measured by LMI for all TCs or intensification rate---is strongly influenced by thermal conditions both at and below the ocean surface. While weak winds are more affected by thermal conditions near the ocean surface, strong winds can penetrate deeper into the water column, making them more influenced by subsurface conditions.

Building upon these important findings, our work aims to make a unique contribution by specifically examining the long-term evolution of the most intense TCs, which interact with deeper oceanic layers (please refer to our General Response 1(page 1-2) for a detailed discussion of our primary objective and approach). With this goal in mind, we focus on the behavior of the annual maximum LMI in the western North Pacific where the strongest TCs occur mostly frequently. The annual maximum LMI measures the highest LMI value observed among all TCs in a given year and is a good indicator of the upper limit of the subsurface ocean's capacity to sustain TC intensification.

This choice of TC metric and the study region, grounded in the foundational work of Lin et al.

2013 regarding the importance of the SST evolution under the TC, allows us to directly link the unique warm and salty subsurface water mass to the most powerful storms. This particular metric reveals a V-shaped pattern of multi-decadal variation, without a long-term increasing trend, which contrasts distinctively with the variation of the mean intensification rate observed by Kang et al. 2024 in a nearby region. Furthermore, since the properties of this water mass can be traced back to remote oceanic regions with corresponding atmospheric signals, it presents a potential predictor for TC intensity. Please refer to our General Response 3 (page 7-8) for a detail discussion about the novel aspect of our study. In accordance with your recommendation, the related discussion has been incorporated into the Introduction section in Lines 63-91 and Discussion section in Lines 454-461 of the revised manuscript to articulate the novelty of this study in relation to previous work.

2、*Lines 366-368: [...] the strength of super TCs in the western North Pacific is unlikely to increase in the next few decades.*

How exactly is this claim supported by the text? I have many objections. First, subsurface ocean temperatures are warming in much of the global oceans. Second, whether or not the strength of super TCs changes is not only determined by trends in aLMI. One could envision, for example, an increase in the number of TCs with wind speed > 70 m/s, and an increase in the duration over which this intensity is maintained, and still not see a change in aLMI. In this case, the strength of super TCs would change in ways that are profoundly consequential. Overall, this is a big claim about a highly contested question that, in my opinion, was not granted fair attention and care in the main text.

Response:

We thank you for your valuable comments. First of all, we agree that subsurface ocean temperatures are warming in much of the global oceans, but not in our study region. Fig. R2 shows the warming trend of water temperatures in the western North Pacific in recent decades, we would like to highlight that within our region of interest (14°-21°N), the subsurface layer does not exhibit a pronounced trend. Instead, it shows a multi-decadal V-shaped variation pattern. Moreover, its variability is considerably greater than that of the surface layer. This enhanced variability allows the subsurface to dominantly control the year-to-year changes in the annual maximum LMI in this area. For further details, please refer to our General Response 2 (page 2-6), where we provide a mechanistic explanation. And following your suggestion, we have added a discussion of the mechanism in Lines 398-424 of the revised manuscript.

Secondly, we apologize for the imprecise use of terminology in our original manuscript. We recognize that the annual maximum LMI alone cannot fully represent overall super TC activity, as metrics such as the number and duration of super TCs also significantly influence collective intensity. Therefore, we have revised the title and relevant text throughout the manuscript to remove any implication that the annual maximum LMI directly represents the overall activity of super TCs. Our focus, instead, is specifically on the ocean's influence on the maximum intensity reached by storms in this region.

3、*Lines 372-376: Subsurface temperature thus exerts an important impact on the evolution of the passing-by TCs, whereas the influence of pre-TC is effectively suppressed. Such mechanism explains the weak relation between global warming, but it does not mean the global warming have no impact on TCs.*

This is an interesting excerpt. The first half is not a result of this manuscript, but a well- established

fact for many decades. The second half argues that the crucial role of subsurface temperatures means that the intensity of super TCs is not modified by oceanic temperatures. This seems to imply that there are no temperature trends in the subsurface warm pool, which was not shown in the main text. Again, the authors are making big claims about a highly debated question without having produced robust tests.

Response: We thank you for the valuable comments. As stated in the response to your second comment, the subsurface ocean in our study region does not show a long-term warming trend, but instead shows a V-shaped variation pattern that closely aligns with the pattern observed for the annual maximum LMI. Based on this result and a theoretical explanation of the underlying mechanism, we emphasize the crucial role of the subsurface ocean in modulating the amplitude of peak winds of strong TCs.

This is not to imply that global warming is unimportant. In fact, it likely plays a significant role, especially for weaker storms with shallower impact depth, or in regions where subsurface ocean warming occurs alongside surface warming. For clarity, a related discussion has been added in the Discussion section in Lines 464-474 of the revised manuscript.

Reference:

Lin, I. I. et al. An ocean coupling potential intensity index for tropical cyclones. Geophys. Res. Lett. ***40****, 1878–1882 (2013).*

Kang, S. K. et al. The North Equatorial Current and rapid intensification of super typhoons. Nat. Commun. ***15****, 1742 (2024).*

Reviewer#3

The results are interesting and potentially important for predicting the occurrence of strong typhoons in the western Pacific, if ocean conditions in the northeastern Pacific do lead those in the TC region by several months and subsurface temperature has a strong impact on TC-induced ocean cooling, TC intensity, and LMI. However, the manuscript does not conclusively show either of these. Much of the main analysis is based on correlations (e.g., covariability of subsurface temperature with LMI) without in-depth physical reasoning. The mechanism of subsurface temperature variability in the TC region (propagation from the northeastern Pacific) is inconclusive because the subsurface salinity propagation pathway is shown, not temperature. These major issues need to be addressed.

1. Evidence of the importance of subsurface temperature for super TCs.
   It's well known that SST is the most important factor affecting TC intensity. Subsurface conditions (ocean heat content, T/S stratification) affect TCs through their impact on SST. How can subsurface temperature (150 m depth) be more important than SST? I'm not aware of any other studies that come to a similar conclusion except maybe for specific case studies. In the present study, annual maximum LMI (defined as max TC intensity achieved by any TC in a given year?) is used instead of intensity, and LMI seems like a less robust metric compared to integrated/averaged metrics like ACE. More in-depth analysis is needed to explain why subsurface temperature is so much more important than SST.
   Is SST very uniform spatially and/or does is not change much year to year? If subsurface temperature has stronger variability than SST, that could help explain it. However, SST is 100% effective at modifying the surface enthalpy flux and TC intensity (any change in SST directly affects the energy exchange), whereas subsurface temperature, especially as deep as 100-200 m, is much less effective because it first has to be mixed/upwelled to the surface. If a warm temperature anomaly at 100 m depth is 1C and it decreases linearly to the surface, and the TC mixes completely down to 100 m, the subsurface temperature anomaly will result in an SST anomaly of only 0.5C. That means variability of subsurface temperature has to be twice as large as variability of SST just for subsurface temperature to have a similar impact as SST. However, you show in Fig. 2c that the influence of subsurface temperature dominates that of SST, and the maximum correlation is at ~150 m depth. That means that subsurface temperature variability has to be a lot larger than that of SST, probably at least 5-6 times larger. Is that the case?

Response:

We are grateful to you for highlighting the need to better elucidate the subsurface mechanism, and enlightening and constructive comments regarding the relative contributions of surface and subsurface layers in regulating SST during the TC-induced mixing and upwelling process. Following your valuable advice, we carried out a quantitative assessment of the relative contributions from surface and subsurface layers, and established a theoretical derivation explaining the crucial role of the subsurface layer in determining the SST evolution. According to our analysis, the upper ocean in the study region is composed of two distinctively different layers. The surface layer heat content shows a warming trend, while the subsurface layer exhibits a multi-decadal V-shaped variation with no long-term trend. Although the two have comparable amplitudes,

the variability in subsurface heat content is observed to be more than 20 times greater than that in the surface layer. This indicates that the subsurface layer plays a more significant role in driving changes in SST during mixing induced by the most intense TCs. For further details, please refer to our General Response 2 (page 2-6) and the related discussion can be found in Lines 398-424 of the revised manuscript.

Regarding the choice of the annual maximum LMI, we selected this metric because composite indices such as ACE and PDI are inherently influenced by storm frequency, track, and duration. We aimed to isolate these factors in order to focus more specifically on the ocean's influence on the most intense TCs. As demonstrated in our manuscript, the annual maximum LMI shows high consistency across four different agency datasets, supporting its robustness as an indicator. We acknowledge that this metric does not represent overall TC activity, and we have revised the manuscript in Lines 80-91 to clarify this point. Nevertheless, it remains an important measure of the ocean's role in controlling the upper limit of TC intensity.

2. Another possibility is that subsurface temperature is correlated with something else that affects LMI, such as TC atmospheric steering flow. Changes in the large-scale flow could keep TCs over more or less conducive conditions for intensification for longer or shorter amounts of time, resulting in changes in LMI that are physically unconnected to changes in subsurface temperature.
   At the same time, the steering flow anomalies could also force subsurface temperature changes through Ekman convergence/divergence, resulting in the observed correlation without causation.

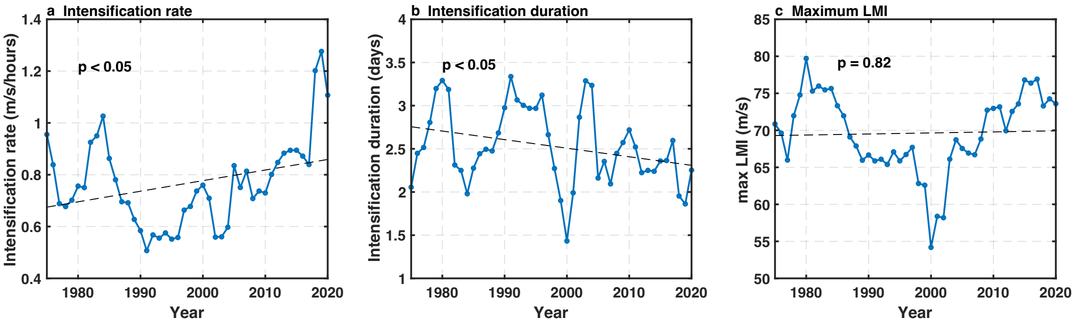

Fig. R10 *Time series of* ***(a)*** *mean intensification rate,* ***(b)*** *mean intensification duration for TCs with the annual maximum LMI and* ***(c)*** *the annual maximum LMI in the TC region. The black dashed lines indicate the linear regression trend, with the corresponding p-value indicated in each panel. The mean intensification rate/duration is calculated from the time when a TC's wind speed first reaches 30 m/s until its LMI. All time series are averaged by four agencies (USA, HKO, CMA and JMA).*

Response:

We thank you for your valuable suggestion. Given the potential influence of atmospheric variables on TC intensity, we have already incorporated analyses of several key atmospheric factors (including vertical wind shear, relative vorticity, and pressure vertical velocity) in our study. However, these variables do not show a statistically significant correlation with the annual maximum LMI on seasonal average.

To further address the mechanism that steering flow could prolong intensification duration

and thus affect LMI, we have conducted additional analysis that decomposes LMI into the mean intensification rate and the intensification duration (Mei et al., 2015). As shown in Fig. R10, the annual maximum LMI exhibits a much stronger correlation with the mean intensification rate (R=0.55) than with intensification duration (R=0.1). The weak observed link with duration argues against steering flow being the primary driver in the LMI interannual variability.

It should be noted that the mean intensification rate is calculated from 30 m/s up to LMI. During the initial phase of this period, when wind speeds are relatively low, intensification is likely more influenced by the near surface temperature, as mixing depth is relatively shallow and subsurface water entrainment is limited. This is consistent with the weak increasing trend observed in the mean intensification rate, which aligns with the observed surface warming. Therefore, the mean intensification rate inherently reflects a combination of surface and subsurface thermal influences, with the latter becoming more dominant during intensification at higher wind speeds.

3. Evidence of subsurface temperature propagation to the TC region. The analysis showing interannual variations in subsurface salinity in the TC region that are connected to formation regions in the northeastern Pacific makes sense. However, the argument made throughout the manuscript is that changes in subsurface temperature strongly affect TC LMI, not subsurface salinity. Does subsurface temperature show similar lat-lon distributions and lead-lag relationships as salinity? This is crucial to the argument of remote forcing and predictability of super TC activity in the western North Pacific.

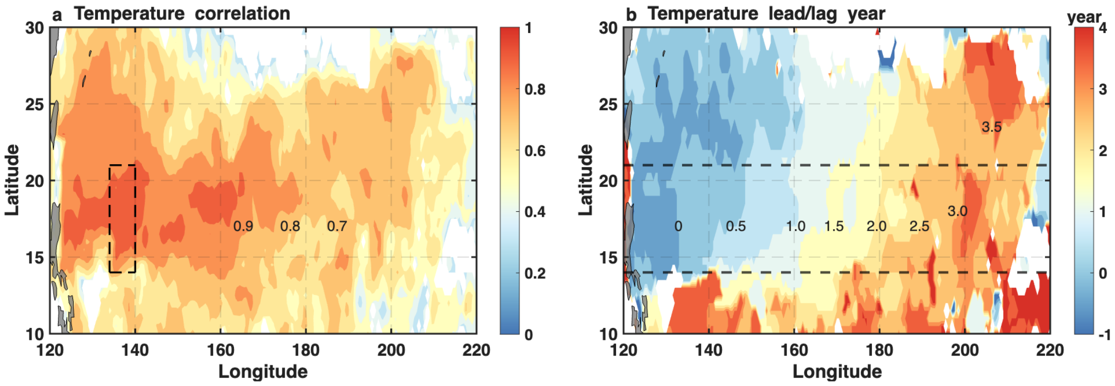

Fig. R11 The transport path of the subsurface temperature based on Argo data. (a) *Strongest correlations (above 95% confidence level) between the temperature within the box (134°-140°E, 14°-21°N, denoted by a black dashed box) and that of all other regions along the isopycnal 24 kg/m^3.* ***(b)*** *Lead/lag periods corresponding to the strongest correlations shown in* ***(b)****. Black dashed lines denote latitudes 14°N and 21°N respectively.*

Response:

We thank you for this important comment. In our original analysis, we chose salinity as a key tracer because it forms a distinct high-salinity core through the water column in the TC region, making the particular water mass easily identifiable. Subsurface maximum in salinity also makes it clear that the water mass cannot be formed locally from the surface, inspiring us to find its formation region by tracing its properties along the isopycnal. Due to the compensation relation between variations of salinity and temperature along a given isopycnal, the two are perfect aligned with

each other. We have performed the same analysis for temperature as we did for salinity in Fig.3 of the manuscript, and temperature displays consistent spatiotemporal patterns and lead-lag relationships to those of salinity (Fig. R11). This coherence is expected, as both properties are quasi-conserved during the transport process along isopycnals of the same water mass from its formation region. These findings have been added to the revised manuscript in Lines 255-260 and further support the role of this remote subsurface water mass in influencing annual maximum LMI in the western North Pacific.

Other comments:

4. Line 89: Please define 'super TC' (LMI > 70 m/s).

Response: we thank you for pointing out the imprecision of the text. We have defined 'super TC' upon its first occurrence in Line 121 of the revised manuscript.

5. Lines 101-104: Please explain why LMI has the most direct relationship with regional oceanic thermal conditions. For a given TC, it will depend strongly on atmospheric conditions such as wind shear and humidity. The maximum LMI over a whole year might also be very sensitive to atmospheric conditions. For example, if the ocean is the same in two different years, but a TC happens to experience much more conducive atmospheric conditions along its track in one year compared to the other (because of steering flow and/or synoptic weather variations), that year will have a higher total LMI despite no differences in the ocean. These subtleties deserve more consideration and discussion.

Response:

We appreciate your consideration regarding the use of LMI. We would like to clarify the variable used in this study is the annual maximum LMI. In any given year, many TCs may occur, and each reaching its own lifetime maximum intensity. The annual maximum LMI is defined as the highest LMI value among all TCs occurring in that year, yielding a single representative value per year for our analysis.

We agree that both atmospheric conditions and oceanic thermal structure influence TC intensity. Our reasoning for stating that annual maximum LMI has the most direct relationship with regional oceanic thermal conditions among commonly used TC metrics is based on the following considerations. First, from a theoretical perspective, Potential Intensity (PI) theory (Emanuel, 1995, 1997; Lin et al., 2013) establishes an upper-limit relationship between SST and the maximum attainable TC intensity, under the assumption that the storm remains in near-equilibrium with its oceanic and atmospheric environment. The LMI of a TC approaches this potential intensity when atmospheric conditions are relatively favorable. In this context, LMI is more directly tied to the ocean's thermal energy supply than other metrics such as PDI or ACE, which depend more on storm frequency, duration, and track distribution.

While PI is conventionally related to SST, the relevant SST is the under-storm SST, which is often cooler than the pre-storm SST due to upwelling and entrainment. Stronger TCs induce deeper mixing and entrain more subsurface water, making annual maximum LMI especially reflective of subsurface thermal influences, as it represents the year's most intense storm. In contrast, metrics such as mean LMI or integrated indices include weaker storms that may be more influenced by surface conditions or inhibited by unfavorable atmospheric factors.

We fully acknowledge that the LMI of an individual TC can be sensitive to atmospheric

variability. However, the annual maximum LMI can be interpreted as the upper intensity limit permitted by the ocean, particularly the subsurface ocean, under the year's most conducive atmospheric setting. By focusing on the strongest storm each year, we target events in which atmospheric conditions are likely more favorable and the subsurface ocean's role in intensity modulation is most pronounced. Furthermore, as shown in Fig. 2 of the manuscript, the annual maximum LMI exhibits no strong correlation with several large-scale atmospheric variables in the TC region, supporting the premise that it is primarily governed by oceanic processes. We have revised the manuscript in Lines 80-91 to more clearly explain this rationale. Thank you for prompting this clarification.

6. Fig. 1: Another interpretation is that TCs form to the south and generally move to the west/northwest and take time to reach max. intensity. That's why strong TCs are most common in the 15-20N region.

Response:

We thank you for raising this alternative interpretation. We have revised the manuscript in Lines 124-127 to acknowledge in our discussion regarding Fig. 1 that the higher occurrence of intense TCs in the 14°-21°N band may partly reflect the time required for TCs to reach their maximum intensity after forming farther south and tracking westward or northwestward.

Besides, we propose that the clustering of the strongest TCs in this region may also be related to the distinct subsurface thermal structure present there. While the dynamical process noted by you is well-recognized in TC development, our results further suggest that local subsurface conditions help modulate the intensity ceiling that TCs can attain. As demonstrated by our study, this unique oceanic environment exerts a significant influence on interannual-to-multidecadal variation of the annual maximum LMI.

7. There are different metrics used in the manuscript and they are not always well defined: super TC strength ("maximum LMI of all TCs attained in each year" - maximum LMI across all TCs (a single value) or LMI averaged across all TCs?), "annual maximum LMI" (Fig. 2 caption), "maximum LMI" (line 204). More generally, the use of "maximum" to describe LMI is confusing because LMI is "lifetime maximum intensity." What does "maximum lifetime maximum intensity" mean?

Response: We thank you for pointing out the lack of clarity in our terminology. We sincerely apologize for the confusion caused by the inconsistent use of names in the manuscript. The metric used throughout our study is the annual maximum LMI. For each year, multiple TCs may occur, and each will reach its own lifetime maximum intensity. The annual maximum LMI is defined as the single highest LMI value among all TCs in that given year. It is a single, yearly value, not an average across TCs. To prevent any misunderstanding, we have revised the manuscript to consistently use the precise term "annual maximum LMI" and have clearly defined it upon its first occurrence in Lines 80-81 of the revised manuscript.

8. Lines 334-336: I don't understand the statement "underlying SST has already lost its pre-TC signature." The pre-storm SST still matters, whether it's 27C or 29C. The TC-induced

cooling starts from the pre-storm value. If pre-storm SST is lower, under-storm SST will be lower. In that respect, the pre-storm SST signature remains. Even if the impact of SST on TC intensification is nonlinear, for example if SST=24 and SST=26 have the same effect, the pre-storm SST helps determine whether that nonlinear threshold will be reached. On the other hand, if super TCs are so strong that they always bring SST below the nonlinear threshold, then variations of subsurface temperature will not impact TC intensity.

Response: We thank you for this insightful comment. We agree that the pre-storm SST provides the initial condition for TC-induced cooling and therefore influences the final SST under the storm. Our statement that the "underlying SST has already lost its pre-TC signature" was inaccurate and we have revised this description in the manuscript in Lines 352-354. What we intended to convey is that the under-storm SST is no longer the same as pre-SST. Through vertical mixing, it becomes a blended temperature reflecting both surface and subsurface thermal properties. Our quantitative analysis confirms that while the pre-storm surface layer heat content remains a contributing factor to the under-storm SST, the temporal variability of the under-storm SST is predominantly controlled by the variability of the subsurface layer (please refer to our General Response 2 (page 2-6). We have modified the phrase to clarify this point and to more accurately describe the respective roles of the surface and subsurface layers in controlling the under-storm SST evolution.

9. Line 639: A translation speed of 3 m/s if very low. The observed mean translation speed is probably closer to 6 m/s. Therefore, please verify that the modeled upwelling agrees well with the upwelling estimated from the Argo profiles. Please also verify that the model produces TC-induced SST cooling that is similar to observed values.

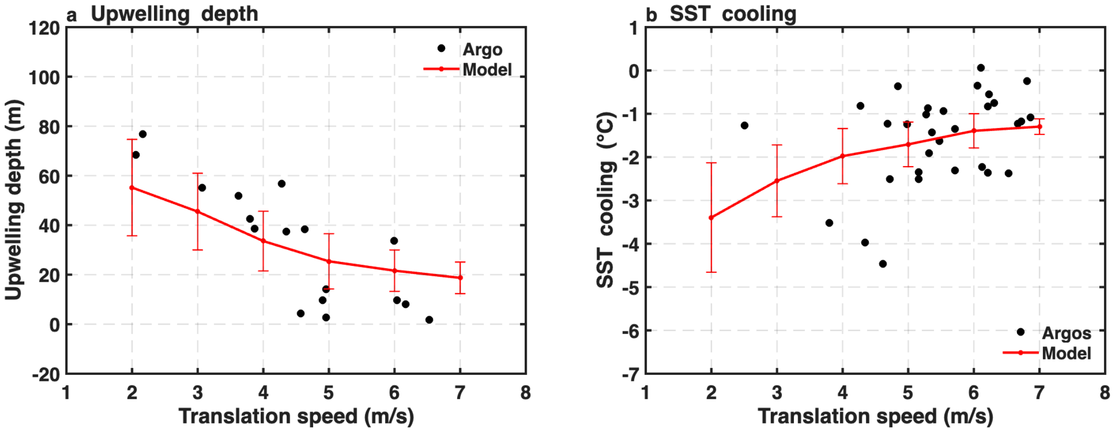

Fig.R12 Verification of model-induced upwelling and sea surface temperature (SST) cooling. (a) *Observed upwelling depth based on Argo (black dots) and model-simulated upwelling depth (red).* ***(b)*** *Observed SST cooling based on Argos (black dots) and model-simulated SST cooling (red). Argo/Argos are sampled within 0-7 days after TC passage and within 50 km across the TC track, considering only strong TCs (Category 4 and 5). The model simulation is forced with a wind speed of 80 m/s. Red lines show the mean value within 50 km across the track, averaged within 0-7 days after TC passage, with error bars indicating one standard deviation.*

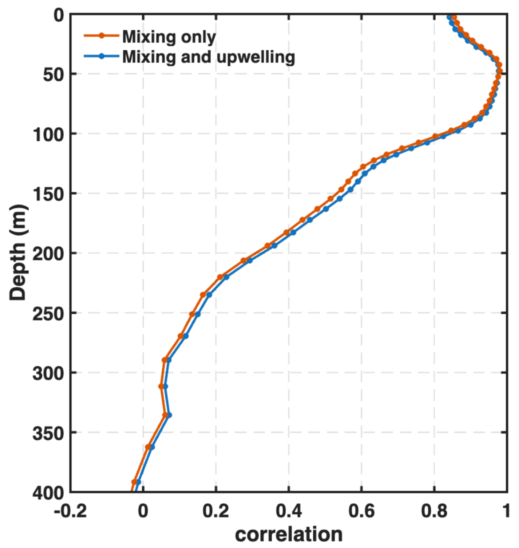

Fig.R13 The correlation between the modelled under-storm SST and the pre-storm temperature at translation speed of 6 m/s. *The red dot line denotes the mixing-only case and the blue dot line denotes the mixing+upwelling case. Model configurations are the same as in Fig. 5 of the manuscript but for a higher translation speed.*

Response:

We thank you for raising this important point regarding translation speed. Firstly, we verified the model simulations against observations (Fig. R12). Argo profiles are used to validate the simulated upwelling depth, and Argos (floats drifting near the ocean surface) are used to validate the simulated SST cooling. The results show good agreement, indicating that the model captures the ocean response reasonably well.

We agree that the typical observed TC translation speed is higher than model set up in Fig. 4 of the manuscript. At a translation speed 6 m/s (Fig. R13), upwelling becomes less effective. The under-storm SST is mainly modulated by the mixing process. The slower speed (3 m/s) adopted in our experiment was not intended to replicate the most common scenario of the ocean, but rather to create a demonstrative, idealized setup to maximize the difference between upwelling and no-upwelling cases.

We originally hypothesized that the TC-induced upwelling, which effectively brings more subsurface water into mixing whereas pushes surface water away from the TC track, is most crucial for the decisive role of subsurface water temperature on the SST evolution during a TC's passage. Nevertheless, following your enlightening comments regarding the physical mechanism, we discovered that it is the much greater variability of the thermal condition of the subsurface layer that caused its dominance in modulating SST evolution. Although the subsurface effects can be amplified by upwelling, the high degree of temporal variation of this particular water mass is essential.

10. Lines 655-656: "disabling horizontal advection" - In the main text it's stated that horizontal divergence is set to zero. Which one is it?

Response:

We thank you for identifying this inconsistency. The statement in the text, " turning on/off divergence of the horizontal flow" was indeed incorrect and should have been "disabling horizontal advection."

We have now corrected the corresponding description in Lines 348-349 of the revised manuscript to ensure consistency throughout the paper. We sincerely apologize for this oversight and greatly appreciate the reviewer's careful attention.

Reference:

Mei, W., Xie, S. P., Primeau, F., McWilliams, J. C., & Pasquero, C. Northwestern Pacific typhoon intensity controlled by changes in ocean temperatures. Sci. Adv. ***1****, e1500014 (2015).*

Emanuel, K. A. Sensitivity of tropical cyclones to surface exchange coefficients and a revised steady-state model incorporating eye dynamics. J. Atmos. Sci. ***52****, 3969–3976 (1995).*

Emanuel, K. A. Some aspects of hurricane inner-core dynamics and energetics. J. Atmos. Sci. ***54****, 1014–1026 (1997).*

Lin, I. I. et al. An ocean coupling potential intensity index for tropical cyclones. Geophys. Res. Lett. ***40****, 1878–1882 (2013).*

Reviewer #1 (Remarks to the Author):

The authors have addressed my comments from the last round of reviews fairly well. I only have two additional suggestions for them.

We appreciate the reviewer's positive feedback on our previous revision. The reviewer's insightful suggestions from the last round have been instrumental in improving the quality of our manuscript. We are also grateful for the additional suggestions provided this time, which we have carefully addressed as follows.

1) It is nice that they have shown the relationship between the North Pacific high in the subtropical eastern Pacific and TC activity in the western Pacific. While they have used observations to demonstrate this, I wonder if they can confirm the robustness of this relationship (eg. ~5 years) using models. One possibility is the use of TC tracks from the HighResMIP suite of models, which are already provided here:

https://catalogue.ceda.ac.uk/uuid/e82a62d926d7448696a2b60c1925f811/.

Response:

We thank the reviewer for the valuable suggestion. Following this recommendation, we analyzed six high-resolution models from HighResMIP (Roberts et al., 2020). In summary, these models fail to reproduce the observed linkage between early sea surface signals in the eastern North Pacific and the subsequent TC maximum intensity in the western North Pacific. This limitation can be attributed to two main factors. First, the models systematically underestimate TC wind speeds. The simulated TC intensities are only about half of the observed values, which may lead to insufficient mixing of subsurface ocean waters. Second, the modelled water masses originating in the eastern North Pacific subduction region are not well conserved during their westward transport, thereby failing to retain the climatic signals at the source region.

The six high-resolution models from HighResMIP are: CMCC-CM2-VHR4, CNRM-CM6-1-HR, EC-Earth3P-HR, ECMWF-IFS-HR, HadGEM3-GC31-HM, and MPI-ESM1-2-XR. Basic information for each model is provided in Tab. R1. The TC track and wind speed data from the HighResMIP models used in our analysis were derived using the "TRACK" algorithm (Roberts, 2019). We utilize the simulated data derived from historical ocean-atmosphere coupled experiments (hist-1950) over the period 1975-2014, which aligns with the temporal coverage of the observations.

Tab. R1 Concise list of HighResMIP model configurations.

Model name	*Atmospheric resolution (km)*	*Ocean resolution (°)*	*Oceanic model levels*	*Reference*
CMCC-CM2-VHR4	*25*	*0.25*	*50*	*Scoccimarro et al., 2017*
CNRM-CM6-1-HR	*50*	*0.25*	*75*	*Voldoire, 2019*
EC-Earth3P-HR	*50*	*0.25*	*75*	*EC-Earth, 2018*
ECMWF-IFS-HR	*25*	*0.25*	*75*	*Roberts et al., 2017*

HadGEM3-GC31-HM	*50*	*0.08*	*75*	*Roberts, 2017*
MPI-ESM1-2-XR	*50*	*0.4*	*40*	*von Storch et al., 2017*

Firstly, we compare the latitude and intensity distributions of TC LMI over the western North Pacific between observations and HighResMIP model simulations (Fig. R1). To examine the latitude distribution of the most intense TCs, we used the top 20% of LMI values as an indicator of strong TCs. As shown in Fig. R1a, the simulated latitude distribution of the most intense LMI exhibits a bimodal structure, with a primary peak between 10°-30°N and a secondary peak between 30°-50°N. The secondary peak is related to the TC tracking algorithm, which tends to misidentify extratropical cyclones in the westerly belt as TCs, resulting in a spurious high-latitude peak (Horn et al., 2014; Li and Zhou, 2024). Comparison between the multi-model ensemble mean and observations reveals that the observed LMI distribution peaks at approximately 18°N, while the ensemble mean peaks near 22°N, indicating a northward shift in the models.

Given the spurious high-latitude signals, we confine the analysis to regions south of 30°N to more accurately assess the models' capability in simulating TC intensity. As shown in Fig. R1b, models consistently underestimate TC intensity. The multi-model means of the top 20% LMI reaches only 35 m s^{-1}, about half the observed value of 68 m s^{-1}, a bias consistent with findings of previous work (Camargo, 2013; Guan et al., 2026).

Fig.R2 shows the vertical mean temperature of the upper 200 m for each model. Overall, all models capture a pronounced warm region in the subtropical zone. Based on this warm pool location and the northward-biased TC distribution in the models, we define the analysis region for TCs as 120°-150°E and 14°-25°N.

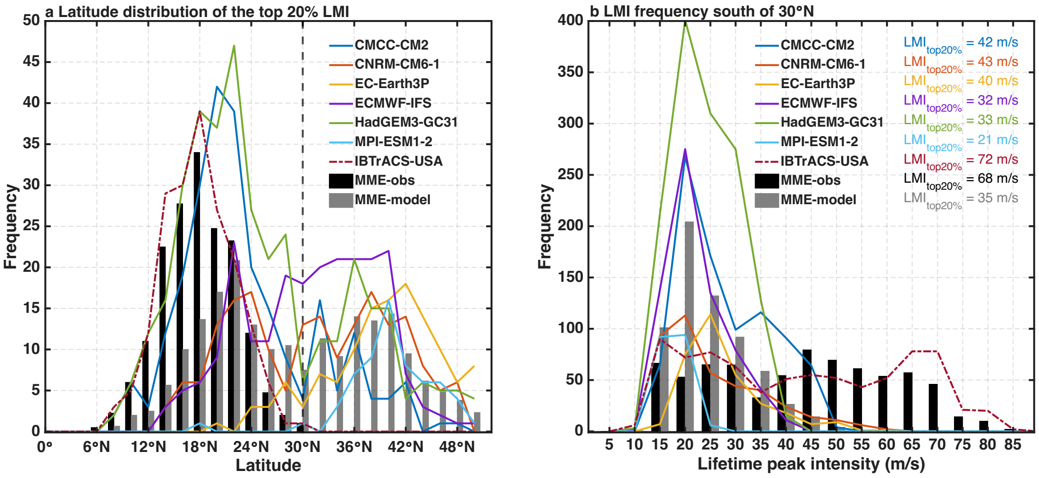

Fig.R1 Comparison of the latitude and intensity distributions of TC LMI between observations and HighResMIP models in the WNP during 1975-2014. *(a) Latitude distribution of the top 20% LMI in the WNP (120°-180°E, 0°-50°N). Colored solid lines represent the six individual HighResMIP models, and the gray bars indicate their multi-model ensemble mean (MME). Black bars represent the MME of four observational datasets (USA, CMA, HKO, JMA) from IBTrACS. (b) Intensity distribution of LMI. Same as (a), but for the distribution of LMI for all storms occurring*

south of 30°N. The top 20% mean LMI for each model is denoted in the upper-right corner of the panel.

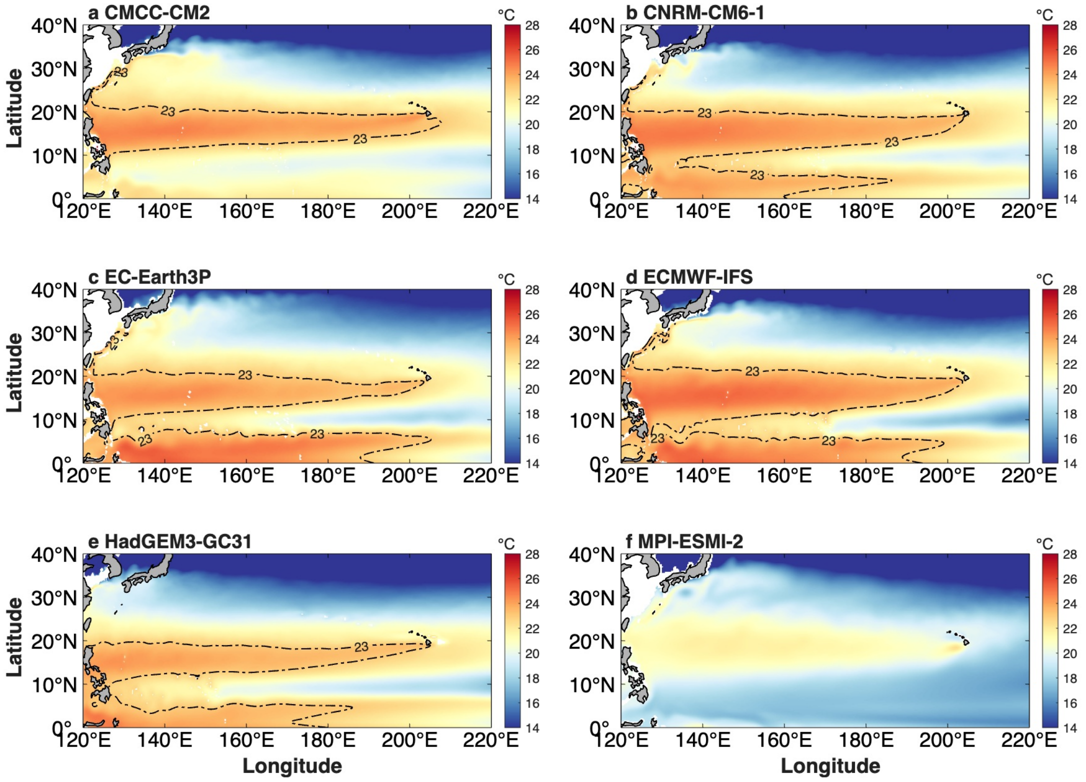

Fig.R2 Mean temperature of the upper 200 m in HighResMIP models. *(a)-(f) are for the CMCC-CM2, CNRM-CM6-1, EC-Earth3P, ECMWF-IFS, HadGEM3-GC31 and MPI-ESM1-2 models, respectively. The black dashed lines indicate the 23℃ isotherm.*

We calculated the annual maximum LMI time series for each model over the defined region during 1975-2014. The results reveal substantial inter-model differences in interannual variability, with no consistent trend across models (Fig. R3a). Further analysis of the relationship between annual maximum LMI and ocean temperature shows that only HadGEM3-GC31 exhibits a strong correlation with mixed layer temperature. This may be due to the model's underestimated wind speeds, which lead to a shallower mixing depth and insufficient mixing of subsurface waters, thereby making LMI more sensitive to ocean surface conditions. In contrast, the remaining five models show weak correlations between annual maximum LMI and ocean temperature at various depths, suggesting that ocean temperature is not a dominant factor modulating interannual variations in LMI within these models (Fig. R3b).

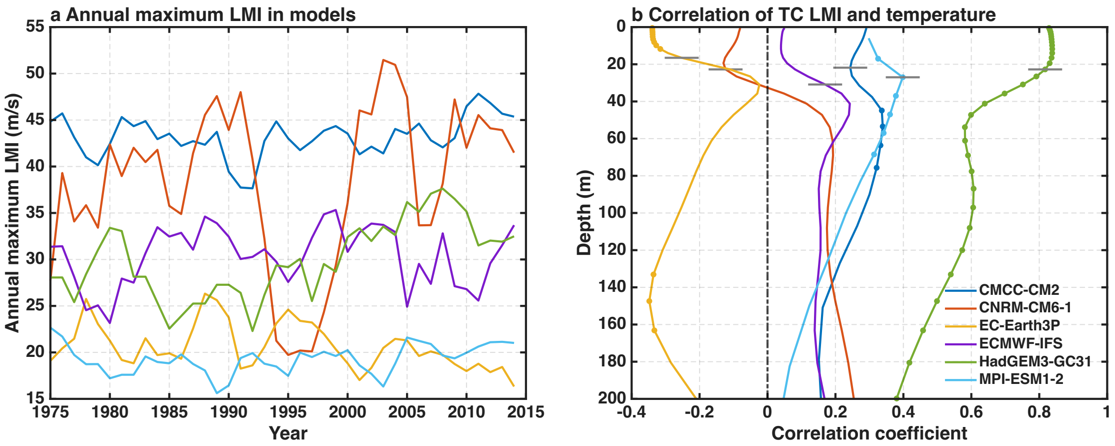

Fig.R3 Relationship between annual maximum LMI of TCs and ocean temperature in HighResMIP models during 1975-2014. *(a) Time series of the annual maximum LMI within the region 120° -150° E, 14° -25° N for six models. (b) Correlation coefficients between the annual maximum LMI and the temperature through the water column for six models. Temperature is averaged over the same region 120° -150° E, 14° -25° N. Color dots show results statistically significant ($p<0.05$). Gray bars denote the mixed layer depth in each model. A 3-year running mean smoothing was applied to all time series.*

We also examine the transport pathways of the subsurface water mass in the models (Fig.R4). Along the isopycnal 24.5 kg m^{-3}, we calculated lead/lag correlations of temperature between the key area (134°-140°E,14°-25°N, denoted by a rectangular box in Fig. R4) and other regions across the North Pacific.

The results indicate that, during westward transport, the conservation of subsurface water masses is generally weaker in the models than in observations. With the exception of CMCC-CM2 and HadGEM3-GC31, regions with correlations exceeding 0.7 (black dashed contours in Fig. R4) rarely extend eastward to 200°E. This implies that the properties of water masses formed beneath the NPH can hardly be conserved during their transport to the western boundary.

This limitation may be linked to differences in the simulated ventilation positions among the models. Fig. R5 presents latitudinal sections of climatological salinity, which help to identify where water masses are ventilated. In particular, CNRM-CM6-1 shows the poorest conservation of water mass characteristics, with significant correlation lost as early as 160°E. This aligns well with its distinct westward-shifted ventilation position along the 23 kg m^{-3} isopycnal, as evident in Fig. R5b. Such differences in ventilation location may render water masses more susceptible to surface processes during their subsequent transport, thereby hindering the preservation of source region characteristics.

Moreover, the formed water mass may dissipate locally (Guo et al., 2018) and thus cannot persist until the following year, when it would otherwise subduct into the thermocline and continue its westward transport. Consequently, the NPH signal is not well preserved, which may partly explain the relatively weak conservation of water mass transport in the models.

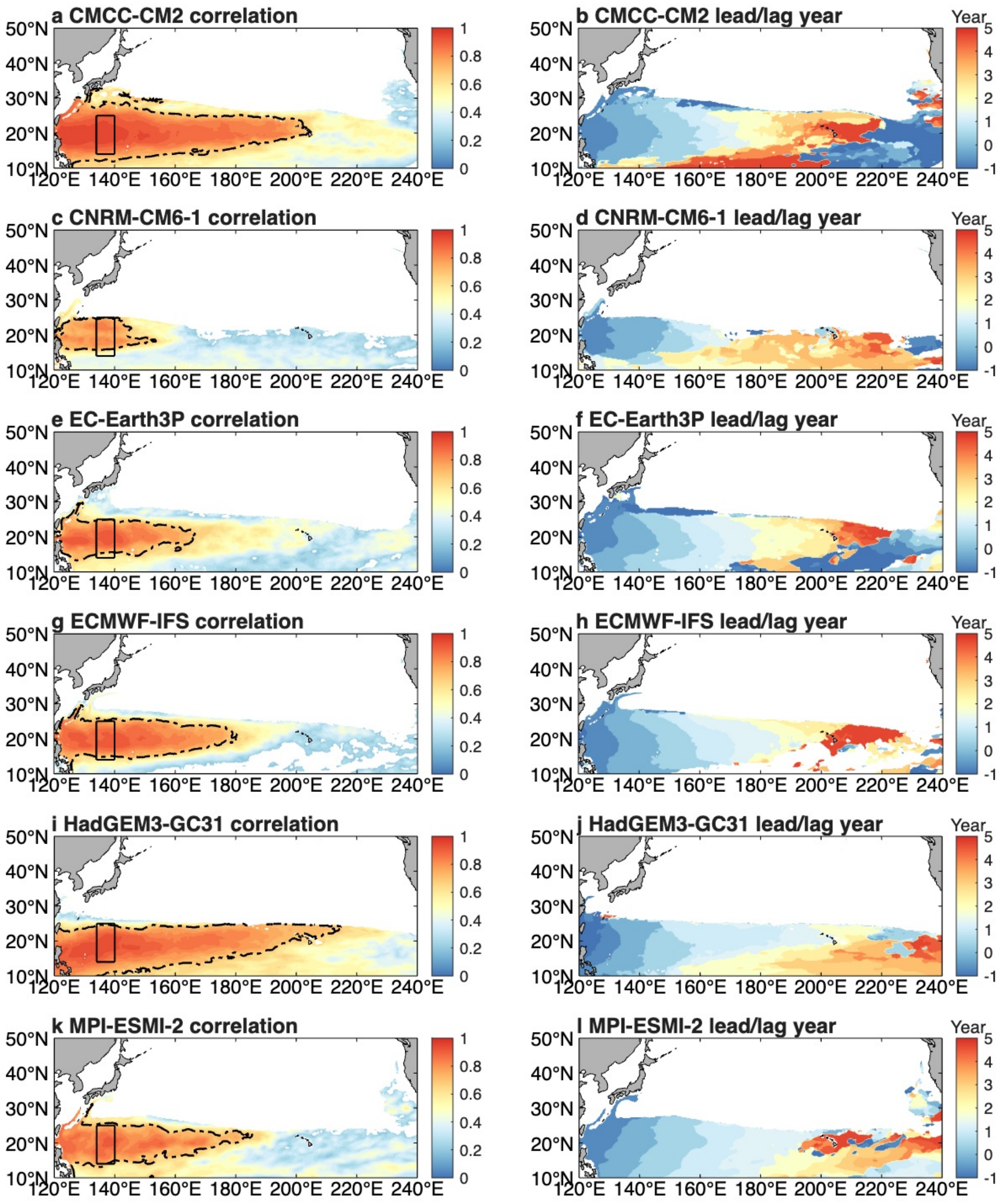

Fig.R4 The transport path of the subsurface water in HighResMIP models. *(a, c, e, g, i, k) Strongest correlations (above 95% confidence level) between the temperature within the black box (134° -140° E, 14° -25° N) and that of all other regions along the isopycnal 24.5 kg m*$^{-3}$*. The black dashed contour denotes a correlation of 0.7. (b, d, f, h, j, l) The corresponding lead/lag time at which the strongest correlation occurs. The black dashed contour denotes a correlation of 0.7. Results are shown for: (a, b) CMCC-CM2; (c, d) CNRM-CM6-1; (e, f) EC-Earth3P; (g, h) ECMWF-IFS; (i, j) HadGEM3-GC31; (k, l) MPI-ESM1-2.*

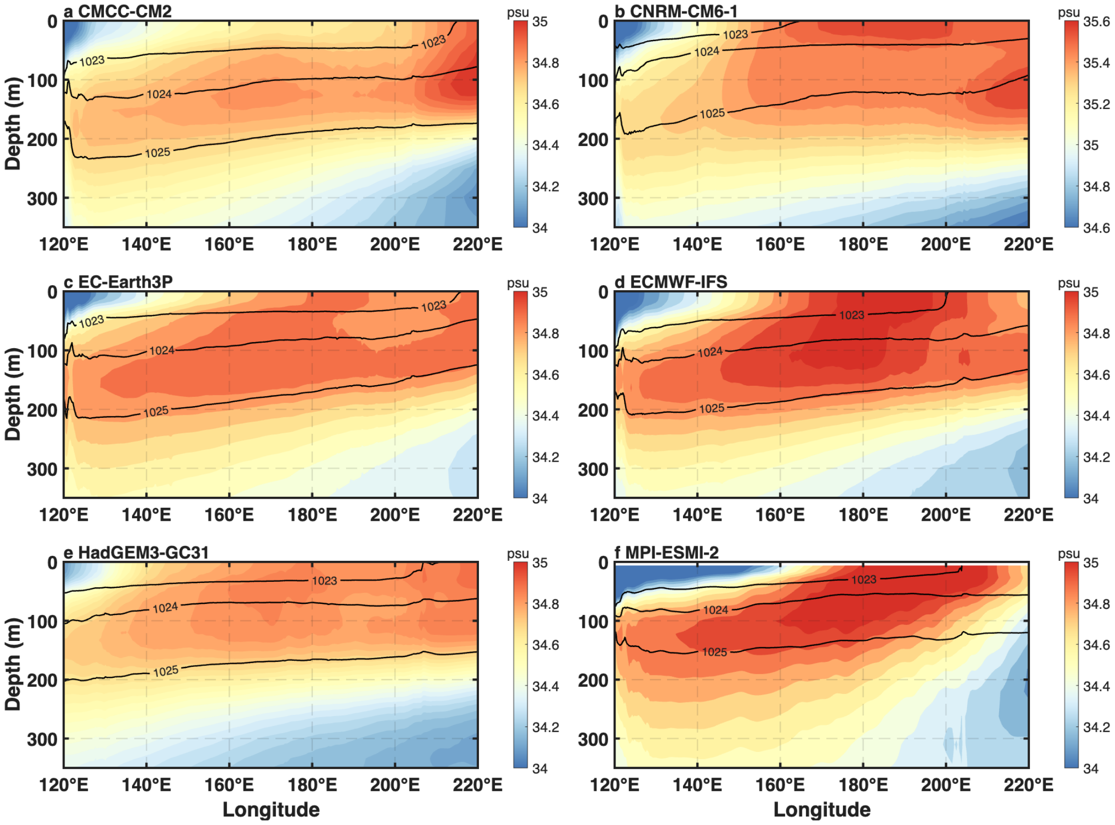

Fig.R5 Latitudinal sections of climatological salinity in the WNP simulated by HighResMIP models. *(a)-(f) are for the CMCC-CM2, CNRM-CM6-1, EC-Earth3P, ECMWF-IFS, HadGEM3-GC31 and MPI-ESM1-2 models, respectively. The sections are obtained by averaging the salinity field over 14° -25° N. Black contours denote the 23, 24, and 25 kg m*$^{-3}$ *isopycnals.*

In summary, local ocean temperature is not the dominant factor modulating the annual maximum LMI of TCs over the western North Pacific in the HighResMIP models. Meanwhile, although the models capture, to some extent, the cross-basin transport pathway of subsurface water masses, these water mass properties are not well conserved during their westward transport. Further improvements in simulating TC intensity and its coupling with oceanic processes are still needed. We have incorporated this analysis into the discussion section of the revised manuscript (Lines 519-537).

2) To complement the existing analysis in Fig. 4, I suggest showing composites of surface pressure (and winds/MLD) over the NPSH region for years with strong and weak TCs in the western Pacific. Beyond a simple correlation, this would help see things a bit more clearly.

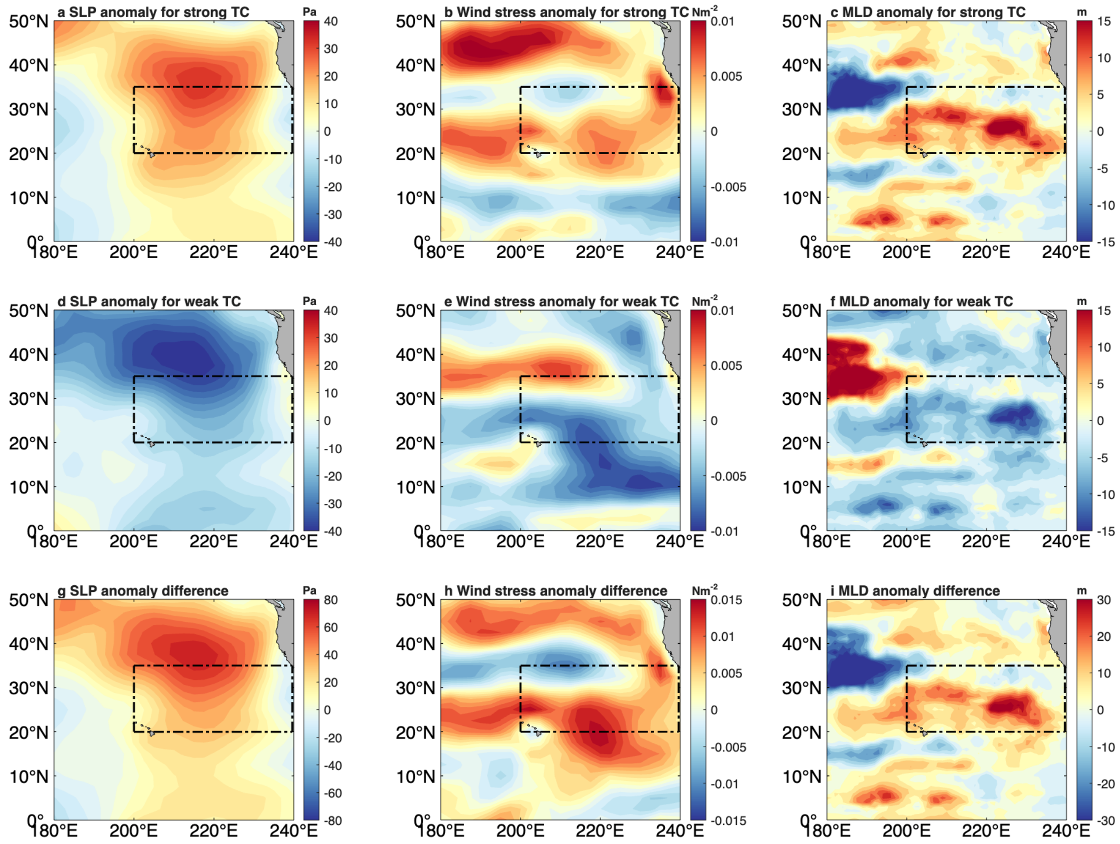

Fig.R6 Composite analysis of ocean and atmospheric conditions five years prior to strong and weak TC events. *Shown are sea level pressure (SLP) anomaly (a, d, g), March wind stress anomaly (b, e, h), and January-March mean mixed layer depth (MLD) anomaly (c, f, i). The composite fields are shown for (a-c) strong TC events, (d-f) weak TC events, and (g-i) their anomaly differences (strong minus weak). The black dashed box (200°-240°E, 20°-35°N) denotes the core water mass formation area.*

Response:

We thank the reviewer for the valuable suggestion. Following this recommendation, we constructed composites of SLP, wind stress, and MLD over the NPSH region for years with strong and weak TC activity.

To objectively distinguish between strong and weak TC years, we used the standardized time series of annual maximum LMI (orange line in Fig. 4c of the manuscript). Years with standardized values greater than 0 were defined as strong TC events (1976, 1978-1986, 2009-2020), and those with values less than 0 as weak TC events (1975, 1977, 1987-2008). Composite analyses were then performed for the five years preceding each type of event to examine precursory signals in the ocean-atmosphere system. The black dashed lines in Fig.R7 denote the water mass formation area (200°-240°E, 20°-35°N).

The results indicate that NPH intensity anomalies significantly modulate regional wind stress and MLD patterns, with distinctly opposing patterns prior to strong versus weak TC events. Prior

to strong TC events, the NPH is intensified, accompanied by an enhanced high-pressure system over the eastern North Pacific (Fig. R6a) and increased wind stress around its periphery (Fig. R6b). These conditions may enhance oceanic heat loss, deepen the mixed layer in water mass formation regions (Fig. R6c), and thereby influence the formation and physical characteristics of water masses. In contrast, prior to weak TC events, the NPH is relatively weak (Fig. R6d), with reduced wind stress (Fig. R6e) and diminished ocean heat loss, resulting in a shallower mixed layer (Fig. R6f). The differences between the composite fields further emphasize these contrasting patterns, illustrating systematic and opposing air-sea conditions in the eastern North Pacific preceding strong versus weak TC events (Figs. R6g-i). These results are discussed in Lines 312-325 of the revised manuscript.

References:

1. *Roberts, M. J., Camp, J., Seddon, J., et al. Impact of model resolution on tropical cyclone simulation using the HighResMIP-PRIMAVERA multimodel ensemble. Journal of Climate, 33(7), 2557-2583(2020).*
2. *Roberts, M. CMIP6 HighResMIP: tropical storm tracks. Centre for Environmental Data Analysis. http://catalogue.ceda.ac.uk/uuid/e82a62d926d7448696a2b60c1925f811 (2019).*
3. *Scoccimarro, E., Bellucci, A., & Peano, D. CMCC CMCC-CM2-VHR4 model output prepared for CMIP6 HighResMIP. Earth System Grid Federation. https://doi.org/10.22033/ESGF/CMIP6.1367 (2017).*
4. *Voldoire, A. CNRM-CERFACS CNRM-CM6-1-HR model output prepared for CMIP6 HighResMIP [Data set]. Earth System Grid Federation. https://doi.org/10.22033/ESGF/CMIP6.1387 (2019).*
5. *EC-Earth Consortium. EC-Earth-Consortium EC-Earth3P-HR model output prepared for CMIP6 HighResMIP. Earth System Grid Federation https://doi.org/10.22033/ESGF/CMIP6.2323 (2018).*
6. *Roberts, C. D., Senan, R., Molteni, F., et al. ECMWF ECMWF-IFS-HR model output prepared for CMIP6 HighResMIP. Earth System Grid Federation https://doi.org/10.22033/ESGF/CMIP6.2461 (2017).*
7. *Roberts, M. MOHC HadGEM3-GC31-HM model output prepared for CMIP6 HighResMIP. Earth System Grid Federation https://doi.org/10.22033/ESGF/CMIP6.446 (2017).*
8. *von Storch, J.-S., Putrasahan, D., Lohmann, K., et al. MPI-M MPI-ESM1.2-XR model output prepared for CMIP6 HighResMIP. Earth System Grid Federation https://doi.org/10.22033/ESGF/CMIP6.10290 (2017).*
9. *Horn, M., Walsh, K., Zhao, M., et al. Tracking scheme dependence of simulated tropical cyclone response to idealized climate simulations. Journal of climate, 27(24), 9197-9213 (2014).*
10. *Li, Z., & Zhou, W. Poleward migration of tropical cyclones over the western North Pacific in the CMIP6-HighResMIP models constrained by observations. npj Climate and Atmospheric Science, 7(1), 161 (2024).*
11. *Camargo, S. J. (2013). Global and regional aspects of tropical cyclone activity in the CMIP5 models. Journal of Climate, 26(24), 9880-9902.*
12. *Guan, S., Huang, M., Cai, W., el al. Weak self-induced cooling of tropical cyclones amid fast sea surface warming. Nature Geoscience, 19, 153-158 (2026).*
13. *Guo, Y., Lin, X., Wei, M., et al. Decadal variability of North Pacific eastern subtropical mode water. Journal of Geophysical Research: Oceans, 123(9), 6189-6206 (2018).*

Reviewer #3 (Remarks to the Author):
Second review of "Subsurface messenger for the annual maximum LMI of tropical cyclones in the western North Pacific" by Ni et al.

The authors have responded very well to the reviewers' comments and have provided more robust and in-depth analyses to support the main conclusions. These include (1) comparisons of the impact of subsurface temperature on maximum LMI to the impacts of other environmental conditions such as wind shear, humidity, and vorticity, showing that subsurface temperature is most important; (2) analysis of atmospheric forcing of subduction in the eastern Pacific and the connection to subsurface temperature in the western Pacific; (3) quantitative analysis supporting the greater impact of subsurface temperature variability on under-TC SST compared to pre-storm SST.

We thank the reviewer for the affirmation of our previous response. The reviewer's professional and precise summary of the key improvements in our revised work accurately reflects the main points we aimed to address. The thoughtful and insightful comments from the last round have greatly strengthened the quality of our manuscript.

I have a few more comments aimed at making the analysis and conclusions even more robust:

Lines 80-91: Why not use an upper quantile (90% or 95%) as in previous studies (Elsner et al. 2008)? Using the maximum intensity achieved by any single TC in a year can be strongly affected by any random confluence of synoptic weather conditions. For example, this was likely the case for Hurricane Patricia in the eastern Pacific, which was a very small hurricane and had nearly ideal SST and atmospheric conditions as it moved quickly, making subsurface ocean conditions unimportant. Cases like that probably don't have a very big impact on decadal variability in the western Pacific, which has high annual TC counts, but it would be helpful to know that your results are not sensitive to the TC intensity threshold for reasonable choices (top 10%, 5%, 1% of LMI, for example).

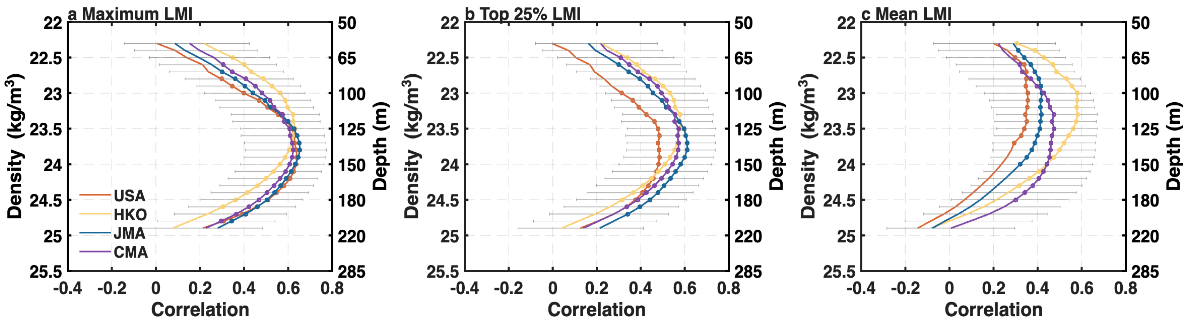

Fig.R7 Correlation between TC intensity and ocean temperature. *Results are for (a) Maximum LMI; (b) Mean LMI of the top 25% and (c) Mean LMI of all TCs. Colored markers indicate statistically significant results ($p < 0.05$). Gray bars represent the 95% confidence intervals based on the IBTrACS-CMA dataset. A 3-year running mean was applied to the time series.*

Response:

We thank the reviewer for the valuable suggestion. Following this recommendation, we

calculated the simultaneous correlations between ocean temperature and three TC metrics: the maximum LMI, the top 25% LMI, and the overall mean LMI.

Since the key region in the western North Pacific typically experiences about 10 TCs per year, using the top 10%, 5%, or 1% quantiles would essentially select only the most intense TC each year, which is equivalent to using the maximum LMI. To examine a slightly broader sample while still focusing on strong TCs, we instead chose the top 25% LMI (which corresponds to about 2-3 TCs per year). As shown in Fig. R7, the correlations obtained with the maximum LMI and the top 25% LMI are almost identical (Figs. R7ab), indicating that the use of the maximum LMI is robust and appropriate.

However, when the mean LMI of all TCs is used, the results differ markedly (Fig. R7c). First, the correlations decrease substantially, with maximum values ranging from 0.3 to 0.6 across different agencies. Second, the spread among the four agencies increases, reflecting greater inconsistency in the results. Third, the depth of the maximum correlation becomes shallower and is poorly defined, with a broad range of depths exhibiting similar correlation values. This suggests that including weaker TCs may introduce larger discrepancies among different agencies due to measurement uncertainties. It also implies that weaker wind speeds are associated with reduced ocean mixing, leading to a larger influence from the surface layer. Therefore, we use the maximum LMI, or the top 25% LMI as the TC metric in our analysis. These findings, which further support the robustness of our results, have been incorporated into the revised manuscript (Lines 476-482).

The variable that connects changes in subsurface temperature to LMI is TC-induced SST cooling (i.e., cold wake). With satellite SST, this can be calculated accurately since 1982. Are SST cold wakes weaker when max LMI is higher? It would be helpful to see a plot like Fig. 2d for max LMI and average cold wake to make a direct connection between subsurface temperature and max LMI.

Response:

We thank the reviewer for this insightful suggestion. Following this recommendation, we calculated the top 25% LMI and the corresponding TC-induced SST cooling (SSTC). As shown in Fig. R8, the results do not reveal a clear relationship between the two time series.

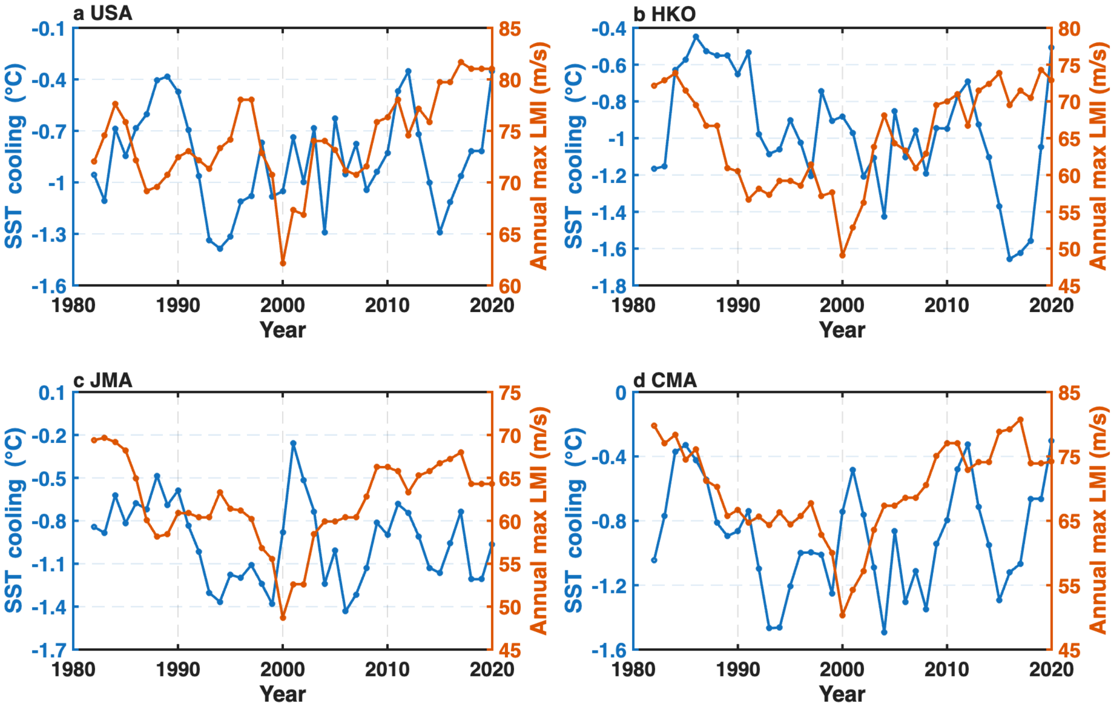

Fig.R8 Time series of the top 25% LMI and the corresponding TC-induced SST cooling. *Results are from four agencies: (a) USA, (b) HKO, (c) JMA and (d) CMA. The SST cooling is calculated as the difference between the average SST (within a 100 km distance of the maximum LMI location) three days before and after TC passage. A 3-year running mean smoothing was applied to all time series.*

The inconsistency between TC intensity and SSTC appears counterintuitive at first, but it is not unreasonable considering an important complicating factor that has been overlooked. As shown in Equation (1), the time series of SST cooling, $SSTC(t)$*, is calculated as the difference between the pre-storm* $SST(t)$ *and the mixed layer temperature after TC passage (*$T_{mix}(t)$*):*

$$SSTC(t) = SST(t) - T_{mix}(t) = SST(t) - \frac{\int_{-H_{mix}}^{0} T(z,t)dz}{H_{mix}} \qquad (1)$$

Specifically, T_{mix} *is calculated as the mean temperature of the pre-storm temperature profile* $T(z,t)$ *over the TC-induced mixing depth* H_{mix}*. In this formulation,* $SSTC(t)$*,* $SST(t)$*,* H_{mix}*, and the* $T(z,t)$ *are all time-dependent, with* $T(z,t)$ *also varying in the vertical indicating the warm water parcels on top of the cold ones.*

If we assume that H_{mix} *remains constant (e.g., fixed from year to year), then, based on our earlier discussion, a higher subsurface ocean heat content tends to mitigate SSTC, thereby favoring TC intensification, whereas a lower heat content enhances cooling and limits TC intensity. This reasoning would suggest that stronger TCs are associated with weaker SSTC.*

However, as a TC intensifies, its wind speeds increase, leading to a bigger H_{mix}*. Since the temperature profile* $T(z,t)$ *decreases monotonically with depth, a bigger* H_{mix} *means entrainment of more and colder water from below to reduce the* T_{mix} *and amplify SSTC. Consequently, even in the presence of high subsurface heat content, deeper mixing can still cause substantial cooling at the surface.*

Given this complex interplay of multiple factors and the inherently intricate relationship between SSTC and TC intensity, as well as the fact that the subsurface thermal structure influences both the potential for SSTC and the mixing depth, we focus in this study on the direct relationship between subsurface temperature and the maximum LMI.

What is the average translation speed for TCs with max LMI in the region you consider? This would help to put the mixing depths in context (Figs. S7, S9).

Response:

The average translation speed for TCs with maximum LMI in the key region is ~ 5m s^{-1}*, corresponding to a TC-affected depth of about 180 m.*

Lines 53-55: The interplay of these different factors for SST cooling is also described nicely in this recent paper: doi:10.1029/2024JC021610.

Response:

We thank the reviewer for this suggestion. The suggested paper indeed provides a clear explanation of the various factors influencing SST cooling. Since this discussion is highly relevant to the context in Lines 53-55, we have now cited this article in the revised manuscript (Line 54).

Review for “The subsurface messenger for the maximum intensity of tropical cyclones in the western North Pacific,” submitted to Nature Communications.

Dear Editor,

This manuscript seeks to understand oceanic controls behind the annual lifetime maximum intensity (aLMI) of TCs in the western north Pacific Ocean. To do so, the authors first show that the empirical frequency of TCs with wind speed > 70 m/s is greatest at the latitude (typhoon alley) where both the ocean surface and subsurface are above 26 degrees Celsius (Fig. 1b). As further confirmation, the authors compute correlations between many variables and aLMI in the region, and find that ocean temperature along the ~1024 kg/m3 isopycnal holds the greatest correlation (Fig. 2c).

After establishing that subsurface, and not surface, ocean temperatures hold the strongest statistical correlation with aLMI, the authors dedicate Figs. 3-5 to:

i) understanding how the ventilation of subtropical waters regulates subsurface temperature in typhoon alley

ii) how upwelling and turbulent mixing act together to allow the entrainment of deep water temperatures onto the surface mixed layer.

I will center my main comments around three points that caught my attention in the Discussion section.

> *Lines 356-357: The annual peak intensity of TC in the western North Pacific is exclusively highly correlated with the temperature of a subsurface warm and saline water mass [...]*

At surface level, this is an interesting result. However, once I think about it carefully, I struggle to see how this is surprising or how it merits publication in a prestigious journal. The well-known results in Lin et al (2013, *GRL*) already established that the true limiting factor for enthalpy fluxes to a TC is not set by the pre-TC SST, but rather by the likely evolution of SST under the TC. If this result is not obvious from the existing literature, the authors need to do a better job at explaining how this is surprising. The focus on this particular area of the north Pacific is valuable, but it was already addressed in this journal and on a similar fashion by Kang et al (2024, *Nat. Comms.*)

> *Lines 366-368: [...] the strength of super TCs in the western North Pacific is unlikely to increase in the next few decades.*

How exactly is this claim supported by the text? I have many objections. First, subsurface ocean temperatures are warming in much of the global oceans. Second, whether or not the strength of super TCs changes is not only determined by trends in aLMI. One could envision, for example, an increase in the number of TCs with wind speed > 70 m/s, and an increase in the duration over which this intensity is maintained, and still not see a change in aLMI. In this case, the strength of super TCs would change in ways that are profoundly consequential. Overall, this is a

big claim about a highly contested question that, in my opinion, was not granted fair attention and care in the main text.

> *Lines 372-376: Subsurface temperature thus exerts an important impact on the evolution of the passing-by TCs, whereas the influence of pre-TC is effectively suppressed. Such mechanism explains the weak relation between global warming, but it does not mean the global warming have no impact on TCs.*

This is an interesting excerpt. The first half is not a result of this manuscript, but a well-established fact for many decades. The second half argues that the crucial role of subsurface temperatures means that the intensity of super TCs is not modified by oceanic temperatures. This seems to imply that there are no temperature trends in the subsurface warm pool, which was not shown in the main text. Again, the authors are making big claims about a highly debated question without having produced robust tests.

With all this in mind, I recommend that this manuscript be substantially revised. It requires more focus to distinguish itself from previous literature and convincingly answer one or two science questions.

References:

Lin, I.-I., P. Black, J. F. Price, C.-Y. Yang, S. S. Chen, C.-C. Lien, P. Harr, N.-H. Chi, C.-C. Wu, and E. A. D'Asaro (2013), An ocean coupling potential intensity index for tropical cyclones, *Geophys. Res. Lett.*, 40, 1878–1882, doi:10.1002/grl.50091.

Kang, S.K., Kim, SH., Lin, II. et al. The North Equatorial Current and rapid intensification of super typhoons. Nat Commun 15, 1742 (2024). https://doi.org/10.1038/s41467-024-45685-2
